# Whole-Genome Analysis of Histone H3 Lysine 27 Trimethylation in *Arabidopsis*

**DOI:** 10.1371/journal.pbio.0050129

**Published:** 2007-04-17

**Authors:** Xiaoyu Zhang, Oliver Clarenz, Shawn Cokus, Yana V Bernatavichute, Matteo Pellegrini, Justin Goodrich, Steven E Jacobsen

**Affiliations:** 1 Department of Molecular, Cell and Developmental Biology, University of California Los Angeles, Los Angeles, California, United States of America; 2 Institute of Molecular Plant Science, University of Edinburgh, Edinburgh, United Kingdom; 3 School of Biology, University of Edinburgh, Edinburgh, United Kingdom; 4 Molecular Biology Institute, University of California Los Angeles, Los Angeles, California, United States of America; 5 Howard Hughes Medical Institute, University of California Los Angeles, Los Angeles, California, United States of America; Oregon State University, United States of America

## Abstract

Trimethylation of histone H3 lysine 27 (H3K27me3) plays critical roles in regulating animal development, and in several cases, H3K27me3 is also required for the proper expression of developmentally important genes in plants. However, the extent to which H3K27me3 regulates plant genes on a genome-wide scale remains unknown. In addition, it is not clear whether the establishment and spreading of H3K27me3 occur through the same mechanisms in plants and animals. We identified regions containing H3K27me3 in the genome of the flowering plant *Arabidopsis thaliana* using a high-density whole-genome tiling microarray. The results suggest that H3K27me3 is a major silencing mechanism in plants that regulates an unexpectedly large number of genes in *Arabidopsis* (~4,400), and that the maintenance of H3K27me3 is largely independent of other epigenetic pathways, such as DNA methylation or RNA interference. Unlike in animals, where H3K27m3 occupies large genomic regions, in *Arabidopsis,* we found that H3K27m3 domains were largely restricted to the transcribed regions of single genes. Furthermore, unlike in animals systems, H3K27m3 domains were not preferentially associated with low–nucleosome density regions. The results suggest that different mechanisms may underlie the establishment and spreading of H3K27me3 in plants and animals.

## Author summary

During plant and animal development, genes must be activated or repressed according to a strict temporal and spatial schedule. Histones, which are DNA-packaging proteins, play a key role in this process. For development to proceed normally, an amino acid residue (lysine 27) in histone H3 must undergo a chemical modification (called trimethylation). The modified histone (H3K27me3) maintains the repression of its target genes in appropriate tissues or developmental stages. H3K27me3 has been shown to regulate hundreds of genes and many developmental processes in animals, where it also appears to interact with other epigenetic pathways. However, the extent to which this histone modification regulates plant gene expression remained unknown. Does H3K27me3 interact with other epigenetic pathways in plants? Do plants and animals have similar H3K27me3 patterning and underlying mechanisms? To address these questions, we combined chromatin immunoprecipitation with whole-genome tiling microarrays (ChIP-chip) to identify H3K27me3-associated regions across the entire genome of the flowering plant *Arabidopsis* at high resolution (35 base pairs). The results suggest that H3K27me3 is a major and systematic gene silencing mechanism in plants that acts independently of small RNAs or DNA methylation. Furthermore, distinct features of *Arabidopsis* H3K27me3 patterning suggest that different mechanisms may be responsible for the establishment and spread of this histone modification in plants and animals.

## Abstract 3

This paper demonstrates that histone H3 lysine 27 methylation is a major gene-silencing histone modification in plants.

## Introduction

Trimethylation of histone H3 lysine 27 (H3K27me3) is critically important for the normal development of animals. The Polycomb-group (PcG) protein complexes PhoRC, PRC1, and PRC2 collectively establish and maintain H3K27me3 at ~400 and ~2,000 genes in *Drosophila* and mammals, respectively [1–4]. In *Drosophila*, PRC1 and PRC2 are recruited to nucleosome-depleted regions of the genome called Polycomb response elements (PREs) primarily through the sequence-specific binding activity of Pho [1,5–10]. The PRC2 complex then catalyzes the trimethylation of H3K27, whereas the PRC1 complex is required for the bidirectional spreading of H3K27me3 from PREs to the adjacent regions, presumably until an insulator is encountered. As a result, H3K27me3 forms broad domains in *Drosophila* and mammals that can span up to hundreds of kilobases and cover multiple genes, maintaining them in a transcriptionally suppressed state at appropriate developmental stages [1,3,11,12]. The observation that H3K27me3 target genes are enriched for transcription factors underscores the importance of this histone modification in regulating animal development [1–3,11,12].

Plants also contain ample amounts of H3K27me3, accounting for ~5% of the canonical histone H3.1, but trimethylation is undetectable on the histone variant H3.2 (referred to as H3.3 in *Drosophila*) that is predicted to be associated with actively transcribed genes [13,14]. Furthermore, *Arabidopsis* mutants defective in H3K27me3 exhibit severe developmental abnormalities [15–18], and the repression of several important developmental patterning genes in *Arabidopsis*, such as *FLOWERING LOCUS C (FLC), AGAMOUS,* and *MEDEA,* requires H3K27me3 [19–24]. It is therefore likely that the silencing function of H3K27me3 is conserved between plants and animals and that H3K27me3 also plays essential roles in regulating normal plant development.

However, several important questions regarding the patterning and function of H3K27me3 in plants remain unanswered. For example, the extent to which H3K27me3 regulates plant gene expression on a genome-wide scale is unknown. Thus far, only seven plant genes have been shown to be associated with H3K27me3, including *FLC, AGAMOUS, MEDEA, SHOOT MERISTEMLESS (STM), PHERES1, FUSCA3,* and *AGAMOUS-LIKE 19 (AGL19)* [19–27]. In addition, whereas plants have homologs of each of the PRC2 components, they do not encode components of PRC1 or PhoRC, and it is therefore not clear whether H3K27me3 is established and maintained by similar mechanisms in plants and animals [28,29]. Furthermore, the relationship between H3K27me3 and other important epigenetic pathways in plants such as DNA methylation and RNA interference (RNAi) has not been determined.

To begin to address these questions, we identified regions containing H3K27me3 in the *Arabidopsis* genome using high-resolution whole-genome tiling microarrays. We found that H3K27me3 regulates an unexpectedly large number of genes (~4,400) in *Arabidopsis,* including numerous transcription factors. In addition, we present evidence that H3K27me3 functions independently of DNA methylation or RNAi. Furthermore, several important differences were observed between the patterning of H3K27me3 in *Arabidopsis* and *Drosophila,* suggesting that distinct mechanisms may underlie the establishment and maintenance of H3K27me3 in plants and animals.

## Results/Discussion

### Genome-Wide Identification of H3K27me3 Regions in *Arabidopsis*

We performed a genome-wide identification of regions containing H3K27me3 in *Arabidopsis* using chromatin immunoprecipitation (ChIP) and high-density Affymetrix whole-genome tiling microarrays (ChIP-on-chip). Genomic DNA associated with H3K27me3 was isolated by ChIP, amplified, and hybridized to a microarray, which covered ~97% of the *Arabidopsis* genome at 35–base pair (bp) resolution [30]. As a control, nucleosomal DNA was isolated by ChIP using an antibody against the C terminus of H3 (regardless of its modifications) [31]. Genomic regions associated with H3K27me3 (“H3K27me3 regions”) were identified as those yielding significantly higher hybridization signals when probed with H3K27me3 ChIP samples than with nucleosomal DNA, using a two-state hidden Markov model based on probe-level *t* statistics (see Materials and Methods) [32].

**Figure 1:**
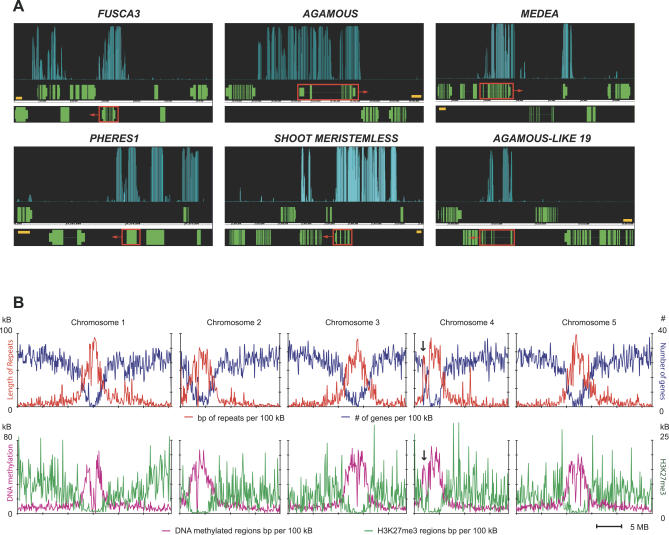
Genome-Wide Identification of H3K27me3 Regions (A) Comparison of ChIP-chip results with *Arabidopsis* genes (red boxes, where arrows indicate direction of transcription) that were previously shown to be H3K27me3 targets [21–27]. Genes are shown as green boxes (exons) and lines (introns), and H3K27me3 is shown as vertical light blue bars [posterior probability ranging from 0 (bottom) to 1 (top)]. Orange scale bars represent 1 kb. (B) Euchromatic chromosomal distribution of H3K27me3 regions. Top panels: the total length of repetitive sequences (*y-*axis, left-side scale) and number of genes per 100 kb (*y-*axis, right-side scale). Bottom panels: the total length of methylated DNA (*y-*axis, left-side scale) and H3K27me3 regions per 100 kb (*y-*axis, right-side scale). Arrows indicate the heterochromatic knob on chromosome 4.

The H3K27me3 regions identified in this way were highly consistent with results from previous studies. All seven known H3K27me3 target genes were found to be enriched for H3K27me3 in our dataset (listed in Figures 1A and S1). In addition, we found that many other genes previously implicated by genetic evidence to contain H3K27me3 (e.g., misregulated in Polycomb-group protein mutant backgrounds) were indeed direct H3K27me3 targets (Figure S1). For example, of the 106 genes overexpressed in an *msi1* mutant (component of PRC2) and therefore likely to be enriched for direct H3K27me3 targets [33], 48 (~45.3%) were associated with H3K27me3 in our dataset, whereas the remaining 58 could represent indirect targets. In contrast, only one region (253 bp) in the 154,478-bp chloroplast genome (~0.16%) was falsely identified as containing H3K27me3. Additional verifications were obtained by real-time PCR validation of H3K27me3-positive and -negative regions identified here, using independently prepared ChIP samples (Figure S2). Taken together, these results indicate that our procedure was sensitive and yielded a low false discovery rate.

A total of 8,979 H3K27me3 regions were identified, covering ~6.9 Mb and representing ~5.7% of the sequenced nuclear genome. Consistent with previous results from cytological studies using immunofluorescence [16], we found that H3K27me3 regions were highly enriched in the euchromatic arms, resembling the distribution of genes (Figure 1B). This is in stark contrast to the heterochromatic distribution of several other silencing marks such as DNA methylation, small interfering RNAs (siRNAs), or H3K9me2 [16,30,34], and suggests that H3K27me3 is primarily targeted to genic regions. Indeed, 6,357 (~70.8%) of the H3K27me3 regions were found in the promoters (200-bp regions upstream of transcription start sites) or the transcribed regions of genes.

### H3K27me3 Targets a Large Number of *Arabidopsis* Genes

H3K27m3 was found to be associated with a large number of genes in *Arabidopsis*. We found that 2,778 of the 14,948 expressed genes with known functions (“known genes”; ~18.6%) and 1,628 of the 10,475 expressed genes with unknown functions (“unknown genes”; ~15.6%) were H3K27me3 targets, as well as many computationally predicted but not expressed genes (“nonexpressed genes”; 249 of 1,116; ~22.3%) and pseudogenes (276 of 3,811; ~7.2%). A list of the H3K27me3 target genes is provided in Dataset S1. This likely represents a conservative estimate of all of the H3K27m3 target genes, because only one developmental stage was assayed here (10-d-old seedlings), and the establishment of H3K27me3 at specific genes may take place at different stages of development in response to developmental or environmental cues [19,20].

**Figure 2:**
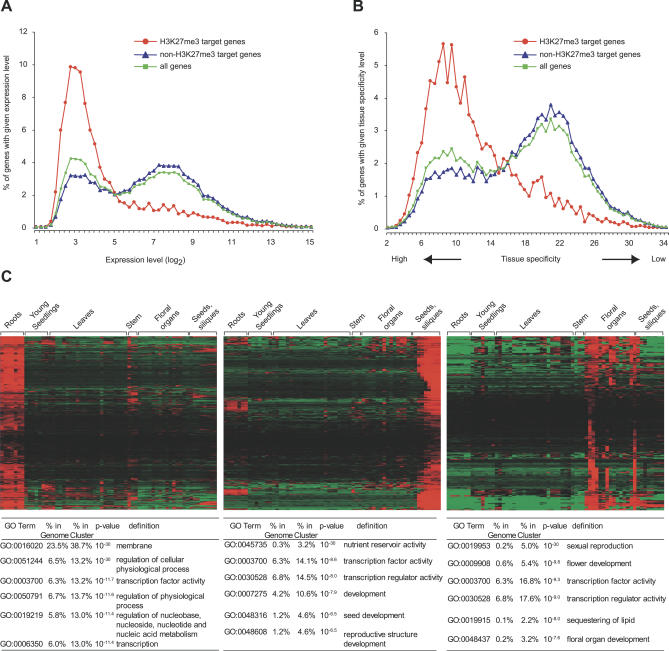
Characteristics of H3K27me3 Target Genes (A) Expression level of H3K27me3 target genes (red) compared to all genes (green) or genes that are not targeted by H3K27me3 (blue). Expression levels were obtained by averaging appropriate developmental stages from a recently published dataset [35] (see Materials and Methods). *x-*axis: expression level (log_2_ scale, vertical bars indicate the bins used.); *y-*axis: percentage of genes with given expression level. (B) Tissue specificity of H3K27me3 target genes (red), non-H3K27me3 target genes (blue), and all genes (green), measured by entropy values (low entropy values = high tissue specificity). *x-*axis: entropy values (vertical bars indicate the bins used); *y* axis: fraction of genes with the given entropy value. (C) Cluster analysis of H3K27me3 target genes. H3K27me3 target genes are grouped into eight mutually exclusive clusters based on their expression patterns. Three clusters that are specifically expressed in roots (left), seeds/siliques (middle), and floral organs (right) are shown as here as examples. Other clusters are shown in Figure S3. Each row represents a gene, and each column represents a tissue type. Red or green indicate tissues in which a particular gene is highly expressed or repressed, respectively. Results of GO analyses for each cluster are shown below. The “% in genome” and “% in cluster” columns indicate the percentages of genes in the genome or in the clusters with corresponding GO terms.

H3K27me3 target genes were analyzed with respect to their expression level and tissue specificity using a previously published expression dataset [35], as well as their functional classifications. Consistent with the function of H3K27me3 in transcriptional silencing, H3K27me3 target genes were expressed at significantly lower levels in young seedlings than those that did not contain H3K27me3 (Figure 2A). Importantly, most H3K27me3 target genes are expressed in a very tissue-specific manner (Figure 2B), as measured by Shannon entropy [36], suggesting that H3K27me3 may facilitate the repression of these genes in appropriate tissues. This finding is further supported by cluster analysis of genes based on their expression patterns. The majority of H3K27me3 target genes were expressed only in one or a few specific tissues, such as floral organs, siliques/seeds, mature leaves, or roots (Figures 2C and S3). With regard to their functions, H3K27me3 targets were highly enriched for genes involved in transcriptional regulation, but also included many other genes with diverse functions (Figures 2C and S3). Taken together, these results suggest that H3K27me3 is a component of a widespread gene silencing system in *Arabidopsis* that is involved in the regulation of numerous genes and many developmental processes.

### H3K27me3 Is Usually Confined to Single Genes in *Arabidopsis*

In animal systems such as *Drosophila* and mammals, the concerted actions of the PRC1 and PRC2 complexes are responsible for the bidirectional spreading of H3K27me3 to sometimes hundreds of kilobases from the PREs [1,3,8,11,12]. Whether a similar spreading process occurs in plants remains unknown, because plants do not encode components of PRC1. Some characteristics of H3K27me3 regions (such as their length) could provide important information to address this question, as a broad distribution of H3K27me3 in *Arabidopsis* comparable to that in *Drosophila* and mammals might indicate similar levels or mechanisms of spreading.

**Figure 3:**
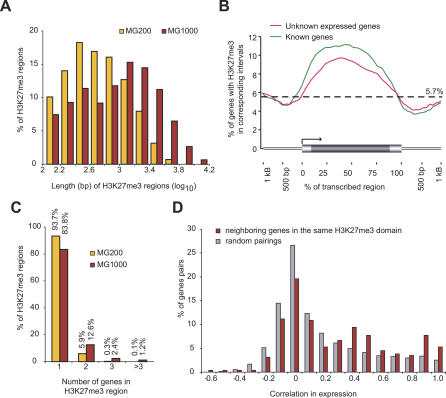
Characteristics of H3K27me3 Regions in *Arabidopsis* (A) Length distribution of H3K27me3 regions [yellow: maximal gap = 200 bp (MG200); brown: maximal gap = 1,000 bp (MG1000); see Materials and Methods for details]. *x-*axis: length of H3K27me3 regions (log_10_ scale); *y-*axis: percentage of H3K27me3 regions with corresponding length. (B) Gene-level distribution of H3K27me3. Each gene (thick horizontal bar, shown as 2.5 kb, which is the average length of *Arabidopsis* genes) was divided into 20 intervals (5% each interval), and the 1-kb regions upstream and downstream of each gene (thin horizontal bars) were divided into 50-bp intervals. The percentage of genes with H3K27me3 in each interval was graphed (*y-*axis). Dotted line indicates the percentage of the *Arabidopsis* genome that is associated with H3K27me3 (~5.7%). (C) The number of genes covered by each H3K27me3 region (yellow: maximal gap = 200 bp; brown: maximal gap = 1,000 bp). *x-*axis: number of genes covered by a H3K27me3 region; *y-*axis: percentage of H3K27me3 regions covering a given number of genes. (D) Correlation of the expression patterns of neighboring H3K27me3 target genes (maximal gap = 1,000 bp) covered by the same H3K27me3 region (brown). All possible pair-wise correlations between H3K27me3 target genes were used as a control (gray). *x-*axis: correlation of expression patterns (1 = perfect positive correlation; −1 = perfect negative correlation; 0 = not correlated). *y-*axis: percentage of gene pairs with corresponding correlation.

We found that H3K27me3 regions in *Arabidopsis* were significantly shorter than those in *Drosophila* and mammals (Figure 3A). Even when we joined adjacent H3K27me3 regions when they were separated by less than 1 kb ("maximal gap” = 1 kb; see Materials and Methods), nearly half (~49.1%) of H3K27me3 regions were still shorter than 1 kb in length (Figure 3A), whereas those in *Drosophila* are usually ~20–50 kb long [1]. In addition, inspection of individual H3K27me3 regions revealed that, in most cases, H3K27me3 regions spanned significant portions of the target genes but rarely extended beyond them into adjacent genes (see Figures 1A and S1 for examples). Mapping of the position of H3K27me3 regions relative to genes showed that H3K27me3 was enriched in the transcribed region with a notable bias toward the 5' end and that the regions immediately upstream of promoters and downstream of the 3' ends of genes had lower levels of H3K27me3 relative to the genome average (Figure 3B). In addition, on a genome-wide scale, the vast majority of H3K27me3 regions (>90%) that overlapped with genes were found to be limited to single genes (Figures 1A, 3C, and S1). Considering that a large number (>17%) of *Arabidopsis* genes were H3K27me3 targets, it is possible that two H3K27me3 regions covering two closely spaced target genes were sometimes inappropriately joined into a single H3K27me3 region during the analysis (because gaps were allowed). Consistent with this possibility, the distances between neighboring genes covered by the same H3K27me3 regions were shorter than the genome average (Figure S4). Furthermore, we reasoned that if two genes were indeed controlled by H3K27me3 in the same region, they may have similar expression patterns. However, although a few exceptions were observed, the overall correlation level between the expression patterns of neighboring genes covered by the same H3K27me3 regions was similar to randomly paired H3K27m3 target genes (Figure 3D). Taken together, these results suggest that in most cases, H3K27me3 is significantly enriched in and limited to single genes, indicating that long-range spreading of H3K27me3 similar to that seen in *Drosophila* may not take place in *Arabidopsis*. However, one interpretation of the relatively broad distribution of H3K27me3 within individual target genes is that limited spreading from an initiation site might also occur in *Arabidopsis*. Among other possibilities, it is interesting to consider that the H3K27me3 methyltransferase(s) might interact with the transcription apparatus, or that insulator-like sites (DNA sequences or epigenetic marks) might border most H3K27m3 target genes.

### H3K27me3 Regions Are Not Preferentially Associated with Low Nucleosome Density Regions in *Arabidopsis*

The Polycomb-group protein complexes in *Drosophila* are recruited to PREs primarily through the sequence-specific binding activity of Pho [5,6], a factor which also appears to be lacking in plants. Recent genomic and biochemical studies have shown that the *Drosophila* PRE regions are depleted of nucleosomes, perhaps to facilitate (or as a result of) the binding of Pho and to accommodate the assembly of the multi-protein complexes PRC1, PCR2, and PhoRC [1,7–10]. To determine whether low nucleosome density (LND) regions of significant length are also associated with H3K27me3 in *Arabidopsis,* we amplified input genomic DNA samples that were not subjected to ChIP, hybridized them to the microarrays, and compared the results to those from hybridization using nucleosomal DNA. LND regions were identified as those yielding significantly higher hybridization signals when probed with input genomic DNA than nucleosomal DNA.

**Figure 4:**
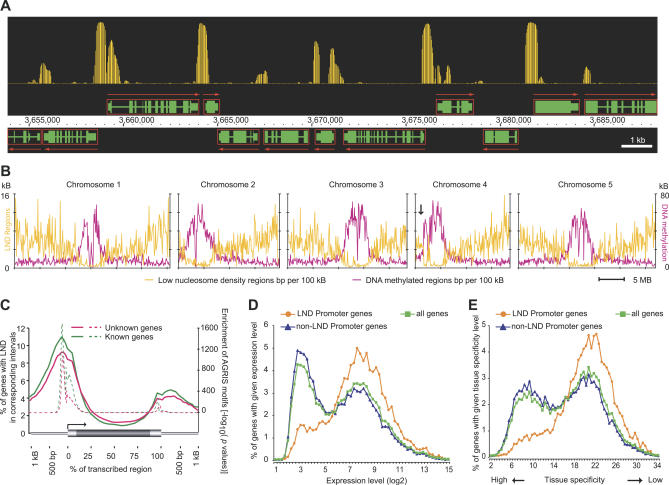
LND Regions in *Arabidopsis* (A) A region of chromosome 1, showing the presence of LND regions (yellow vertical line) in promoters. Genes are shown as in Figure 1A. Red boxes indicate individual genes. Red arrows indicate the direction of transcription. (B) Euchromatic chromosomal distribution of LND regions shown as the total length of LND regions per 100 kb (left *y-*axis). Arrow indicates the heterochromatic knob on chromosome 4. Distribution of DNA methylation is shown for comparison (right *y-*axis). (C) Gene-level distribution of LND regions (solid lines, left *y-*axis) and *Arabidopsis* Gene Regulatory Information Server (AGRIS) transcription factor binding sites (dotted lines, right *y-*axis). *x-*axis is described as in Figure 3B. (D) Expression levels of genes with LND regions in their promoters (LND promoter genes, yellow) compared to non-LND genes (blue), or all genes (green). (E) Tissue specificity (shown as entropy) of LND promoter genes (yellow) compared to non-LND promoter genes (blue), or all genes (green).

A total of 10,104 LND regions were identified, accounting for 4,854,913 bp or ~4.1% of the genome (see Figure 4A for examples). Independent experimental validations were performed using a micrococcal nuclease (MNase) sensitivity assay for 17 randomly selected LND regions; all 17 regions were found to be hypersensitive to MNase digestion (Figure S5). The most common length of LND regions corresponded to the length predicted to be occupied by approximately two nucleosomes with a median of ~410 bp, and most LND regions (~83.6%) were smaller than the length predicted for four nucleosomes (Figure S6). Similar to H3K27me3-containing regions, LND regions were enriched in the euchromatic regions of the genome (Figure 4B).

However, several lines of evidence suggest that H3K27me3 and LND regions are not preferentially associated. First, only ~6.3% of the H3K27me3 regions were located within 180 bp on either side of a LND region. In contrast, ~12.9% of randomly chosen control regions (with similar length and chromosomal distribution as the H3K27me3 regions) were found to be located within 180 bp of a LND region. Second, LND regions were highly enriched in the promoters and 5′ ends and slightly enriched at the 3′ ends, but were depleted in other regions of expressed genes (Figure 4C). Specifically, although only ~4.1% and ~5.1% of the *Arabidopsis* genome consists of LND regions and promoters, respectively, ~42.6% of LND regions were located in promoters, and ~15.4% of promoters contained LND regions. This promoter localization of LND regions is in contrast to H3K27m3 regions, which are enriched in the body of genes (Figure 3B), but is similar to that observed in animals and fungi [31,37–41], indicating that this feature of chromatin organization is conserved in all three eukaryotic kingdoms. Interestingly, the distribution of LND regions is highly similar to the distribution of transcription factor binding sites (Figure 4C). This suggests that many LND regions identified here may be functionally significant, perhaps to facilitate (or are caused by) the binding of transcription factors. We hereafter refer to promoters with LND regions as “low nucleosome density promoters (LND promoters),” and LND promoter–containing genes are listed in Dataset S2. Importantly, the fraction of LND promoter genes that were H3K27me3 targets (350 of 4,785) was significantly lower than genome average. That is, in the majority of cases, genes do not simultaneously contain LND regions in their promoters and H3K27me3. In fact, LND promoter genes were among the most highly expressed genes in *Arabidopsis*, had very low levels of tissue specificity, and were enriched in catalytic enzymes involved in a variety of physiological processes, whereas nonexpressed genes or pseudogenes usually do not have LND promoters (Figures 4D, 4E, S7, and S8). Taken together, these results suggest that LND regions in the *Arabidopsis* genome mark the promoters and 5′ ends of highly transcribed genes and that H3K27m3 regions in *Arabidopsis* do not colocalize with LND regions of significant length.

### H3K27me3 Acts Independently of DNA Methylation and RNAi

**Figure 5:**
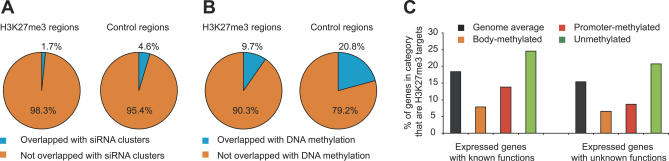
Relationship between H3K27me3, siRNAs, and DNA Methylation (A) The fraction of H3K27me3 regions and random control regions overlapping with siRNA clusters (blue). (B) The fraction of H3K27me3 regions and random control regions overlapping with DNA-methylated regions (blue). (C) The fractions of genes containing body DNA methylation (orange), promoter DNA methylation (red), and no DNA methylation (green) that are H3K27me3 targets. The genome averages of expressed genes are shown for comparison.

DNA methylation and siRNA-mediated silencing pathways represent two major epigenetic silencing mechanisms in plants and in many animal systems. Several recent studies have described potential functional relationships between H3K27me3 and DNA methylation or RNAi [42–44], and we therefore compared the H3K27m3 regions defined here to *Arabidopsis* genomic regions containing DNA methylation or associated with high levels of small RNAs. A deep sequencing study of endogenous siRNAs in *Arabidopsis* showed that siRNAs are generally depleted in the transcribed regions of genes [34]. Consistent with this finding, and also with the genic distribution of H3K27me3 reported here, we found that only ~1.7% of H3K27me3 regions overlapped with siRNA clusters, a fraction lower than randomly selected control regions (~4.6%) (Figure 5A). Moreover, the fraction of microRNA target genes that also contained H3K27me3 was found to be very similar to the genome average (23 of 143, ~16.1%). Thus, it is unlikely that the maintenance of H3K27m3 in *Arabidopsis* requires a persistent targeting mechanism involving siRNAs or microRNAs.

In contrast to the depletion of siRNAs in genic regions, results from recent genome-wide analyses of DNA methylation revealed that genic regions in *Arabidopsis* contain ample DNA methylation; the transcribed regions of roughly one third of expressed genes are DNA methylated in the CG sequence context (“body-methylated genes”) [30,45,46]. It was of particular interest to determine the relationship between H3K27me3 and DNA methylation, because H3K27me3 has been suggested to directly target DNA methylation in mammalian cells [44], but two previously described *Arabidopsis* H3K27me3 targets *(FLC* and *AGAMOUS)* do not appear to be controlled directly by DNA methylation [47,48]. We found that on a genome-wide scale, H3K27me3 regions were significant-ly hypomethylated; only 871 of the 8,979 H3K27me3 regions (~9.7%) contained DNA methylation (~9.1% and ~11.1% for genic and intergenic H3H27me3 regions, respectively), compared with ~20.8% for randomly selected control regions (Figure 5B). The relatively low level of DNA methylation in H3K27m3 regions was not due to a lower CG content, because H3K27me3 regions instead showed higher CG contents than either the genome average or randomly selected control regions (Figure S9). In addition, we found that the fraction of body-DNA methylated genes (those with methylation in the transcribed regions but not the promoters [30]) associated with H3K27me3 was significantly lower than the genome average, whereas genes without DNA methylation were much more likely than average to be H3K27me3 targets (Figure 5C). A detailed analysis revealed that the inverse correlation of H3K27me3 target genes and body-DNA methylated genes was not simply due to the fact that H3K27me3 target genes were generally expressed at low levels, whereas body-methylated genes were generally expressed at high levels, because we observed this relationship at many different gene expression levels (Figure S10). Collectively, these results suggest that the patterning and function of H3K27me3 in *Arabidopsis* are largely independent of DNA methylation.

### Conclusions

We have determined the distribution of H3K27me3 in the *Arabidopsis* genome with 35-bp resolution. This study represents the first genome-wide profiling of histone modification in plants, and the data presented here should be useful for future studies addressing how H3K27m3 regulates individual genes. The entire dataset is available at http://rd.plos.org/pbio.0050129 along with annotations of DNA methylation, gene expression, siRNAs, motif analysis, and related information. The results presented here are consistent with and expand previous findings at individual loci, and suggest that H3K27me3 is a major silencing system that likely acts independently of siRNA-mediated silencing pathways or DNA methylation. At the single developmental stage that we analyzed, 10-d-old seedlings, H3K27me3 targets included ~4,400 or ~17% of expressed genes in *Arabidopsis*. Like in animals, H3K27me3 target genes are enriched for transcription factors, indicating that this histone modification likely plays a widespread role in regulating plant development. Unlike in animals, however, H3K27me3 regions in *Arabidopsis* are shorter, enriched in transcribed regions, and appear to be confined to their target genes. In addition, H3K27me3 regions in *Arabidopsis* do not colocalize with nucleosome-depleted regions of significant length. Collectively, these results suggest that although the silencing function of H3K27me3 is conserved, fundamental differences between plants and animals may exist in the mechanisms by which H3K27me3 is established or maintained.

## Materials and Methods

### ChIP, sample preparation, and microarray hybridization.

ChIP was performed as described [23,49]. Plants were grown under 16 h of light on 1/2 X Murashige and Skoog media, and 1.0–1.5g of whole seedling tissue was harvested and fixed after 10–14 d. Rabbit polyclonal antibodies α-H3K27me3 (rabbit 6523 bleed 5, generous gift of T. Jenuwein) and α-H3 (Abcam number ab1791; http://www.abcam.com) antibodies (2 μg in 100 μl) were incubated for 3–5 h at 4 °C with 25 μl of magnetic protein A beads (Invitrogen number 100.01; http://www.invitrogen.com). The IP was performed as described [23], and DNA was resuspended in 50–75 μl H_2_O. Input DNA and ChIP samples were amplified, labeled, and hybridized to microarrays as described [30]. Four biological replicates were performed for each set of experiments. LND regions were defined as those giving higher signal when probed with the input DNA samples than with nucleosome DNA samples. Whereas LND regions are most likely relatively devoid of nucleosomes (Figure S5), it is also possible that some regions of the chromatin were detected as LND regions because they were less accessible to the H3 antibody utilized. All raw microarray data (CEL files) have been deposited in Gene Expression Omnibus (GEO) (http://www.ncbi.nlm.nih.gov/geo/).

### Microarray data analyses.

Raw microarray data from oligo probes that mapped to unique locations in the genome (representing ~90% of all probes) were quantile normalized and analyzed using Tilemap with the Hidden Markov model option, similar to previously described [30,32]. DNA methylation results shown in Figure 1B were re-analyzed using previously published data, following the same procedure as for H3K27me3 or LND regions [30]. Neighboring probes yielding posterior probabilities of 0.5 or higher were joined into regions by requiring a minimal run of 100 bp and allowing a maximal gap of 200 bp. In addition, for results presented in Figure 3A and C, a maximal gap of 1,000 bp was allowed in a separate analysis.

### Gene expression.

Data used in the analyses of gene expression levels and patterns were from a previous publication reporting the transcriptional profiling of *Arabidopsis* genes across various developmental stages [35]. Gene expression values were quantile normalized and results from the three replicates of each stage were averaged. Results presented in Figures 2A and 4D were derived from 7–14-d-old seedlings, a stage comparable to the plant materials used here. Entropy and gene ontology analyses were performed as described [30]. For cluster analysis, the logarithm of the expression ratio for each gene divided by its mean value across all conditions was computed. This data was then clustered into 8–10 mutually exclusive groups using *K*-means clustering [50]. The genes within each cluster were then hierarchically clustered and displayed in the figures.

### Distribution of putative transcription factor binding sites relative to genes.

The transcription factor binding motifs were downloaded from the *Arabidopsis* Gene Regulatory Information Server (AGRIS) at http://arabidopsis.med.ohio-state.edu/AtcisDB/bindingSiteContent.jsp and mapped to both strands of the genome. Of the 99 motifs, 27 had too few matches in the genome (<18) and were not analyzed further; the remaining 72 motifs had 93 to 413,956 matches, and their locations were used to determine the distribution relative to genes. For each gene, the 1-kb regions upstream and downstream of the transcribed regions were divided into 20 bins (50 bp per bin), and the gene itself is also divided into 20 bins (5% of the length of the gene per bin). For each bin and each motif, a *p*-value was determined as the probability of having at least the observed number of matches to the bin. The *p*-values of all motifs in a bin were then summarized as *p*[motif1]*p[motif2]*...*p[motif72] and plotted in Figure 4C.

### Validation of ChIP-chip results.

For ChIP-chip results on H3K27me3, selected regions that were either H3K27me3-positive or -negative were validated using independently prepared ChIP samples. Real-time PCR reactions were performed using the iQ SYBR Green Supermix (BIO-RAD, http://www.bio-rad.com) and the primers used are listed in Table S1. The PCR parameters were as follows: 1 cycle of 2 min at 95 °C; 40 cycles of 15 s at 95 °C, 30 s at 60 °C, and 30 s at 72 °C; and 1 cycle of 1 min at 95 °C. The enrichment of H3K27me3 was determined as the fold change of H3K27me3 over input or nucleosomal DNA (normalized by the first negative locus in each region).

MNase sensitivity assays were performed as an independent experimental validation of LND regions identified by ChIP-on-chip results. Nuclei were prepared as described with the following modifications [51]. Ten-d-old *Arabidopsis* seedlings were ground to a fine powder in liquid nitrogen and resuspended in modified Honda buffer (HBM, 25 mM Tris, 0.44 M sucrose, 10 mM MgCl_2_,10 mM β-mercaptoethanol, 2 mM spermine, and 0.1% Triton). After homogenization and filtration, plant extract was applied to a 40%/60% Percoll (GE Healthcare; http://www.gehealthcare.com) gradient and centrifuged for 30 min at 2000 revolutions per minute. Nuclei pellet was collected and washed with HBB (HBM without spermine) and HBC (HBB with 20% glycerol). Nuclei were digested with MNase (TaKaRa; http://www.takara-bio.com) for 10 min to mostly mononucleosomes, and DNA was isolated as described [52]. Equal amounts of untreated genomic DNA or MNase-treated DNA were used in real-time PCR using the iQ SYBR Green Supermix (BIO-RAD), and the primers used are listed in Table S2. The PCR parameters were: 1 cycle of 1 min at 95 °C; 40 cycles of 10 s at 95 °C, 15 s at 55 °C, and 20 s at 72 °C, and 1 cycle of 1 min at 72°C. The relative amounts of PCR templates in MNase-treated DNA were determined as the percentage of untreated DNA and shown in Figure S5.

## Supporting Information

Dataset S1 List of H3K27me3 Target Genes in *Arabidopsis*
(65 KB PDF)

Dataset S2 List of *Arabidopsis* Genes with LND Regions in Their Promoters (LND Promoter Genes) (28 KB PDF)

Figure S1 Comparison of ChIP-chip Results with *Arabidopsis* Genes That Were Previously Known or Implicated to Be H3K27me3 Targets Red boxes indicated individual genes and arrows indicate direction of transcription [17,19,20,23,33,53,54]. Note that previous studies have shown the accumulation of H3K27me3 at *FLC,* but only after vernalization (a prolonged exposure to cold which leads to the repression of *FLC* and promotes flowering) [19,20]. Interestingly, *FLC* is constitutively repressed in the ecotype used in this study (WS) even without vernalization, and the observed accumulation of H3K27me3 at this locus is consistent with other experimental evidence (OC and JG, unpublished data). Genes are shown as green boxes and H3K27me3 is shown as vertical light blue bars [posterior probability, ranging from 0 (bottom) to 1 (top)]. Orange bars represent 1 kb. Additional genes are shown in Figure 1A.
(477 KB PDF)

Figure S2 Validation of ChIP-chip Results by Real-Time PCR from Independently Prepared Biological ChIP Replicates ChIP-chip results of three genomic regions are shown in the top panels and labeled as in Figure 1A. Red horizontal bars represent regions assayed by real-time PCR (see Table S1 for coordinates and primer sequences). Real-time PCR results are shown as the fold of enrichment of H3K27me3 over input DNA (middle panels) or H3K27me3 over nucleosomal DNA (H3 ChIP samples; bottom panels).
(85 KB PDF)

Figure S3 Cluster Analysis of H3K27me3 Target Genes H3K27me3 target genes are grouped into eight mutually exclusive clusters based on their expression patterns. Results for three additional clusters are shown in Figure 2. Each row represents a gene, and each column represents a tissue type. Red or green indicate tissues in which a particular gene is highly expressed or repressed, respectively. Results of gene ontology (GO) analyses for each cluster are shown below. The “% in genome” and “% in cluster” columns indicate the percentages of genes in the genome or in the clusters with corresponding GO terms.
(1.6 MB PDF)

Figure S4 Neighboring H3K27me3 Target Genes Covered by the Same H3K27me3 Regions Are More Closely Spaced than Average Neighboring Gene Pairs The length of intergenic regions between neighboring H3K27me3 target genes (brown) are compared to that between all neighboring genes (grey). *x-*axis: intergenic length (negative values indicate overlapping of genes); *y-*axis: percentage of gene pairs with corresponding distance.
(50 KB PDF)

Figure S5 Independent Experimental Validations of LND ChIP-chip Results Using an MNase sensitivity assay LND regions are more sensitive to MNase digestion, and therefore less DNA from LND regions should remain after MNase digestion than from non-LND regions. Equal amounts of DNA extracted from undigested or MNase-digested nuclei were assayed by real-time PCR to measure the relative abundance of 17 randomly selected regions (orange bars) that were found to be LND regions by ChIP-chip (see Table S2 for primer sequences). Two regions that were not found to have LND (regions 7 and 8 in Figure S2) were included as controls (gray bars). For each region, the relative abundance of the amplified region in MNase-digested sample was shown as the percentage of its abundance in undigested samples (*y*-axis).
(275 KB PDF)

Figure S6 The Distribution of the Lengths of LND regions in *Arabidopsis*
The *x-*axis shows the length of LND regions; the *y-*axis is the percentage of LND regions with corresponding length.
(52 KB PDF)

Figure S7 Cluster Analysis of Genes with LND Regions in Their Promoters (“LND Promoter Genes”) LND promoter genes are grouped into ten mutually exclusive clusters based on their expression patterns. Each row represents a gene, and each column represents a tissue type. Red or green indicate tissues in which a particular gene is highly expressed or repressed, respectively. The most enriched GO terms in each cluster are shown below. The “% in genome” and “% in cluster” columns indicate the percentages of genes in the genome or in the clusters with corresponding GO terms.
(1.0 MB PDF)

Figure S8 The Fractions of Expressed Genes with Known Functions, Expressed Genes with Unknown Functions, Computationally Predicted but Not Expressed Genes, and Pseudogenes That Have LND Regions in Their Promoters (Green) (52 KB PDF)

Figure S9 DNA Hypomethylation of H3K27me3 Regions Is Not due to Their Low CG Contents (A) The average CG content of H3K27me3 regions (brown) is higher than that of randomly selected control regions (gray) or the genome average (black). *y-*axis: the number of CG dinucleotides per 100 bp.
(B) The distribution of CG content of H3K27me3 regions (brown) compared to randomly selected control regions (gray). *x-*axis: number of base pairs per 1 CG dinucleotides; *y-*axis: the number of regions with corresponding CG content.
(76 KB PDF)

Figure S10 Genes Containing DNA Methylation Are Less Likely to Be H3K27me3 Targets To exclude the potential bias introduced by gene expression level in this analysis, all expressed genes with known (top) or unknown (bottom) functions were divided into ten bins according to their expression level (bin 1 has the lowest expression level and bin 10, the highest); each bin has an equal number of genes. The percentage of genes that are H3K27me3 targets was then determined for genes that are DNA methylated in their promoters (“promoter-methylated;” red) or transcribed regions (“body-methylated;” orange) and genes that do not contain DNA methylation (“unmethylated;” blue). For all three classes, higher expression levels are correlated with lower likelihoods of being H3K27me3 targets. However, within each bin, unmethylated genes are always more likely to be H3K27me3 targets than promoter-methylated or body-methylated genes.
(61 KB PDF)

Table S1 PCR Primers Used for Validation of H3K27me3 Results (8 KB PDF)

Table S2 PCR Primers Used for Validation of LND Regions (7 KB PDF)

### Accession Numbers

Gene Expression Omnibus (GEO) (http://www.ncbi.nlm.nih.gov/geo/) series accession numbers for construct used are as follows: GSE7064 (H3K27me3 ChIP-chip), GSE7062 (H3 ChIP-chip), and GSE7063 (input DNA).

## Abbreviations

bp: base pair
ChIP: chromatin immunoprecipitation
GO: gene ontology
H3K27me3: histone H3 lysine 27 trimethylation
LND: low nucleosome density
MNase: micrococcal nuclease
PREs: Polycomb response elements
RNAi: RNA interference
siRNA: small interfering RNA
