# Supplementary material for: Whole-Genome Analysis of Histone H3 Lysine 27 Trimethylation in Arabidopsis
Source: PLoS Biol. 2007 Apr 17;5(5):e129. doi: 10.1371/journal.pbio.0050129 (PMC1852588; doi:10.1371/journal.pbio.0050129)
Supplement: Supplementary file 1 [file pbio.0050129.sd001.pdf]

Supplemental Data 1. List of H3K27me3 target genes in *Arabidopsis*.

At1g01030, At1g01183, At1g01190, At1g01250, At1g01390, At1g01410, At1g01420, At1g01580, At1g01690, At1g02040, At1g02065, At1g02070, At1g02190, At1g02205, At1g02310, At1g02335, At1g02340, At1g02360, At1g02390, At1g02460, At1g02580, At1g02620, At1g02770, At1g02790, At1g02810, At1g02813, At1g02940, At1g02950, At1g02980, At1g03020, At1g03106, At1g03400, At1g03410, At1g03445, At1g03490, At1g03495, At1g03710, At1g03720, At1g03790, At1g03800, At1g03810, At1g03820, At1g03840, At1g03880, At1g03890, At1g04150, At1g04180, At1g04220, At1g04250, At1g04330, At1g04370, At1g04445, At1g04450, At1g04500, At1g04540, At1g04600, At1g04660, At1g04670, At1g05070, At1g05080, At1g05230, At1g05280, At1g05290, At1g05310, At1g05420, At1g05450, At1g05580, At1g05660, At1g05800, At1g06080, At1g06090, At1g06100, At1g06135, At1g06270, At1g06280, At1g06320, At1g06330, At1g06340, At1g06350, At1g06360, At1g06800, At1g06810, At1g06980, At1g06990, At1g07000, At1g07320, At1g07430, At1g07640, At1g07645, At1g07720, At1g07725, At1g08080, At1g08090, At1g08100, At1g08135, At1g08140, At1g08150, At1g08160, At1g08290, At1g08320, At1g08430, At1g08440, At1g08465, At1g08670, At1g08860, At1g08990, At1g09030, At1g09090, At1g09240, At1g09280, At1g09350, At1g09360, At1g09380, At1g09480, At1g09483, At1g09486, At1g09500, At1g09550, At1g09665, At1g09890, At1g09930, At1g09932, At1g09935, At1g09960, At1g10010, At1g10220, At1g10240, At1g10250, At1g10260, At1g10380, At1g10385, At1g10480, At1g10490, At1g10585, At1g10590, At1g10620, At1g10670, At1g10880, At1g11080, At1g11150, At1g11230, At1g11250, At1g11370, At1g11460, At1g11470, At1g11545, At1g11580, At1g11600, At1g11610, At1g11670, At1g11700, At1g11740, At1g11765, At1g11770, At1g11806, At1g11850, At1g11920, At1g11980, At1g12010, At1g12030, At1g12064, At1g12070, At1g12100, At1g12110, At1g12170, At1g12180, At1g12190, At1g12200, At1g12260, At1g12560, At1g12570, At1g12630, At1g12660, At1g12805, At1g12810, At1g12950, At1g12960, At1g12980, At1g13200, At1g13290, At1g13300, At1g13400, At1g13420, At1g13430, At1g13605, At1g13609, At1g13610, At1g13620, At1g13670, At1g13710, At1g13810, At1g13820, At1g14040, At1g14110, At1g14120, At1g14220, At1g14240, At1g14250, At1g14450, At1g14540, At1g14550, At1g14580, At1g14600, At1g14700, At1g14760, At1g14880, At1g14930, At1g14940, At1g14950, At1g14960, At1g15140, At1g15310, At1g15320, At1g15330, At1g15360, At1g15540, At1g15590, At1g15620, At1g15630, At1g15640, At1g15680, At1g16390, At1g16400, At1g16410, At1g16530, At1g16900, At1g16910, At1g16930, At1g16940, At1g16950, At1g17000, At1g17010, At1g17200, At1g17310, At1g17420, At1g17700, At1g17710, At1g17770, At1g17800, At1g17950, At1g17960, At1g18100, At1g18140, At1g18220, At1g18230, At1g18410, At1g18570, At1g18710, At1g18760, At1g18770, At1g18780, At1g18810, At1g18860, At1g18940, At1g19060, At1g19230, At1g19250, At1g19390, At1g19415, At1g19420, At1g19560, At1g19610, At1g19620, At1g19640,

At1g19670, At1g19780, At1g19790, At1g19810, At1g19890, At1g19900, At1g20070, At1g20080, At1g20130, At1g20240, At1g20400, At1g20500, At1g20650, At1g20730, At1g20740, At1g20795, At1g20800, At1g20810, At1g20880, At1g20900, At1g20940, At1g20990, At1g21110, At1g21140, At1g21300, At1g21310, At1g21320, At1g21360, At1g21470, At1g21475, At1g21490, At1g21530, At1g21540, At1g21850, At1g21860, At1g21890, At1g21940, At1g21960, At1g21970, At1g21990, At1g22010, At1g22015, At1g22080, At1g22090, At1g22110, At1g22120, At1g22150, At1g22220, At1g22230, At1g22340, At1g22350, At1g22490, At1g22600, At1g22680, At1g22710, At1g22760, At1g22910, At1g22990, At1g23130, At1g23160, At1g23200, At1g23205, At1g23240, At1g23300, At1g23320, At1g23350, At1g23380, At1g23420, At1g23520, At1g23570, At1g23580, At1g23590, At1g23600, At1g23610, At1g23630, At1g23640, At1g23660, At1g23670, At1g23700, At1g23720, At1g23730, At1g23760, At1g23770, At1g24000, At1g24010, At1g24070, At1g24110, At1g24145, At1g24220, At1g24250, At1g24260, At1g24400, At1g24470, At1g24490, At1g24520, At1g24540, At1g24590, At1g24620, At1g24650, At1g25240, At1g25250, At1g25270, At1g25300, At1g25310, At1g25330, At1g25340, At1g25410, At1g25440, At1g25450, At1g25988, At1g26250, At1g26310, At1g26320, At1g26350, At1g26380, At1g26390, At1g26400, At1g26410, At1g26420, At1g26510, At1g26600, At1g26700, At1g26710, At1g26720, At1g26780, At1g26795, At1g26796, At1g26797, At1g26798, At1g26820, At1g26870, At1g26945, At1g27030, At1g27040, At1g27220, At1g27490, At1g27540, At1g27550, At1g27560, At1g27570, At1g27610, At1g27620, At1g27660, At1g27670, At1g27740, At1g27860, At1g27870, At1g28020, At1g28030, At1g28040, At1g28080, At1g28100, At1g28160, At1g28170, At1g28180, At1g28270, At1g28290, At1g28300, At1g28310, At1g28323, At1g28360, At1g28430, At1g28470, At1g28500, At1g28590, At1g28650, At1g28695, At1g28700, At1g29000, At1g29080, At1g29090, At1g29100, At1g29110, At1g29140, At1g29230, At1g29280, At1g29670, At1g29860, At1g29870, At1g30080, At1g30100, At1g30150, At1g30160, At1g30170, At1g30180, At1g30190, At1g30210, At1g30340, At1g30350, At1g30480, At1g30630, At1g30650, At1g30700, At1g30710, At1g30720, At1g30730, At1g30760, At1g30790, At1g30795, At1g30840, At1g30920, At1g30921, At1g30925, At1g30930, At1g30935, At1g30940, At1g30945, At1g30950, At1g30960, At1g30990, At1g31000, At1g31050, At1g31060, At1g31090, At1g31240, At1g31260, At1g31310, At1g31355, At1g31520, At1g31530, At1g31550, At1g31620, At1g31630, At1g31670, At1g31680, At1g31690, At1g31710, At1g31720, At1g31772, At1g31983, At1g31990, At1g32020, At1g32030, At1g32050, At1g32100, At1g32140, At1g32240, At1g32280, At1g32350, At1g32420, At1g32430, At1g32450, At1g32650, At1g32660, At1g32710, At1g32770, At1g32780, At1g32850, At1g32975, At1g32980, At1g33020, At1g33070, At1g33080, At1g33090, At1g33100, At1g33110, At1g33280, At1g33530, At1g33540, At1g33590, At1g33600, At1g33670, At1g33710, At1g33720, At1g33730, At1g33740, At1g33750, At1g33760, At1g33790, At1g33811, At1g33817, At1g33820, At1g33830, At1g33850, At1g33870, At1g33880,

At1g33890, At1g33900, At1g33910, At1g33920, At1g33930, At1g33960,
At1g34047, At1g34050, At1g34140, At1g34460, At1g34480, At1g34490,
At1g34500, At1g34510, At1g34520, At1g34540, At1g34580, At1g34590,
At1g34650, At1g34670, At1g34690, At1g34790, At1g34842, At1g35140,
At1g35170, At1g35180, At1g35183, At1g35186, At1g35250, At1g35260,
At1g35270, At1g35290, At1g35310, At1g35330, At1g35360, At1g35365,
At1g35440, At1g35450, At1g35550, At1g35610, At1g35630, At1g35690,
At1g35830, At1g35910, At1g36105, At1g36150, At1g36180, At1g36580,
At1g36730, At1g37140, At1g40105, At1g40113, At1g42924, At1g43005,
At1g43010, At1g43020, At1g43130, At1g43140, At1g43640, At1g43650,
At1g43800, At1g44050, At1g44080, At1g44090, At1g44180, At1g44190,
At1g44222, At1g44286, At1g44318, At1g44350, At1g44478, At1g44542,
At1g44800, At1g44940, At1g45060, At1g45063, At1g45100, At1g45201,
At1g46264, At1g46408, At1g46480, At1g46552, At1g46840, At1g46912,
At1g47300, At1g47317, At1g47320, At1g47350, At1g47370, At1g47390,
At1g47395, At1g47400, At1g47405, At1g47485, At1g47510, At1g47540,
At1g47590, At1g47600, At1g47603, At1g47606, At1g47610, At1g47620,
At1g47630, At1g47670, At1g47705, At1g47730, At1g47770, At1g47780,
At1g47786, At1g47790, At1g47800, At1g47810, At1g47860, At1g47915,
At1g47920, At1g47980, At1g47990, At1g48000, At1g48130, At1g48140,
At1g48290, At1g48590, At1g48690, At1g48700, At1g48730, At1g48740,
At1g48742, At1g48800, At1g48910, At1g48990, At1g49030, At1g49100,
At1g49110, At1g49120, At1g49190, At1g49250, At1g49270, At1g49290,
At1g49310, At1g49320, At1g49330, At1g49430, At1g49450, At1g49470,
At1g49570, At1g49770, At1g49860, At1g49960, At1g50050, At1g50090,
At1g50130, At1g50650, At1g50690, At1g50720, At1g50870, At1g50880,
At1g50960, At1g50980, At1g50990, At1g51000, At1g51010, At1g51030,
At1g51040, At1g51050, At1g51150, At1g51175, At1g51190, At1g51220,
At1g51230, At1g51240, At1g51250, At1g51260, At1g51270, At1g51280,
At1g51290, At1g51300, At1g51330, At1g51355, At1g51360, At1g51460,
At1g51470, At1g51480, At1g51490, At1g51790, At1g51805, At1g51810,
At1g51820, At1g51830, At1g51840, At1g51850, At1g51860, At1g51880,
At1g51900, At1g51910, At1g51915, At1g52000, At1g52040, At1g52050,
At1g52060, At1g52070, At1g52100, At1g52110, At1g52120, At1g52130,
At1g52140, At1g52190, At1g52210, At1g52400, At1g52440, At1g52460,
At1g52470, At1g52480, At1g52490, At1g52510, At1g52680, At1g52690,
At1g52695, At1g52700, At1g52830, At1g52840, At1g52890, At1g52900,
At1g52940, At1g53000, At1g53020, At1g53080, At1g53165, At1g53260,
At1g53265, At1g53325, At1g53340, At1g53360, At1g53370, At1g53420,
At1g53600, At1g53610, At1g53620, At1g53640, At1g53690, At1g53700,
At1g53820, At1g53830, At1g53990, At1g54000, At1g54010, At1g54020,
At1g54040, At1g54200, At1g54300, At1g54310, At1g54330, At1g54360,
At1g54480, At1g54530, At1g54550, At1g54560, At1g54575, At1g54620,
At1g54660, At1g54860, At1g54870, At1g54880, At1g54890, At1g54930,
At1g54940, At1g54950, At1g54955, At1g54970, At1g54980, At1g55030,
At1g55035, At1g55110, At1g55220, At1g55230, At1g55240, At1g55260,

At1g55380, At1g55390, At1g55410, At1g55420, At1g55430, At1g55440,
At1g55560, At1g55570, At1g55580, At1g55600, At1g55650, At1g55660,
At1g55700, At1g55740, At1g56030, At1g56040, At1g56100, At1g56160,
At1g56250, At1g56360, At1g56390, At1g56400, At1g56410, At1g56480,
At1g56490, At1g56520, At1g56540, At1g56550, At1g56553, At1g56630,
At1g56650, At1g56680, At1g57565, At1g57570, At1g57580, At1g57650,
At1g57670, At1g57690, At1g57740, At1g57750, At1g57800, At1g57810,
At1g57830, At1g58020, At1g58037, At1g58050, At1g58090, At1g58260,
At1g58265, At1g58270, At1g58280, At1g58300, At1g58310, At1g58330,
At1g58340, At1g58390, At1g58410, At1g58450, At1g58460, At1g59500,
At1g59530, At1g59535, At1g59680, At1g59690, At1g59722, At1g59725,
At1g59740, At1g59780, At1g59790, At1g59800, At1g59810, At1g59940,
At1g59950, At1g60040, At1g60050, At1g60060, At1g60075, At1g60095,
At1g60180, At1g60240, At1g60250, At1g60290, At1g60300, At1g60310,
At1g60320, At1g60340, At1g60350, At1g60360, At1g60370, At1g60380,
At1g60390, At1g60400, At1g60410, At1g60570, At1g60580, At1g60830,
At1g60930, At1g60980, At1g60983, At1g60985, At1g61070, At1g61080,
At1g61105, At1g61110, At1g61120, At1g61130, At1g61270, At1g61320,
At1g61330, At1g61370, At1g61540, At1g61563, At1g61590, At1g61630,
At1g61650, At1g61660, At1g61680, At1g61700, At1g61710, At1g61750,
At1g61810, At1g61830, At1g61840, At1g62210, At1g62220, At1g62225,
At1g62270, At1g62300, At1g62340, At1g62360, At1g62440, At1g62450,
At1g62490, At1g62500, At1g62510, At1g62530, At1g62540, At1g62560,
At1g62570, At1g62700, At1g62710, At1g62760, At1g62940, At1g62975,
At1g62980, At1g63040, At1g63055, At1g63105, At1g63190, At1g63200,
At1g63205, At1g63450, At1g63600, At1g63710, At1g63870, At1g63880,
At1g63910, At1g64000, At1g64030, At1g64035, At1g64060, At1g64255,
At1g64290, At1g64340, At1g64480, At1g64590, At1g64620, At1g64660,
At1g64780, At1g64870, At1g64900, At1g64910, At1g64930, At1g64940,
At1g65050, At1g65180, At1g65330, At1g65340, At1g65342, At1g65345,
At1g65350, At1g65352, At1g65360, At1g65365, At1g65370, At1g65385,
At1g65445, At1g65450, At1g65480, At1g65500, At1g65570, At1g65620,
At1g65630, At1g65640, At1g65680, At1g65740, At1g65750, At1g65760,
At1g65770, At1g65780, At1g65850, At1g65860, At1g65870, At1g65875,
At1g65880, At1g65890, At1g65910, At1g65985, At1g65990, At1g66000,
At1g66010, At1g66020, At1g66045, At1g66060, At1g66120, At1g66130,
At1g66210, At1g66220, At1g66270, At1g66280, At1g66290, At1g66300,
At1g66310, At1g66320, At1g66360, At1g66370, At1g66380, At1g66390,
At1g66420, At1g66440, At1g66470, At1g66480, At1g66490, At1g66500,
At1g66540, At1g66570, At1g66630, At1g66640, At1g66650, At1g66690,
At1g66710, At1g66720, At1g66770, At1g66780, At1g66783, At1g66790,
At1g66795, At1g66800, At1g66850, At1g66860, At1g66950, At1g66960,
At1g66970, At1g67030, At1g67100, At1g67110, At1g67130, At1g67150,
At1g67160, At1g67170, At1g67260, At1g67270, At1g67330, At1g67450,
At1g67455, At1g67560, At1g67640, At1g67710, At1g67760, At1g67770,
At1g67860, At1g67865, At1g67870, At1g68030, At1g68040, At1g68130,

At1g68140, At1g68170, At1g68180, At1g68250, At1g68270, At1g68280,
At1g68320, At1g68360, At1g68380, At1g68390, At1g68450, At1g68460,
At1g68470, At1g68480, At1g68510, At1g68520, At1g68530, At1g68550,
At1g68600, At1g68610, At1g68620, At1g68750, At1g68800, At1g68810,
At1g69080, At1g69090, At1g69100, At1g69110, At1g69120, At1g69140,
At1g69150, At1g69160, At1g69170, At1g69180, At1g69190, At1g69260,
At1g69310, At1g69320, At1g69450, At1g69560, At1g69630, At1g69650,
At1g69660, At1g69690, At1g69810, At1g69830, At1g69860, At1g69870,
At1g69880, At1g69920, At1g69930, At1g69940, At1g69970, At1g70080,
At1g70170, At1g70250, At1g70260, At1g70270, At1g70360, At1g70380,
At1g70390, At1g70510, At1g70540, At1g70560, At1g70640, At1g70670,
At1g70720, At1g70830, At1g70840, At1g70850, At1g70860, At1g70870,
At1g70880, At1g70885, At1g70890, At1g70895, At1g70910, At1g70920,
At1g70960, At1g70970, At1g71000, At1g71050, At1g71160, At1g71280,
At1g71290, At1g71300, At1g71390, At1g71680, At1g71691, At1g71692,
At1g71695, At1g71880, At1g71890, At1g71930, At1g72080, At1g72100,
At1g72110, At1g72120, At1g72140, At1g72200, At1g72210, At1g72230,
At1g72290, At1g72570, At1g72580, At1g73000, At1g73010, At1g73220,
At1g73260, At1g73270, At1g73280, At1g73330, At1g73410, At1g73490,
At1g73510, At1g73530, At1g73590, At1g73610, At1g73810, At1g73850,
At1g73870, At1g74000, At1g74010, At1g74080, At1g74110, At1g74180,
At1g74460, At1g74500, At1g74620, At1g74660, At1g74760, At1g74820,
At1g75040, At1g75160, At1g75240, At1g75250, At1g75410, At1g75450,
At1g75470, At1g75520, At1g75620, At1g75710, At1g75720, At1g75790,
At1g75830, At1g75870, At1g75880, At1g75890, At1g75900, At1g75910,
At1g75920, At1g75930, At1g75940, At1g76210, At1g76220, At1g76230,
At1g76240, At1g76250, At1g76290, At1g76410, At1g76420, At1g76500,
At1g76610, At1g76800, At1g76850, At1g77110, At1g77145, At1g77150,
At1g77160, At1g77200, At1g77650, At1g77655, At1g77690, At1g77700,
At1g77780, At1g77815, At1g77820, At1g77830, At1g77950, At1g77990,
At1g78330, At1g78450, At1g78460, At1g78470, At1g78480, At1g78490,
At1g78500, At1g78640, At1g78720, At1g78740, At1g78750, At1g78760,
At1g78810, At1g78815, At1g78840, At1g78970, At1g78980, At1g79050,
At1g79250, At1g79360, At1g79370, At1g79400, At1g79430, At1g79450,
At1g79520, At1g79580, At1g79650, At1g79680, At1g79760, At1g79770,
At1g79800, At1g79820, At1g79840, At1g79860, At1g80090, At1g80100,
At1g80110, At1g80130, At1g80220, At1g80240, At1g80340, At1g80390,
At1g80540, At1g80580, At1g80760, At1g80865, At2g01430, At2g01500,
At2g01520, At2g01530, At2g01560, At2g01570, At2g01610, At2g01780,
At2g01800, At2g01810, At2g01890, At2g01900, At2g01940, At2g01960,
At2g01990, At2g02000, At2g02010, At2g02070, At2g02080, At2g02120,
At2g02130, At2g02135, At2g02260, At2g02310, At2g02320, At2g02330,
At2g02340, At2g02450, At2g02540, At2g02610, At2g02620, At2g02630,
At2g02640, At2g02650, At2g02660, At2g02670, At2g02680, At2g02690,
At2g02770, At2g02890, At2g03000, At2g03020, At2g03160, At2g03170,
At2g03180, At2g03190, At2g03250, At2g03290, At2g03320, At2g03330,

At2g03360, At2g03500, At2g03550, At2g03560, At2g03690, At2g03700, At2g03710, At2g03720, At2g03730, At2g03740, At2g03840, At2g03850, At2g03860, At2g03900, At2g03960, At2g03970, At2g04025, At2g04032, At2g04046, At2g04047, At2g04050, At2g04090, At2g04100, At2g04150, At2g04160, At2g04200, At2g04210, At2g04300, At2g04495, At2g04500, At2g04680, At2g04810, At2g04820, At2g04830, At2g04840, At2g04910, At2g04920, At2g04930, At2g05185, At2g05190, At2g05200, At2g05380, At2g05400, At2g05410, At2g05420, At2g05430, At2g05435, At2g05440, At2g05500, At2g05510, At2g05530, At2g05580, At2g05850, At2g07080, At2g07120, At2g07130, At2g07570, At2g07688, At2g07783, At2g07786, At2g07787, At2g07792, At2g07981, At2g09862, At2g10930, At2g11005, At2g12190, At2g12200, At2g12462, At2g12510, At2g12646, At2g12875, At2g13630, At2g13810, At2g13820, At2g13900, At2g13910, At2g13920, At2g14070, At2g14210, At2g14220, At2g14245, At2g14280, At2g14282, At2g14290, At2g14440, At2g14510, At2g14540, At2g14550, At2g14560, At2g14605, At2g14610, At2g14620, At2g14670, At2g14710, At2g14760, At2g14920, At2g14960, At2g14990, At2g15010, At2g15020, At2g15080, At2g15090, At2g15130, At2g15350, At2g15390, At2g15400, At2g15590, At2g15610, At2g15660, At2g15670, At2g15770, At2g15780, At2g15840, At2g16005, At2g16120, At2g16130, At2g16190, At2g16220, At2g16230, At2g16290, At2g16310, At2g16365, At2g16570, At2g16730, At2g16840, At2g16850, At2g16890, At2g16895, At2g16960, At2g16970, At2g16980, At2g16990, At2g17050, At2g17055, At2g17060, At2g17070, At2g17090, At2g17310, At2g17470, At2g17525, At2g17590, At2g17600, At2g17680, At2g17780, At2g17785, At2g17940, At2g17950, At2g17960, At2g18050, At2g18060, At2g18070, At2g18080, At2g18120, At2g18140, At2g18150, At2g18190, At2g18193, At2g18230, At2g18320, At2g18360, At2g18370, At2g18380, At2g18450, At2g18460, At2g18480, At2g18490, At2g18550, At2g18570, At2g18590, At2g18660, At2g18720, At2g18810, At2g18870, At2g18880, At2g19000, At2g19010, At2g19020, At2g19040, At2g19050, At2g19060, At2g19070, At2g19150, At2g19200, At2g19310, At2g19320, At2g19500, At2g19610, At2g19630, At2g19640, At2g19660, At2g19770, At2g19890, At2g19893, At2g19980, At2g19990, At2g20080, At2g20100, At2g20150, At2g20160, At2g20170, At2g20350, At2g20430, At2g20520, At2g20530, At2g20570, At2g20620, At2g20635, At2g20800, At2g20805, At2g20825, At2g20875, At2g21000, At2g21010, At2g21100, At2g21140, At2g21220, At2g21235, At2g21260, At2g21490, At2g21510, At2g21720, At2g21780, At2g21870, At2g21900, At2g21910, At2g21920, At2g21930, At2g21980, At2g21990, At2g22030, At2g22040, At2g22050, At2g22060, At2g22150, At2g22180, At2g22190, At2g22290, At2g22330, At2g22340, At2g22510, At2g22540, At2g22590, At2g22620, At2g22630, At2g22750, At2g22760, At2g22800, At2g22810, At2g22980, At2g23010, At2g23130, At2g23160, At2g23170, At2g23220, At2g23230, At2g23240, At2g23260, At2g23330, At2g23440, At2g23510, At2g23540, At2g23590, At2g23670, At2g23760, At2g23800, At2g23850, At2g23860, At2g23870, At2g23880, At2g23960, At2g24000, At2g24010, At2g24080, At2g24210, At2g24255,

At2g24260, At2g24430, At2g24470, At2g24510, At2g24620, At2g24696,
At2g24700, At2g24710, At2g24720, At2g24740, At2g24750, At2g24810,
At2g24820, At2g24850, At2g25095, At2g25120, At2g25150, At2g25160,
At2g25230, At2g25240, At2g25330, At2g25340, At2g25360, At2g25410,
At2g25440, At2g25450, At2g25510, At2g25630, At2g25687, At2g25700,
At2g25770, At2g25780, At2g25790, At2g25810, At2g25820, At2g25880,
At2g25890, At2g25980, At2g26010, At2g26020, At2g26110, At2g26130,
At2g26160, At2g26250, At2g26270, At2g26290, At2g26390, At2g26410,
At2g26560, At2g26580, At2g26695, At2g26700, At2g26720, At2g26850,
At2g26870, At2g26880, At2g26940, At2g26950, At2g26960, At2g27070,
At2g27110, At2g27120, At2g27220, At2g27240, At2g27250, At2g27370,
At2g27385, At2g27550, At2g27560, At2g27570, At2g27660, At2g27670,
At2g27700, At2g27740, At2g27750, At2g27780, At2g27880, At2g27935,
At2g27940, At2g27990, At2g28010, At2g28020, At2g28030, At2g28056,
At2g28085, At2g28170, At2g28180, At2g28210, At2g28220, At2g28280,
At2g28330, At2g28340, At2g28490, At2g28510, At2g28580, At2g28590,
At2g28600, At2g28610, At2g28625, At2g28630, At2g28680, At2g28700,
At2g28710, At2g28770, At2g28780, At2g28790, At2g28810, At2g28860,
At2g28880, At2g28950, At2g28960, At2g28970, At2g28980, At2g28990,
At2g29000, At2g29010, At2g29040, At2g29090, At2g29100, At2g29110,
At2g29120, At2g29125, At2g29130, At2g29210, At2g29220, At2g29250,
At2g29320, At2g29370, At2g29380, At2g29590, At2g29600, At2g29605,
At2g29610, At2g29780, At2g29800, At2g29810, At2g29820, At2g29830,
At2g29840, At2g29860, At2g29930, At2g30090, At2g30130, At2g30210,
At2g30220, At2g30240, At2g30300, At2g30310, At2g30340, At2g30370,
At2g30650, At2g30660, At2g30670, At2g30680, At2g30750, At2g30760,
At2g30770, At2g30890, At2g30900, At2g31081, At2g31082, At2g31083,
At2g31085, At2g31160, At2g31215, At2g31230, At2g31425, At2g31430,
At2g31460, At2g31470, At2g31690, At2g31720, At2g31760, At2g31770,
At2g31780, At2g31910, At2g31920, At2g31930, At2g32270, At2g32275,
At2g32370, At2g32460, At2g32530, At2g32540, At2g32610, At2g32620,
At2g32830, At2g32860, At2g32870, At2g32880, At2g32890, At2g32900,
At2g32960, At2g33070, At2g33160, At2g33180, At2g33385, At2g33655,
At2g33660, At2g33710, At2g33775, At2g33780, At2g33790, At2g33850,
At2g33870, At2g33880, At2g34100, At2g34140, At2g34185, At2g34210,
At2g34315, At2g34350, At2g34360, At2g34370, At2g34530, At2g34540,
At2g34700, At2g34710, At2g34790, At2g34810, At2g34820, At2g34830,
At2g34880, At2g35070, At2g35080, At2g35270, At2g35300, At2g35350,
At2g35570, At2g35580, At2g35585, At2g35590, At2g35612, At2g35640,
At2g35700, At2g35770, At2g35930, At2g35960, At2g35970, At2g35980,
At2g36020, At2g36090, At2g36100, At2g36110, At2g36120, At2g36190,
At2g36255, At2g36260, At2g36330, At2g36440, At2g36450, At2g36560,
At2g36640, At2g36690, At2g36770, At2g36780, At2g36970, At2g36985,
At2g37010, At2g37435, At2g37440, At2g37590, At2g37610, At2g37700,
At2g37720, At2g37730, At2g37740, At2g37800, At2g37810, At2g37820,
At2g37830, At2g37960, At2g38080, At2g38110, At2g38220, At2g38230,

At2g38240, At2g38300, At2g38320, At2g38380, At2g38390, At2g38510, At2g38520, At2g38530, At2g38540, At2g38590, At2g38900, At2g38905, At2g38910, At2g38920, At2g39010, At2g39030, At2g39040, At2g39060, At2g39175, At2g39200, At2g39240, At2g39250, At2g39310, At2g39320, At2g39330, At2g39410, At2g39415, At2g39490, At2g39510, At2g39520, At2g39530, At2g39790, At2g39820, At2g39830, At2g39850, At2g39855, At2g40050, At2g40060, At2g40170, At2g40180, At2g40210, At2g40220, At2g40260, At2g40370, At2g40390, At2g40435, At2g40440, At2g40450, At2g40470, At2g40475, At2g40500, At2g40520, At2g40560, At2g40740, At2g40750, At2g40900, At2g40910, At2g40990, At2g40995, At2g41130, At2g41260, At2g41270, At2g41280, At2g41290, At2g41360, At2g41370, At2g41390, At2g41400, At2g41600, At2g41610, At2g41850, At2g41860, At2g41910, At2g41940, At2g42000, At2g42090, At2g42100, At2g42170, At2g42180, At2g42410, At2g42430, At2g42440, At2g42460, At2g42470, At2g42480, At2g42540, At2g42560, At2g42610, At2g42720, At2g42730, At2g42830, At2g42840, At2g42990, At2g43000, At2g43220, At2g43270, At2g43470, At2g43510, At2g43570, At2g43590, At2g43600, At2g43610, At2g43660, At2g43670, At2g43860, At2g43870, At2g43880, At2g43960, At2g44030, At2g44065, At2g44070, At2g44240, At2g44250, At2g44320, At2g44340, At2g44460, At2g44490, At2g44540, At2g44550, At2g44560, At2g44570, At2g44580, At2g44600, At2g44745, At2g44810, At2g44930, At2g44990, At2g45110, At2g45130, At2g45135, At2g45190, At2g45220, At2g45230, At2g45360, At2g45400, At2g45406, At2g45410, At2g45420, At2g45430, At2g45650, At2g45660, At2g45760, At2g45830, At2g45970, At2g46300, At2g46495, At2g46560, At2g46590, At2g46630, At2g46640, At2g46720, At2g46730, At2g46740, At2g46750, At2g46760, At2g46770, At2g46870, At2g46880, At2g46970, At2g46990, At2g47030, At2g47040, At2g47050, At2g47120, At2g47260, At2g47280, At2g47460, At2g47520, At2g47530, At2g47540, At2g47550, At2g47810, At2g47870, At2g47880, At2g47950, At2g48130, At3g01010, At3g01015, At3g01020, At3g01080, At3g01085, At3g01190, At3g01220, At3g01230, At3g01240, At3g01250, At3g01260, At3g01270, At3g01323, At3g01420, At3g01490, At3g01700, At3g01750, At3g01900, At3g01940, At3g01950, At3g01960, At3g02000, At3g02140, At3g02150, At3g02240, At3g02310, At3g02440, At3g02610, At3g02620, At3g02670, At3g02710, At3g02810, At3g02940, At3g02960, At3g02970, At3g02980, At3g03200, At3g03230, At3g03240, At3g03260, At3g03280, At3g03290, At3g03340, At3g03530, At3g03660, At3g03670, At3g03760, At3g04060, At3g04100, At3g04160, At3g04170, At3g04180, At3g04190, At3g04220, At3g04270, At3g04280, At3g04290, At3g04320, At3g04330, At3g04360, At3g04370, At3g04510, At3g04520, At3g04530, At3g04570, At3g04810, At3g04990, At3g05155, At3g05300, At3g05400, At3g05450, At3g05860, At3g05950, At3g05960, At3g06010, At3g06020, At3g06120, At3g06230, At3g06390, At3g06435, At3g06460, At3g06470, At3g06490, At3g06970, At3g06990, At3g07000, At3g07250, At3g07820, At3g07830, At3g07840, At3g07850, At3g07860, At3g07970, At3g08040, At3g08490, At3g08500, At3g08860, At3g09030, At3g09220, At3g09290,

At3g09340, At3g09385, At3g09400, At3g09520, At3g09922, At3g09925, At3g09930, At3g09940, At3g09990, At3g10000, At3g10020, At3g10116, At3g10280, At3g10340, At3g10500, At3g10575, At3g10580, At3g10585, At3g10590, At3g10595, At3g10600, At3g10750, At3g10780, At3g10790, At3g10815, At3g10845, At3g10890, At3g10900, At3g10990, At3g11060, At3g11150, At3g11160, At3g11180, At3g11260, At3g11300, At3g11310, At3g11325, At3g11385, At3g11390, At3g11402, At3g11410, At3g11430, At3g11480, At3g11580, At3g11750, At3g11920, At3g12000, At3g12143, At3g12203, At3g12410, At3g12420, At3g12430, At3g12440, At3g12450, At3g12460, At3g12470, At3g12500, At3g12710, At3g12720, At3g12730, At3g12850, At3g12890, At3g12900, At3g12910, At3g13130, At3g13140, At3g13228, At3g13229, At3g13280, At3g13370, At3g13400, At3g13490, At3g13540, At3g13590, At3g13624, At3g13630, At3g13650, At3g13662, At3g13720, At3g13730, At3g13784, At3g13790, At3g13810, At3g13830, At3g13890, At3g13950, At3g13960, At3g14040, At3g14067, At3g14100, At3g14110, At3g14210, At3g14250, At3g14260, At3g14300, At3g14370, At3g14480, At3g14490, At3g14500, At3g14510, At3g14520, At3g14780, At3g14820, At3g14880, At3g15280, At3g15370, At3g15510, At3g15605, At3g15670, At3g15680, At3g15800, At3g15830, At3g15870, At3g15910, At3g15940, At3g15960, At3g16330, At3g16340, At3g16360, At3g16370, At3g16380, At3g16390, At3g16400, At3g16410, At3g16420, At3g16430, At3g16440, At3g16450, At3g16460, At3g16510, At3g16540, At3g16550, At3g16552, At3g16580, At3g16590, At3g16660, At3g16670, At3g16680, At3g16690, At3g16830, At3g16920, At3g16960, At3g17060, At3g17070, At3g17080, At3g17110, At3g17150, At3g17180, At3g17185, At3g17200, At3g17203, At3g17260, At3g17265, At3g17270, At3g17320, At3g17480, At3g17490, At3g17500, At3g17530, At3g17540, At3g17550, At3g17560, At3g17570, At3g17730, At3g18010, At3g18280, At3g18330, At3g18400, At3g18450, At3g18460, At3g18470, At3g18540, At3g18550, At3g18560, At3g18980, At3g19055, At3g19070, At3g19160, At3g19200, At3g19210, At3g19270, At3g19300, At3g19380, At3g19430, At3g19700, At3g19710, At3g19880, At3g19890, At3g19920, At3g19940, At3g20160, At3g20210, At3g20360, At3g20362, At3g20365, At3g20460, At3g20470, At3g20550, At3g20555, At3g20557, At3g20580, At3g20640, At3g20705, At3g20710, At3g20750, At3g20760, At3g20840, At3g20850, At3g20880, At3g20885, At3g20890, At3g20975, At3g20980, At3g20990, At3g20993, At3g21010, At3g21020, At3g21030, At3g21040, At3g21050, At3g21090, At3g21200, At3g21210, At3g21340, At3g21360, At3g21370, At3g21380, At3g21410, At3g21420, At3g21690, At3g21710, At3g21720, At3g21730, At3g21760, At3g21790, At3g21800, At3g21830, At3g21840, At3g21850, At3g21860, At3g21870, At3g21880, At3g21900, At3g21910, At3g21960, At3g21965, At3g21970, At3g21980, At3g21985, At3g21990, At3g22050, At3g22053, At3g22057, At3g22060, At3g22080, At3g22120, At3g22133, At3g22140, At3g22232, At3g22237, At3g22250, At3g22340, At3g22350, At3g22400, At3g22490, At3g22500, At3g22640, At3g22650, At3g22710, At3g22720, At3g22730, At3g22740, At3g22760, At3g22770, At3g22800, At3g22820,

At3g22830, At3g22840, At3g22860, At3g22870, At3g22886, At3g22920,
At3g23050, At3g23060, At3g23100, At3g23110, At3g23120, At3g23130,
At3g23140, At3g23145, At3g23220, At3g23230, At3g23245, At3g23250,
At3g23290, At3g23295, At3g23430, At3g23570, At3g23630, At3g23840,
At3g23860, At3g23955, At3g23960, At3g23970, At3g24060, At3g24130,
At3g24140, At3g24220, At3g24225, At3g24250, At3g24280, At3g24290,
At3g24300, At3g24400, At3g24420, At3g24515, At3g24540, At3g24570,
At3g24580, At3g24590, At3g24610, At3g24640, At3g24650, At3g24690,
At3g24700, At3g24710, At3g24770, At3g24780, At3g25080, At3g25160,
At3g25170, At3g25180, At3g25190, At3g25200, At3g25240, At3g25260,
At3g25265, At3g25270, At3g25280, At3g25430, At3g25460, At3g25490,
At3g25620, At3g25640, At3g25650, At3g25710, At3g25750, At3g25760,
At3g25770, At3g25790, At3g25810, At3g25905, At3g26010, At3g26120,
At3g26140, At3g26150, At3g26190, At3g26200, At3g26270, At3g26290,
At3g26300, At3g26310, At3g26460, At3g26470, At3g26480, At3g26610,
At3g26760, At3g26770, At3g26775, At3g26790, At3g26820, At3g26920,
At3g26930, At3g26935, At3g26940, At3g27095, At3g27150, At3g27250,
At3g27400, At3g27460, At3g27480, At3g27490, At3g27510, At3g27650,
At3g27660, At3g27710, At3g27785, At3g27810, At3g27910, At3g27920,
At3g27970, At3g27980, At3g27990, At3g28007, At3g28020, At3g28220,
At3g28230, At3g28240, At3g28250, At3g28260, At3g28270, At3g28280,
At3g28310, At3g28320, At3g28330, At3g28345, At3g28350, At3g28360,
At3g28390, At3g28410, At3g28415, At3g28500, At3g28520, At3g28570,
At3g28600, At3g28740, At3g28770, At3g28780, At3g28830, At3g28840,
At3g28860, At3g28865, At3g28915, At3g28917, At3g28980, At3g28990,
At3g29032, At3g29040, At3g29050, At3g29060, At3g29110, At3g29156,
At3g29190, At3g29250, At3g29260, At3g29365, At3g29410, At3g29560,
At3g29580, At3g29590, At3g29635, At3g29680, At3g29690, At3g29738,
At3g29739, At3g29830, At3g30110, At3g30210, At3g30260, At3g30280,
At3g30290, At3g30340, At3g30345, At3g30350, At3g30460, At3g30530,
At3g30540, At3g30580, At3g30724, At3g30727, At3g32030, At3g32040,
At3g32047, At3g33000, At3g33002, At3g33004, At3g33076, At3g33103,
At3g33115, At3g33142, At3g42057, At3g42174, At3g42180, At3g42320,
At3g42722, At3g42723, At3g42724, At3g42725, At3g42830, At3g42836,
At3g42837, At3g43110, At3g43120, At3g43190, At3g43200, At3g43250,
At3g43270, At3g43410, At3g43575, At3g43660, At3g43710, At3g43715,
At3g43830, At3g43850, At3g43860, At3g43890, At3g43910, At3g43950,
At3g44080, At3g44090, At3g44096, At3g44100, At3g44220, At3g44260,
At3g44264, At3g44300, At3g44326, At3g44350, At3g44425, At3g44460,
At3g44540, At3g44550, At3g44560, At3g44713, At3g44760, At3g44770,
At3g44810, At3g44910, At3g44920, At3g44930, At3g44970, At3g45070,
At3g45080, At3g45110, At3g45130, At3g45150, At3g45160, At3g45170,
At3g45180, At3g45220, At3g45260, At3g45280, At3g45450, At3g45460,
At3g45470, At3g45480, At3g45500, At3g45510, At3g45530, At3g45540,
At3g45550, At3g45555, At3g45560, At3g45570, At3g45577, At3g45580,
At3g45610, At3g45700, At3g45760, At3g45810, At3g45860, At3g45920,

At3g45940, At3g46070, At3g46080, At3g46090, At3g46186, At3g46190,
At3g46240, At3g46250, At3g46260, At3g46270, At3g46280, At3g46330,
At3g46340, At3g46345, At3g46350, At3g46360, At3g46370, At3g46400,
At3g46420, At3g46460, At3g46570, At3g46710, At3g46810, At3g46910,
At3g47030, At3g47040, At3g47050, At3g47120, At3g47170, At3g47180,
At3g47190, At3g47200, At3g47210, At3g47220, At3g47290, At3g47380,
At3g47400, At3g47480, At3g47540, At3g47570, At3g47600, At3g47620,
At3g47710, At3g47740, At3g47750, At3g47760, At3g47770, At3g47980,
At3g48185, At3g48230, At3g48231, At3g48270, At3g48275, At3g48280,
At3g48300, At3g48310, At3g48340, At3g48510, At3g48520, At3g48550,
At3g48640, At3g48720, At3g48740, At3g48830, At3g48850, At3g48940,
At3g49010, At3g49020, At3g49110, At3g49120, At3g49130, At3g49190,
At3g49200, At3g49210, At3g49270, At3g49280, At3g49305, At3g49330,
At3g49340, At3g49440, At3g49450, At3g49460, At3g49470, At3g49580,
At3g49610, At3g49620, At3g49690, At3g49700, At3g49710, At3g49765,
At3g49770, At3g49780, At3g49790, At3g49950, At3g49960, At3g50120,
At3g50130, At3g50140, At3g50150, At3g50160, At3g50170, At3g50180,
At3g50220, At3g50260, At3g50330, At3g50340, At3g50373, At3g50376,
At3g50390, At3g50450, At3g50470, At3g50500, At3g50505, At3g50510,
At3g50570, At3g50580, At3g50710, At3g50720, At3g50730, At3g50740,
At3g50760, At3g50770, At3g50870, At3g50890, At3g50990, At3g51020,
At3g51060, At3g51070, At3g51190, At3g51200, At3g51420, At3g51560,
At3g51680, At3g51690, At3g51700, At3g51810, At3g51860, At3g51895,
At3g52000, At3g52010, At3g52020, At3g52450, At3g52490, At3g52510,
At3g52527, At3g52550, At3g52560, At3g52610, At3g52670, At3g52680,
At3g52690, At3g52700, At3g52780, At3g52790, At3g52810, At3g52820,
At3g52970, At3g53040, At3g53050, At3g53060, At3g53070, At3g53080,
At3g53090, At3g53232, At3g53280, At3g53290, At3g53300, At3g53330,
At3g53640, At3g53720, At3g53790, At3g53820, At3g53960, At3g53965,
At3g53970, At3g54040, At3g54340, At3g54400, At3g54450, At3g54600,
At3g54700, At3g54770, At3g54940, At3g55100, At3g55110, At3g55130,
At3g55180, At3g55240, At3g55370, At3g55500, At3g55570, At3g55580,
At3g55590, At3g55646, At3g55677, At3g55680, At3g55700, At3g55940,
At3g55970, At3g56040, At3g56060, At3g56220, At3g56230, At3g56275,
At3g56277, At3g56350, At3g56610, At3g56620, At3g56700, At3g56770,
At3g56780, At3g57130, At3g57140, At3g57160, At3g57240, At3g57260,
At3g57460, At3g57510, At3g57620, At3g57670, At3g57950, At3g58020,
At3g58060, At3g58070, At3g58080, At3g58190, At3g58200, At3g58210,
At3g58250, At3g58260, At3g58270, At3g58310, At3g58320, At3g58330,
At3g58340, At3g58350, At3g58360, At3g58370, At3g58390, At3g58440,
At3g58780, At3g58870, At3g58880, At3g58890, At3g58960, At3g58980,
At3g58990, At3g59010, At3g59070, At3g59120, At3g59170, At3g59230,
At3g59240, At3g59440, At3g59460, At3g59590, At3g59600, At3g59610,
At3g59680, At3g59710, At3g59850, At3g59930, At3g60010, At3g60090,
At3g60120, At3g60140, At3g60150, At3g60470, At3g60490, At3g60520,
At3g60560, At3g60570, At3g60650, At3g60670, At3g60700, At3g60710,

At3g60720, At3g60730, At3g60780, At3g60790, At3g60870, At3g60900,
At3g61040, At3g61120, At3g61150, At3g61270, At3g61280, At3g61290,
At3g61340, At3g61350, At3g61510, At3g61700, At3g61720, At3g61820,
At3g61850, At3g61910, At3g61920, At3g61930, At3g61970, At3g62100,
At3g62170, At3g62180, At3g62210, At3g62220, At3g62230, At3g62340,
At3g62350, At3g62380, At3g62440, At3g62455, At3g62460, At3g62610,
At3g62670, At3g62680, At3g62690, At3g62710, At3g62725, At3g62730,
At3g62820, At3g62850, At3g62860, At3g62950, At3g62960, At3g62990,
At3g63110, At3g63160, At3g63170, At4g00026, At4g00030, At4g00070,
At4g00080, At4g00085, At4g00120, At4g00130, At4g00150, At4g00160,
At4g00170, At4g00180, At4g00190, At4g00210, At4g00220, At4g00225,
At4g00250, At4g00260, At4g00300, At4g00310, At4g00315, At4g00320,
At4g00360, At4g00390, At4g00400, At4g00416, At4g00480, At4g00500,
At4g00510, At4g00540, At4g00580, At4g00610, At4g00680, At4g00730,
At4g00870, At4g00880, At4g00885, At4g00920, At4g00940, At4g01140,
At4g01180, At4g01350, At4g01355, At4g01380, At4g01390, At4g01420,
At4g01430, At4g01440, At4g01450, At4g01455, At4g01460, At4g01500,
At4g01520, At4g01525, At4g01530, At4g01720, At4g01890, At4g01920,
At4g01925, At4g01930, At4g01950, At4g01975, At4g01985, At4g02160,
At4g02180, At4g02190, At4g02250, At4g02300, At4g02310, At4g02320,
At4g02670, At4g02690, At4g02700, At4g02733, At4g02780, At4g02810,
At4g02870, At4g03040, At4g03050, At4g03060, At4g03170, At4g03175,
At4g03300, At4g03450, At4g03540, At4g03565, At4g03570, At4g03610,
At4g03620, At4g03630, At4g04120, At4g04370, At4g04375, At4g04450,
At4g04500, At4g04510, At4g04640, At4g04800, At4g04885, At4g04890,
At4g04900, At4g04925, At4g04930, At4g04990, At4g05100, At4g05230,
At4g05240, At4g05260, At4g05270, At4g05430, At4g05470, At4g05490,
At4g05500, At4g06511, At4g06534, At4g08150, At4g08290, At4g08300,
At4g08360, At4g08380, At4g08450, At4g08560, At4g08570, At4g08620,
At4g08670, At4g08730, At4g08740, At4g08750, At4g08770, At4g08867,
At4g08910, At4g08990, At4g09110, At4g09120, At4g09140, At4g09143,
At4g09146, At4g09190, At4g09440, At4g09450, At4g09590, At4g09600,
At4g09610, At4g09644, At4g09647, At4g09690, At4g09740, At4g09745,
At4g09780, At4g09790, At4g09820, At4g09870, At4g09950, At4g09960,
At4g09965, At4g10010, At4g10160, At4g10310, At4g10350, At4g10370,
At4g10380, At4g10490, At4g10595, At4g10660, At4g10670, At4g10680,
At4g10690, At4g10695, At4g10700, At4g10740, At4g10750, At4g10850,
At4g10865, At4g11020, At4g11030, At4g11040, At4g11070, At4g11170,
At4g11180, At4g11190, At4g11200, At4g11210, At4g11290, At4g11310,
At4g11320, At4g11340, At4g11580, At4g11590, At4g11610, At4g11650,
At4g11720, At4g11730, At4g11745, At4g11750, At4g11760, At4g11780,
At4g11880, At4g11930, At4g12050, At4g12100, At4g12150, At4g12160,
At4g12170, At4g12180, At4g12190, At4g12350, At4g12360, At4g12370,
At4g12380, At4g12470, At4g12480, At4g12490, At4g12500, At4g12545,
At4g12550, At4g12810, At4g12820, At4g12890, At4g12920, At4g12950,
At4g12960, At4g12990, At4g13080, At4g13090, At4g13095, At4g13130,

At4g13190, At4g13195, At4g13210, At4g13235, At4g13260, At4g13261, At4g13262, At4g13263, At4g13266, At4g13280, At4g13290, At4g13300, At4g13310, At4g13320, At4g13330, At4g13400, At4g13410, At4g13420, At4g13450, At4g13470, At4g13490, At4g13560, At4g13600, At4g13610, At4g13620, At4g13700, At4g13770, At4g13840, At4g13955, At4g13960, At4g13965, At4g13985, At4g14060, At4g14140, At4g14260, At4g14315, At4g14370, At4g14440, At4g14450, At4g14465, At4g14550, At4g14560, At4g14610, At4g14630, At4g14690, At4g14723, At4g14730, At4g14810, At4g14815, At4g14905, At4g14930, At4g14940, At4g14980, At4g15053, At4g15100, At4g15120, At4g15150, At4g15160, At4g15210, At4g15270, At4g15280, At4g15290, At4g15300, At4g15310, At4g15320, At4g15330, At4g15340, At4g15350, At4g15360, At4g15370, At4g15380, At4g15390, At4g15393, At4g15396, At4g15400, At4g15417, At4g15440, At4g15490, At4g15590, At4g15620, At4g15650, At4g15660, At4g15670, At4g15680, At4g15690, At4g15700, At4g15710, At4g15715, At4g15733, At4g15740, At4g15750, At4g15755, At4g15870, At4g15960, At4g15970, At4g15975, At4g15980, At4g15990, At4g16030, At4g16040, At4g16045, At4g16162, At4g16220, At4g16230, At4g16240, At4g16265, At4g16444, At4g16447, At4g16450, At4g16460, At4g16550, At4g16560, At4g16590, At4g16600, At4g16730, At4g16740, At4g16745, At4g16810, At4g16860, At4g16950, At4g16990, At4g17060, At4g17580, At4g17585, At4g17690, At4g17695, At4g17700, At4g17710, At4g17780, At4g17785, At4g17788, At4g17800, At4g17810, At4g17850, At4g17860, At4g17970, At4g17990, At4g18050, At4g18170, At4g18250, At4g18310, At4g18320, At4g18330, At4g18335, At4g18350, At4g18390, At4g18425, At4g18510, At4g18550, At4g18610, At4g18620, At4g18660, At4g18670, At4g18680, At4g18690, At4g18720, At4g18900, At4g18920, At4g18940, At4g18960, At4g19030, At4g19035, At4g19250, At4g19430, At4g19470, At4g19515, At4g19520, At4g19530, At4g19630, At4g19680, At4g19690, At4g19720, At4g19730, At4g19740, At4g19750, At4g19760, At4g19770, At4g19800, At4g19810, At4g19820, At4g19840, At4g19910, At4g19920, At4g19925, At4g19930, At4g19940, At4g19970, At4g19980, At4g20000, At4g20050, At4g20190, At4g20200, At4g20210, At4g20230, At4g20235, At4g20240, At4g20370, At4g20700, At4g20725, At4g20800, At4g20850, At4g20920, At4g20970, At4g20990, At4g21000, At4g21010, At4g21020, At4g21030, At4g21040, At4g21050, At4g21200, At4g21210, At4g21240, At4g21340, At4g21363, At4g21370, At4g21380, At4g21440, At4g21690, At4g21750, At4g21840, At4g21900, At4g21910, At4g21920, At4g21940, At4g21950, At4g21960, At4g21970, At4g22030, At4g22040, At4g22050, At4g22060, At4g22065, At4g22070, At4g22080, At4g22090, At4g22100, At4g22105, At4g22110, At4g22115, At4g22160, At4g22165, At4g22180, At4g22214, At4g22230, At4g22390, At4g22400, At4g22410, At4g22430, At4g22440, At4g22460, At4g22470, At4g22480, At4g22490, At4g22500, At4g22510, At4g22520, At4g22610, At4g22630, At4g22650, At4g22660, At4g22666, At4g22680, At4g22800, At4g22810, At4g22950, At4g23090, At4g23110, At4g23120, At4g23350, At4g23360, At4g23370, At4g23380, At4g23390, At4g23410, At4g23496,

At4g23520, At4g23550, At4g23560, At4g23580, At4g23590, At4g23600, At4g23670, At4g23680, At4g23713, At4g23760, At4g23840, At4g23870, At4g23990, At4g24000, At4g24010, At4g24050, At4g24060, At4g24070, At4g24130, At4g24140, At4g24180, At4g24310, At4g24540, At4g24640, At4g24650, At4g24660, At4g24980, At4g25010, At4g25020, At4g25040, At4g25090, At4g25130, At4g25140, At4g25190, At4g25210, At4g25250, At4g25400, At4g25410, At4g25430, At4g25434, At4g25435, At4g25780, At4g25790, At4g25820, At4g25850, At4g25920, At4g25930, At4g25950, At4g26050, At4g26150, At4g26200, At4g26250, At4g26260, At4g26280, At4g26330, At4g26340, At4g26350, At4g26380, At4g26420, At4g26480, At4g26490, At4g26820, At4g26870, At4g26880, At4g26920, At4g27140, At4g27150, At4g27160, At4g27170, At4g27250, At4g27330, At4g27420, At4g27590, At4g27810, At4g27840, At4g28040, At4g28088, At4g28090, At4g28100, At4g28110, At4g28340, At4g28365, At4g28395, At4g28410, At4g28420, At4g28500, At4g28510, At4g28520, At4g28530, At4g28640, At4g28650, At4g28680, At4g28780, At4g28840, At4g28850, At4g28860, At4g28920, At4g28940, At4g29020, At4g29030, At4g29100, At4g29110, At4g29150, At4g29230, At4g29280, At4g29370, At4g29570, At4g29580, At4g29600, At4g29610, At4g29620, At4g29630, At4g29640, At4g29650, At4g29970, At4g30030, At4g30040, At4g30050, At4g30120, At4g30140, At4g30170, At4g30230, At4g30410, At4g30440, At4g30450, At4g30460, At4g30540, At4g30590, At4g30770, At4g30980, At4g31250, At4g31260, At4g31330, At4g31380, At4g31390, At4g31590, At4g31610, At4g31710, At4g31730, At4g31830, At4g31877, At4g31900, At4g31910, At4g31940, At4g31950, At4g31970, At4g31980, At4g32040, At4g32090, At4g32220, At4g32230, At4g32280, At4g32300, At4g32320, At4g32370, At4g32375, At4g32380, At4g32490, At4g32500, At4g32540, At4g32760, At4g32780, At4g32810, At4g32860, At4g32880, At4g32890, At4g33220, At4g33230, At4g33290, At4g33300, At4g33330, At4g33420, At4g33450, At4g33710, At4g33720, At4g33730, At4g33790, At4g33800, At4g33810, At4g33830, At4g33840, At4g33850, At4g33860, At4g33880, At4g33900, At4g34060, At4g34170, At4g34250, At4g34320, At4g34415, At4g34420, At4g34520, At4g34580, At4g34760, At4g34980, At4g35030, At4g35080, At4g35120, At4g35150, At4g35160, At4g35165, At4g35170, At4g35180, At4g35360, At4g35370, At4g35380, At4g35390, At4g35640, At4g35650, At4g35670, At4g35690, At4g35700, At4g35710, At4g35720, At4g35820, At4g36160, At4g36170, At4g36220, At4g36250, At4g36260, At4g36280, At4g36350, At4g36440, At4g36560, At4g36570, At4g36610, At4g36620, At4g36710, At4g36740, At4g36870, At4g37050, At4g37060, At4g37070, At4g37390, At4g37400, At4g37410, At4g37650, At4g37710, At4g37720, At4g37730, At4g37750, At4g37770, At4g37780, At4g37850, At4g37860, At4g37940, At4g37950, At4g37970, At4g37980, At4g37990, At4g38000, At4g38030, At4g38190, At4g38220, At4g38340, At4g38420, At4g38770, At4g38920, At4g38980, At4g38990, At4g39000, At4g39010, At4g39290, At4g39360, At4g39410, At4g39480, At4g39590, At4g39610, At4g39615, At4g39650, At4g39720, At4g39740, At4g39745, At4g39750, At4g39753, At4g39756,

At4g39760, At4g39770, At4g39940, At4g39950, At4g40040, At4g40042,
At5g01050, At5g01070, At5g01120, At5g01150, At5g01190, At5g01280,
At5g01310, At5g01330, At5g01370, At5g01380, At5g01480, At5g01490,
At5g01620, At5g01680, At5g01690, At5g01740, At5g01747, At5g01880,
At5g01900, At5g02000, At5g02030, At5g02110, At5g02350, At5g02360,
At5g02460, At5g02510, At5g02700, At5g02920, At5g02980, At5g02990,
At5g03000, At5g03150, At5g03220, At5g03360, At5g03480, At5g03500,
At5g03590, At5g03600, At5g03680, At5g03790, At5g03795, At5g03800,
At5g03810, At5g03820, At5g03840, At5g03890, At5g03980, At5g04120,
At5g04200, At5g04230, At5g04275, At5g04310, At5g04347, At5g04350,
At5g04360, At5g04370, At5g04380, At5g04680, At5g04690, At5g04700,
At5g04950, At5g04960, At5g04970, At5g05020, At5g05040, At5g05120,
At5g05150, At5g05260, At5g05290, At5g05300, At5g05340, At5g05770,
At5g05790, At5g05965, At5g06040, At5g06070, At5g06080, At5g06090,
At5g06150, At5g06170, At5g06250, At5g06330, At5g06380, At5g06500,
At5g06570, At5g06630, At5g06640, At5g06650, At5g06710, At5g06720,
At5g06839, At5g07000, At5g07010, At5g07030, At5g07040, At5g07050,
At5g07060, At5g07080, At5g07160, At5g07190, At5g07200, At5g07210,
At5g07225, At5g07260, At5g07310, At5g07330, At5g07410, At5g07420,
At5g07430, At5g07480, At5g07490, At5g07510, At5g07520, At5g07530,
At5g07540, At5g07550, At5g07560, At5g07590, At5g07600, At5g07690,
At5g07700, At5g07780, At5g07930, At5g07990, At5g08020, At5g08070,
At5g08141, At5g08150, At5g08460, At5g08490, At5g08640, At5g09210,
At5g09320, At5g09340, At5g09360, At5g09470, At5g09480, At5g09490,
At5g09500, At5g09560, At5g09570, At5g09720, At5g09750, At5g09890,
At5g09970, At5g10120, At5g10140, At5g10180, At5g10230, At5g10280,
At5g10420, At5g10510, At5g10580, At5g10590, At5g10930, At5g11050,
At5g11060, At5g11120, At5g11180, At5g11190, At5g11210, At5g11220,
At5g11320, At5g11530, At5g11540, At5g11550, At5g11570, At5g11620,
At5g11850, At5g11930, At5g11940, At5g11977, At5g12000, At5g12070,
At5g12270, At5g12330, At5g12340, At5g12420, At5g12870, At5g13080,
At5g13130, At5g13170, At5g13220, At5g13240, At5g13250, At5g13350,
At5g13380, At5g13780, At5g13825, At5g13880, At5g13910, At5g13930,
At5g14010, At5g14180, At5g14300, At5g14340, At5g14400, At5g14570,
At5g14670, At5g14690, At5g14740, At5g14810, At5g14860, At5g14920,
At5g14940, At5g15030, At5g15040, At5g15100, At5g15110, At5g15130,
At5g15140, At5g15150, At5g15160, At5g15380, At5g15410, At5g15500,
At5g15660, At5g15670, At5g15725, At5g15800, At5g15830, At5g16080,
At5g16090, At5g16285, At5g16330, At5g16460, At5g16500, At5g16530,
At5g16560, At5g16700, At5g16710, At5g16740, At5g16770, At5g16920,
At5g17030, At5g17050, At5g17070, At5g17090, At5g17100, At5g17110,
At5g17120, At5g17200, At5g17260, At5g17320, At5g17340, At5g17430,
At5g17490, At5g17500, At5g17700, At5g17725, At5g17730, At5g17740,
At5g17750, At5g17770, At5g17800, At5g17810, At5g17820, At5g17950,
At5g17960, At5g17970, At5g17980, At5g18200, At5g18270, At5g18300,
At5g18350, At5g18460, At5g18560, At5g18910, At5g18930, At5g18990,

At5g19040, At5g19097, At5g19100, At5g19110, At5g19270, At5g19470, At5g19473, At5g19530, At5g19640, At5g19700, At5g19710, At5g19720, At5g19730, At5g19810, At5g19880, At5g19890, At5g20100, At5g20170, At5g20230, At5g20240, At5g20270, At5g20330, At5g20340, At5g20400, At5g20560, At5g20620, At5g20630, At5g20690, At5g20740, At5g20790, At5g20820, At5g20860, At5g21130, At5g21150, At5g21950, At5g21960, At5g22070, At5g22150, At5g22160, At5g22170, At5g22180, At5g22190, At5g22200, At5g22260, At5g22300, At5g22390, At5g22420, At5g22490, At5g22500, At5g22550, At5g22560, At5g22570, At5g22580, At5g22600, At5g22610, At5g22791, At5g22860, At5g22870, At5g22890, At5g22900, At5g22910, At5g22960, At5g22970, At5g22980, At5g23000, At5g23010, At5g23020, At5g23030, At5g23035, At5g23170, At5g23220, At5g23230, At5g23235, At5g23260, At5g23360, At5g23370, At5g23380, At5g23470, At5g23650, At5g23660, At5g23730, At5g23790, At5g23810, At5g23940, At5g23950, At5g23955, At5g23960, At5g23980, At5g23990, At5g24040, At5g24050, At5g24060, At5g24070, At5g24090, At5g24130, At5g24140, At5g24150, At5g24160, At5g24180, At5g24220, At5g24420, At5g24540, At5g24550, At5g24560, At5g24580, At5g24600, At5g24770, At5g24780, At5g24790, At5g24820, At5g24860, At5g24880, At5g24910, At5g24940, At5g24950, At5g25045, At5g25110, At5g25160, At5g25230, At5g25290, At5g25300, At5g25305, At5g25320, At5g25330, At5g25340, At5g25370, At5g25390, At5g25410, At5g25415, At5g25420, At5g25425, At5g25490, At5g25550, At5g25610, At5g25620, At5g25810, At5g25830, At5g25840, At5g25850, At5g25860, At5g25870, At5g25950, At5g25955, At5g25960, At5g25970, At5g25980, At5g25990, At5g26000, At5g26010, At5g26050, At5g26060, At5g26070, At5g26080, At5g26090, At5g26100, At5g26120, At5g26130, At5g26150, At5g26190, At5g26310, At5g26320, At5g26330, At5g26340, At5g26617, At5g26620, At5g26660, At5g26810, At5g27037, At5g27310, At5g27530, At5g27690, At5g27870, At5g27900, At5g28085, At5g28160, At5g28180, At5g28190, At5g28210, At5g28237, At5g28300, At5g28470, At5g28490, At5g28510, At5g28520, At5g28635, At5g28640, At5g28680, At5g28690, At5g28870, At5g30500, At5g32520, At5g32825, At5g33075, At5g33175, At5g33210, At5g33340, At5g33350, At5g33355, At5g33428, At5g35073, At5g35110, At5g35550, At5g35580, At5g35660, At5g35760, At5g35770, At5g35777, At5g35870, At5g36110, At5g36140, At5g36150, At5g36200, At5g36293, At5g36870, At5g36910, At5g36930, At5g36980, At5g37040, At5g37050, At5g37060, At5g37150, At5g37170, At5g37180, At5g37200, At5g37220, At5g37280, At5g37300, At5g37400, At5g37420, At5g37470, At5g37478, At5g37650, At5g37660, At5g37690, At5g37730, At5g37800, At5g37810, At5g37820, At5g37840, At5g37860, At5g37870, At5g37910, At5g37920, At5g37940, At5g37950, At5g37960, At5g37970, At5g37980, At5g37990, At5g38000, At5g38010, At5g38020, At5g38030, At5g38080, At5g38090, At5g38100, At5g38120, At5g38160, At5g38195, At5g38230, At5g38240, At5g38340, At5g38344, At5g38350, At5g38386, At5g38393, At5g38396, At5g38400, At5g38435, At5g38440, At5g38450, At5g38540, At5g38550, At5g38565, At5g38570, At5g38660,

At5g38670, At5g38680, At5g38750, At5g38760, At5g38770, At5g38780,
At5g38790, At5g38800, At5g38810, At5g38910, At5g38940, At5g38960,
At5g38990, At5g39000, At5g39020, At5g39030, At5g39095, At5g39100,
At5g39110, At5g39120, At5g39130, At5g39180, At5g39230, At5g39260,
At5g39270, At5g39280, At5g39290, At5g39300, At5g39310, At5g39410,
At5g39430, At5g39440, At5g39460, At5g39470, At5g39480, At5g39533,
At5g39540, At5g39560, At5g39580, At5g39620, At5g39630, At5g39650,
At5g39700, At5g39720, At5g39770, At5g39860, At5g39870, At5g39880,
At5g39890, At5g39910, At5g39920, At5g39930, At5g39940, At5g40020,
At5g40040, At5g40050, At5g40260, At5g40310, At5g40330, At5g40350,
At5g40430, At5g40560, At5g40620, At5g40630, At5g40750, At5g40790,
At5g40800, At5g41250, At5g41290, At5g41300, At5g41310, At5g41315,
At5g41490, At5g41500, At5g41505, At5g41510, At5g41570, At5g41630,
At5g41640, At5g41663, At5g41820, At5g41905, At5g42200, At5g42260,
At5g42430, At5g42440, At5g42580, At5g42590, At5g42600, At5g42610,
At5g42630, At5g42645, At5g42800, At5g42900, At5g42930, At5g43110,
At5g43120, At5g43180, At5g43290, At5g43340, At5g43350, At5g43360,
At5g43370, At5g43410, At5g43520, At5g43580, At5g43590, At5g43610,
At5g43640, At5g43840, At5g43870, At5g43935, At5g44060, At5g44120,
At5g44140, At5g44160, At5g44170, At5g44220, At5g44345, At5g44400,
At5g44417, At5g44418, At5g44420, At5g44430, At5g44440, At5g44470,
At5g44620, At5g44630, At5g44770, At5g44860, At5g44870, At5g44940,
At5g44950, At5g44960, At5g44970, At5g44973, At5g44980, At5g45000,
At5g45030, At5g45105, At5g45180, At5g45200, At5g45210, At5g45230,
At5g45450, At5g45530, At5g45630, At5g45640, At5g45650, At5g45690,
At5g45730, At5g45830, At5g45860, At5g45870, At5g45875, At5g45880,
At5g45890, At5g45950, At5g45980, At5g45990, At5g46000, At5g46060,
At5g46130, At5g46260, At5g46270, At5g46300, At5g46310, At5g46315,
At5g46350, At5g46360, At5g46370, At5g46490, At5g46540, At5g46610,
At5g46660, At5g46670, At5g46690, At5g46810, At5g46820, At5g46830,
At5g46873, At5g46874, At5g46875, At5g46877, At5g46880, At5g46890,
At5g46900, At5g46950, At5g46980, At5g46990, At5g47000, At5g47120,
At5g47130, At5g47500, At5g47560, At5g47600, At5g47920, At5g47980,
At5g47990, At5g48000, At5g48010, At5g48050, At5g48060, At5g48100,
At5g48110, At5g48130, At5g48140, At5g48200, At5g48205, At5g48270,
At5g48280, At5g48375, At5g48420, At5g48430, At5g48550, At5g48580,
At5g48980, At5g49050, At5g49090, At5g49130, At5g49140, At5g49180,
At5g49240, At5g49250, At5g49260, At5g49270, At5g49310, At5g49320,
At5g49330, At5g49420, At5g49490, At5g49520, At5g49620, At5g49630,
At5g49660, At5g49750, At5g49770, At5g49780, At5g49790, At5g49800,
At5g49850, At5g49860, At5g49870, At5g50050, At5g50080, At5g50090,
At5g50115, At5g50120, At5g50130, At5g50190, At5g50200, At5g50220,
At5g50300, At5g50360, At5g50470, At5g50480, At5g50490, At5g50500,
At5g50750, At5g50760, At5g50770, At5g50790, At5g50800, At5g50820,
At5g50830, At5g50880, At5g50910, At5g50950, At5g51000, At5g51060,
At5g51310, At5g51360, At5g51370, At5g51470, At5g51480, At5g51490,

At5g51500, At5g51520, At5g51590, At5g51760, At5g51780, At5g51790, At5g51795, At5g51800, At5g51860, At5g51870, At5g51930, At5g51950, At5g52055, At5g52130, At5g52140, At5g52145, At5g52150, At5g52160, At5g52240, At5g52320, At5g52330, At5g52350, At5g52355, At5g52360, At5g52390, At5g52400, At5g52460, At5g52470, At5g52500, At5g52570, At5g52610, At5g52620, At5g52670, At5g52680, At5g52690, At5g52700, At5g52710, At5g52720, At5g52730, At5g52900, At5g52980, At5g53190, At5g53210, At5g53260, At5g53270, At5g53380, At5g53390, At5g53487, At5g53510, At5g53600, At5g53610, At5g53640, At5g53730, At5g53780, At5g53790, At5g53810, At5g53820, At5g53830, At5g53950, At5g53960, At5g54000, At5g54020, At5g54040, At5g54062, At5g54230, At5g54240, At5g54250, At5g54320, At5g54330, At5g54340, At5g54350, At5g54360, At5g54365, At5g54370, At5g54480, At5g54560, At5g54610, At5g54620, At5g54660, At5g54700, At5g54710, At5g54740, At5g54745, At5g54790, At5g54820, At5g55045, At5g55050, At5g55080, At5g55110, At5g55131, At5g55132, At5g55180, At5g55240, At5g55250, At5g55560, At5g55680, At5g55720, At5g55750, At5g55770, At5g55780, At5g55800, At5g55875, At5g55890, At5g55893, At5g55896, At5g56060, At5g56070, At5g56075, At5g56080, At5g56200, At5g56240, At5g56250, At5g56260, At5g56300, At5g56325, At5g56330, At5g56368, At5g56369, At5g56370, At5g56380, At5g56390, At5g56400, At5g56410, At5g56430, At5g56440, At5g56480, At5g56560, At5g56620, At5g56640, At5g56690, At5g56700, At5g56840, At5g56860, At5g56910, At5g56920, At5g56960, At5g56970, At5g56990, At5g57090, At5g57130, At5g57380, At5g57390, At5g57420, At5g57520, At5g57620, At5g57625, At5g57670, At5g57720, At5g57750, At5g57780, At5g57785, At5g58390, At5g58400, At5g58460, At5g58850, At5g58860, At5g58890, At5g58910, At5g59030, At5g59040, At5g59090, At5g59100, At5g59105, At5g59120, At5g59220, At5g59305, At5g59310, At5g59320, At5g59330, At5g59340, At5g59360, At5g59390, At5g59510, At5g59520, At5g59820, At5g59930, At5g60010, At5g60110, At5g60200, At5g60250, At5g60340, At5g60470, At5g60480, At5g60490, At5g60610, At5g60615, At5g60660, At5g60720, At5g60760, At5g60770, At5g60810, At5g60890, At5g60910, At5g60970, At5g61070, At5g61080, At5g61090, At5g61100, At5g61110, At5g61120, At5g61160, At5g61420, At5g61480, At5g61605, At5g61610, At5g61620, At5g61630, At5g61680, At5g61690, At5g61700, At5g61710, At5g61720, At5g61730, At5g61740, At5g61850, At5g61890, At5g62165, At5g62320, At5g62330, At5g62340, At5g62470, At5g62510, At5g62660, At5g62800, At5g62830, At5g62850, At5g62940, At5g62970, At5g63090, At5g63170, At5g63180, At5g63230, At5g63340, At5g63350, At5g63450, At5g63560, At5g63580, At5g63590, At5g63595, At5g63600, At5g63620, At5g63715, At5g63740, At5g63750, At5g63930, At5g64100, At5g64110, At5g64120, At5g64530, At5g64550, At5g64700, At5g64770, At5g64780, At5g64790, At5g64810, At5g64870, At5g64905, At5g65070, At5g65080, At5g65100, At5g65170, At5g65230, At5g65280, At5g65320, At5g65330, At5g65340, At5g65380, At5g65510, At5g65590, At5g65690, At5g65730, At5g65790, At5g65800, At5g65850, At5g65920, At5g66120,

At5g66130, At5g66220, At5g66230, At5g66260, At5g66280, At5g66300, At5g66350, At5g66380, At5g66390, At5g66400, At5g66430, At5g66690, At5g66700, At5g66815, At5g66817, At5g66870, At5g66890, At5g66970, At5g66980, At5g66990, At5g67000, At5g67400, At5g67411
