# Supplementary material for: Whole-Genome Analysis of Histone H3 Lysine 27 Trimethylation in Arabidopsis
Source: PLoS Biol. 2007 Apr 17;5(5):e129. doi: 10.1371/journal.pbio.0050129 (PMC1852588; doi:10.1371/journal.pbio.0050129)
Supplement: Supplementary file 2 [file pbio.0050129.sd002.pdf]

Supplemental Data 2. List of *Arabidopsis* genes with LND regions in their promoters (LND promoter genes).

At1g01020, At1g01060, At1g01100, At1g01140, At1g01210, At1g01225, At1g01270, At1g01280, At1g01290, At1g01320, At1g01420, At1g01430, At1g01440, At1g01470, At1g01480, At1g01490, At1g01500, At1g01540, At1g01550, At1g01560, At1g01570, At1g01590, At1g01620, At1g01630, At1g01650, At1g01680, At1g01690, At1g01710, At1g01720, At1g01730, At1g01800, At1g01810, At1g01880, At1g02020, At1g02140, At1g02160, At1g02230, At1g02305, At1g02340, At1g02350, At1g02420, At1g02510, At1g02590, At1g02600, At1g02610, At1g02620, At1g02650, At1g02670, At1g02740, At1g02750, At1g02816, At1g02850, At1g02880, At1g02970, At1g02990, At1g03010, At1g03030, At1g03040, At1g03050, At1g03080, At1g03160, At1g03180, At1g03200, At1g03220, At1g03230, At1g03250, At1g03260, At1g03270, At1g03290, At1g03300, At1g03310, At1g03350, At1g03360, At1g03430, At1g03440, At1g03510, At1g03515, At1g03560, At1g03570, At1g03580, At1g03600, At1g03610, At1g03630, At1g03670, At1g03680, At1g03687, At1g03743, At1g03746, At1g03750, At1g03760, At1g03780, At1g03860, At1g03870, At1g03930, At1g03970, At1g03980, At1g04080, At1g04150, At1g04160, At1g04190, At1g04200, At1g04260, At1g04270, At1g04340, At1g04350, At1g04390, At1g04440, At1g04445, At1g04520, At1g04530, At1g04570, At1g04635, At1g04640, At1g04690, At1g04730, At1g04810, At1g04820, At1g04970, At1g04990, At1g05000, At1g05010, At1g05080, At1g05090, At1g05100, At1g05140, At1g05180, At1g05310, At1g05320, At1g05340, At1g05385, At1g05430, At1g05805, At1g05840, At1g05850, At1g05860, At1g05910, At1g05950, At1g05960, At1g06040, At1g06070, At1g06140, At1g06190, At1g06220, At1g06230, At1g06290, At1g06390, At1g06400, At1g06420, At1g06470, At1g06480, At1g06490, At1g06530, At1g06570, At1g06590, At1g06610, At1g06680, At1g06700, At1g06740, At1g06790, At1g06850, At1g06860, At1g06980, At1g07000, At1g07010, At1g07040, At1g07080, At1g07100, At1g07110, At1g07130, At1g07160, At1g07170, At1g07250, At1g07270, At1g07280, At1g07290, At1g07310, At1g07320, At1g07330, At1g07390, At1g07420, At1g07430, At1g07510, At1g07580, At1g07630, At1g07660, At1g07795, At1g07870, At1g07890, At1g07910, At1g07920, At1g07930, At1g07980, At1g08125, At1g08130, At1g08180, At1g08210, At1g08220, At1g08300, At1g08380, At1g08390, At1g08450, At1g08510, At1g08530, At1g08570, At1g08580, At1g08610, At1g08650, At1g08790, At1g08830, At1g08920, At1g09010, At1g09070, At1g09080, At1g09210, At1g09230, At1g09250, At1g09270, At1g09280, At1g09320, At1g09330, At1g09340, At1g09415, At1g09570, At1g09645, At1g09710, At1g09830, At1g09860, At1g09870, At1g09970, At1g09980, At1g10040, At1g10050, At1g10090, At1g10150, At1g10180, At1g10200, At1g10210, At1g10340, At1g10420, At1g10440, At1g10450, At1g10470, At1g10490, At1g10500, At1g10510, At1g10570, At1g10590, At1g10600, At1g10670, At1g10700, At1g10840, At1g10910, At1g10940, At1g10950, At1g10960, At1g10980, At1g10990, At1g11000,

At1g11010, At1g11050, At1g11090, At1g11200, At1g11210, At1g11240, At1g11250, At1g11310, At1g11330, At1g11440, At1g11530, At1g11545, At1g11550, At1g11560, At1g11710, At1g11740, At1g11750, At1g11770, At1g11790, At1g11840, At1g11880, At1g11890, At1g11910, At1g11970, At1g12050, At1g12060, At1g12140, At1g12220, At1g12244, At1g12310, At1g12360, At1g12420, At1g12440, At1g12500, At1g12600, At1g12670, At1g12740, At1g12760, At1g12800, At1g12830, At1g12840, At1g12900, At1g12910, At1g12920, At1g12990, At1g13030, At1g13040, At1g13060, At1g13080, At1g13090, At1g13190, At1g13210, At1g13240, At1g13260, At1g13280, At1g13300, At1g13350, At1g13380, At1g13410, At1g13470, At1g13720, At1g13730, At1g13740, At1g13820, At1g13830, At1g13840, At1g13860, At1g13940, At1g14000, At1g14010, At1g14140, At1g14150, At1g14370, At1g14400, At1g14430, At1g14540, At1g14590, At1g14620, At1g14640, At1g14685, At1g14690, At1g14710, At1g14740, At1g14910, At1g14920, At1g14990, At1g15030, At1g15060, At1g15120, At1g15180, At1g15320, At1g15330, At1g15340, At1g15390, At1g15400, At1g15480, At1g15530, At1g15540, At1g15570, At1g15580, At1g15690, At1g15710, At1g15740, At1g15757, At1g15800, At1g15810, At1g15820, At1g15860, At1g15930, At1g15940, At1g15970, At1g15980, At1g16010, At1g16020, At1g16040, At1g16130, At1g16150, At1g16170, At1g16180, At1g16190, At1g16210, At1g16460, At1g16470, At1g16480, At1g16720, At1g16790, At1g16860, At1g16920, At1g16970, At1g17020, At1g17060, At1g17100, At1g17120, At1g17140, At1g17230, At1g17280, At1g17290, At1g17370, At1g17460, At1g17500, At1g17530, At1g17550, At1g17560, At1g17570, At1g17690, At1g17720, At1g17745, At1g17750, At1g17850, At1g17860, At1g17880, At1g17960, At1g17970, At1g18060, At1g18080, At1g18150, At1g18265, At1g18290, At1g18300, At1g18380, At1g18470, At1g18480, At1g18490, At1g18610, At1g18630, At1g18670, At1g18720, At1g18730, At1g18740, At1g18750, At1g18830, At1g18900, At1g18950, At1g18980, At1g19000, At1g19130, At1g19170, At1g19220, At1g19300, At1g19340, At1g19350, At1g19360, At1g19373, At1g19376, At1g19380, At1g19400, At1g19450, At1g19460, At1g19660, At1g19680, At1g19690, At1g19700, At1g19720, At1g19730, At1g19740, At1g19770, At1g19780, At1g19800, At1g19860, At1g19880, At1g19890, At1g19910, At1g19950, At1g20015, At1g20030, At1g20100, At1g20110, At1g20440, At1g20480, At1g20575, At1g20580, At1g20670, At1g20680, At1g20696, At1g20820, At1g20823, At1g20830, At1g20890, At1g21050, At1g21080, At1g21110, At1g21120, At1g21280, At1g21350, At1g21380, At1g21590, At1g21900, At1g21910, At1g21930, At1g22140, At1g22280, At1g22300, At1g22330, At1g22360, At1g22420, At1g22440, At1g22450, At1g22460, At1g22470, At1g22520, At1g22570, At1g22610, At1g22660, At1g22700, At1g22750, At1g22770, At1g22830, At1g22870, At1g22882, At1g22930, At1g22970, At1g22985, At1g23020, At1g23030, At1g23205, At1g23330, At1g23490, At1g23540, At1g23710, At1g23740, At1g23750, At1g23850, At1g23860, At1g23970, At1g24070, At1g24080, At1g24090, At1g24120, At1g24260, At1g24350, At1g24360, At1g24530, At1g24650, At1g24706, At1g25260, At1g25370,

At1g25375, At1g25380, At1g25390, At1g25420, At1g25480, At1g25520, At1g25540, At1g25550, At1g26090, At1g26150, At1g26160, At1g26190, At1g26270, At1g26420, At1g26430, At1g26440, At1g26460, At1g26540, At1g26550, At1g26570, At1g26630, At1g26640, At1g26650, At1g26660, At1g26730, At1g26850, At1g26880, At1g27020, At1g27100, At1g27120, At1g27190, At1g27290, At1g27320, At1g27330, At1g27390, At1g27400, At1g27440, At1g27450, At1g27630, At1g27730, At1g27890, At1g27980, At1g28070, At1g28100, At1g28200, At1g28280, At1g28330, At1g28490, At1g28510, At1g28580, At1g28680, At1g29150, At1g29195, At1g29250, At1g29260, At1g29310, At1g29340, At1g29470, At1g29550, At1g29650, At1g29670, At1g29800, At1g29830, At1g29900, At1g29950, At1g29965, At1g30010, At1g30050, At1g30110, At1g30130, At1g30180, At1g30240, At1g30280, At1g30380, At1g30400, At1g30600, At1g30630, At1g30660, At1g30680, At1g30690, At1g30700, At1g30825, At1g30845, At1g30890, At1g31150, At1g31180, At1g31190, At1g31220, At1g31240, At1g31320, At1g31330, At1g31350, At1g31470, At1g31540, At1g31760, At1g31812, At1g31814, At1g31817, At1g31835, At1g31840, At1g31870, At1g32050, At1g32060, At1g32200, At1g32260, At1g32400, At1g32410, At1g32460, At1g32500, At1g32510, At1g32610, At1g32620, At1g32630, At1g32640, At1g32690, At1g32700, At1g32760, At1g32790, At1g32800, At1g32920, At1g32928, At1g33020, At1g33030, At1g33040, At1g33060, At1g33250, At1g33260, At1g33420, At1g33470, At1g33607, At1g33970, At1g33980, At1g34040, At1g34210, At1g34220, At1g34380, At1g34570, At1g34750, At1g35465, At1g35510, At1g35535, At1g35750, At1g36070, At1g36730, At1g36915, At1g43130, At1g43670, At1g43700, At1g43835, At1g44350, At1g44790, At1g46192, At1g46552, At1g47310, At1g47317, At1g47330, At1g47380, At1g47500, At1g47530, At1g47750, At1g47970, At1g48267, At1g48410, At1g48460, At1g48540, At1g48610, At1g48630, At1g48635, At1g48770, At1g48920, At1g48960, At1g48970, At1g48980, At1g49140, At1g49150, At1g49300, At1g49340, At1g49400, At1g49480, At1g49510, At1g49640, At1g49710, At1g49720, At1g49740, At1g49820, At1g49840, At1g49870, At1g49890, At1g49900, At1g50000, At1g50010, At1g50020, At1g50040, At1g50055, At1g50280, At1g50410, At1g50440, At1g50480, At1g50640, At1g50660, At1g50760, At1g50910, At1g50920, At1g51070, At1g51100, At1g51370, At1g51405, At1g51550, At1g51760, At1g51890, At1g52150, At1g52250, At1g52280, At1g52310, At1g52370, At1g52510, At1g52520, At1g52620, At1g52720, At1g52780, At1g52860, At1g52870, At1g52890, At1g53040, At1g53050, At1g53180, At1g53190, At1g53280, At1g53345, At1g53380, At1g53470, At1g53530, At1g53580, At1g53600, At1g53670, At1g53710, At1g53720, At1g53750, At1g53840, At1g54050, At1g54060, At1g54090, At1g54100, At1g54110, At1g54140, At1g54150, At1g54270, At1g54360, At1g54520, At1g54670, At1g54680, At1g54690, At1g54700, At1g54770, At1g54780, At1g54990, At1g55010, At1g55080, At1g55090, At1g55120, At1g55160, At1g55190, At1g55340, At1g55350, At1g55365, At1g55450, At1g55460, At1g55490, At1g55500, At1g55510, At1g55550, At1g55670, At1g55690, At1g55810, At1g55820, At1g55830,

At1g55900, At1g55910, At1g55930, At1g56010, At1g56020, At1g56070, At1g56140, At1g56145, At1g56220, At1g56330, At1g56345, At1g56350, At1g56400, At1g56450, At1g56560, At1g56570, At1g56590, At1g57720, At1g57790, At1g57906, At1g58060, At1g58180, At1g58200, At1g58210, At1g58280, At1g58290, At1g58360, At1g58420, At1g59550, At1g59820, At1g59840, At1g59870, At1g59900, At1g59910, At1g59980, At1g60300, At1g60420, At1g60430, At1g60440, At1g60650, At1g60660, At1g60670, At1g60800, At1g60900, At1g60970, At1g61040, At1g61110, At1g61210, At1g61350, At1g61400, At1g61540, At1g61560, At1g61570, At1g61667, At1g61670, At1g61688, At1g61780, At1g61790, At1g61810, At1g61870, At1g61880, At1g61890, At1g61910, At1g62200, At1g62320, At1g62350, At1g62370, At1g62380, At1g62420, At1g62520, At1g62740, At1g62800, At1g62850, At1g62855, At1g62870, At1g62880, At1g62960, At1g63055, At1g63080, At1g63130, At1g63290, At1g63300, At1g63430, At1g63500, At1g63670, At1g63690, At1g63780, At1g63790, At1g63830, At1g63840, At1g63850, At1g63960, At1g64040, At1g64050, At1g64100, At1g64120, At1g64130, At1g64140, At1g64170, At1g64180, At1g64185, At1g64190, At1g64200, At1g64300, At1g64420, At1g64430, At1g64470, At1g64490, At1g64610, At1g64650, At1g64990, At1g65000, At1g65030, At1g65060, At1g65370, At1g65410, At1g65420, At1g65440, At1g65560, At1g65790, At1g65850, At1g65950, At1g65980, At1g66150, At1g66160, At1g66190, At1g66250, At1g66410, At1g66580, At1g66600, At1g66740, At1g66890, At1g67100, At1g67180, At1g67190, At1g67195, At1g67210, At1g67240, At1g67350, At1g67370, At1g67480, At1g67540, At1g67550, At1g67580, At1g67775, At1g67830, At1g67880, At1g67890, At1g67920, At1g67930, At1g67940, At1g67960, At1g68020, At1g68130, At1g68190, At1g68440, At1g68520, At1g68550, At1g68620, At1g68640, At1g68660, At1g68680, At1g68690, At1g68710, At1g68720, At1g68840, At1g69010, At1g69060, At1g69120, At1g69200, At1g69260, At1g69270, At1g69310, At1g69340, At1g69570, At1g69620, At1g69640, At1g69680, At1g69750, At1g69890, At1g69960, At1g70000, At1g70050, At1g70100, At1g70190, At1g70200, At1g70210, At1g70230, At1g70250, At1g70290, At1g70330, At1g70350, At1g70360, At1g70450, At1g70480, At1g70520, At1g70570, At1g70580, At1g70590, At1g70620, At1g70660, At1g70700, At1g70790, At1g70920, At1g70950, At1g70985, At1g71015, At1g71040, At1g71050, At1g71080, At1g71090, At1g71350, At1g71370, At1g71420, At1g71430, At1g71490, At1g71500, At1g71520, At1g71697, At1g71710, At1g71730, At1g71740, At1g71770, At1g71780, At1g71790, At1g71840, At1g71850, At1g71860, At1g71890, At1g71900, At1g71980, At1g72010, At1g72020, At1g72030, At1g72050, At1g72160, At1g72190, At1g72310, At1g72320, At1g72330, At1g72420, At1g72430, At1g72440, At1g72450, At1g72510, At1g72520, At1g72630, At1g72680, At1g72690, At1g72700, At1g72730, At1g72750, At1g72770, At1g73020, At1g73030, At1g73150, At1g73160, At1g73170, At1g73175, At1g73230, At1g73350, At1g73390, At1g73445, At1g73450, At1g73480, At1g73670, At1g73680, At1g73730, At1g73760, At1g73770, At1g73830, At1g73860, At1g73880, At1g73890, At1g73900, At1g73910,

At1g73920, At1g73940, At1g73960, At1g74030, At1g74040, At1g74060,
At1g74270, At1g74280, At1g74360, At1g74450, At1g74453, At1g74456,
At1g74470, At1g74640, At1g74650, At1g74710, At1g74780, At1g74840,
At1g74950, At1g74970, At1g75070, At1g75080, At1g75130, At1g75163,
At1g75166, At1g75180, At1g75220, At1g75310, At1g75340, At1g75350,
At1g75360, At1g75380, At1g75400, At1g75420, At1g75440, At1g75460,
At1g75480, At1g75490, At1g75510, At1g75560, At1g75630, At1g75660,
At1g75690, At1g75730, At1g75740, At1g75760, At1g75770, At1g75790,
At1g75820, At1g75840, At1g75850, At1g75870, At1g76010, At1g76030,
At1g76035, At1g76070, At1g76080, At1g76090, At1g76100, At1g76140,
At1g76150, At1g76170, At1g76180, At1g76185, At1g76280, At1g76320,
At1g76330, At1g76380, At1g76390, At1g76490, At1g76550, At1g76570,
At1g76600, At1g76650, At1g76660, At1g76700, At1g76730, At1g76740,
At1g76850, At1g76870, At1g76880, At1g76940, At1g76950, At1g77050,
At1g77080, At1g77122, At1g77290, At1g77405, At1g77410, At1g77420,
At1g77480, At1g77490, At1g77550, At1g77560, At1g77570, At1g77620,
At1g77630, At1g77700, At1g77710, At1g77720, At1g77765, At1g77800,
At1g77850, At1g77930, At1g78040, At1g78050, At1g78070, At1g78100,
At1g78130, At1g78150, At1g78190, At1g78230, At1g78240, At1g78270,
At1g78280, At1g78290, At1g78310, At1g78380, At1g78420, At1g78460,
At1g78480, At1g78510, At1g78530, At1g78550, At1g78590, At1g78620,
At1g78630, At1g78660, At1g78670, At1g78680, At1g78690, At1g78700,
At1g78710, At1g78790, At1g78810, At1g78830, At1g78840, At1g78860,
At1g78890, At1g78900, At1g78990, At1g79040, At1g79120, At1g79150,
At1g79190, At1g79245, At1g79300, At1g79310, At1g79340, At1g79360,
At1g79370, At1g79380, At1g79410, At1g79440, At1g79450, At1g79460,
At1g79490, At1g79510, At1g79520, At1g79540, At1g79630, At1g79650,
At1g79670, At1g79680, At1g79710, At1g79730, At1g79820, At1g79960,
At1g79990, At1g80040, At1g80150, At1g80180, At1g80245, At1g80250,
At1g80350, At1g80360, At1g80380, At1g80410, At1g80440, At1g80450,
At1g80460, At1g80480, At1g80490, At1g80500, At1g80520, At1g80530,
At1g80620, At1g80630, At1g80640, At1g80770, At1g80840, At1g80850,
At1g80870, At1g80920, At1g80930, At1g80950, At2g01060, At2g01070,
At2g01140, At2g01180, At2g01250, At2g01290, At2g01320, At2g01330,
At2g01390, At2g01470, At2g01490, At2g01590, At2g01630, At2g01650,
At2g01720, At2g01730, At2g01755, At2g01760, At2g01870, At2g01910,
At2g02040, At2g02050, At2g02090, At2g02147, At2g02400, At2g02410,
At2g02470, At2g02600, At2g02730, At2g02800, At2g02820, At2g02950,
At2g02970, At2g02980, At2g02990, At2g03020, At2g03120, At2g03140,
At2g03240, At2g03310, At2g03320, At2g03440, At2g03450, At2g03480,
At2g03510, At2g03690, At2g03730, At2g03780, At2g03790, At2g03933,
At2g04030, At2g04100, At2g04280, At2g04690, At2g04865, At2g04880,
At2g05220, At2g05300, At2g05310, At2g05765, At2g05790, At2g06025,
At2g06050, At2g06255, At2g06510, At2g06520, At2g06925, At2g07688,
At2g11520, At2g11890, At2g12550, At2g14045, At2g14110, At2g14440,
At2g14460, At2g14846, At2g14860, At2g14890, At2g15230, At2g15480,

At2g15530, At2g15580, At2g15620, At2g15630, At2g15730, At2g15750,
At2g15790, At2g15830, At2g15900, At2g15960, At2g15970, At2g16070,
At2g16250, At2g16280, At2g16430, At2g16530, At2g16570, At2g16600,
At2g16940, At2g16950, At2g17030, At2g17033, At2g17040, At2g17120,
At2g17200, At2g17260, At2g17370, At2g17390, At2g17520, At2g17525,
At2g17550, At2g17560, At2g17570, At2g17640, At2g17670, At2g17710,
At2g17750, At2g17787, At2g17830, At2g18010, At2g18020, At2g18030,
At2g18040, At2g18220, At2g18240, At2g18250, At2g18280, At2g18290,
At2g18330, At2g18390, At2g18560, At2g18700, At2g18720, At2g18730,
At2g18740, At2g18780, At2g18860, At2g18890, At2g18910, At2g19010,
At2g19250, At2g19390, At2g19480, At2g19490, At2g19580, At2g19720,
At2g19730, At2g19740, At2g19750, At2g19800, At2g19930, At2g19940,
At2g20000, At2g20050, At2g20060, At2g20110, At2g20130, At2g20140,
At2g20180, At2g20190, At2g20270, At2g20280, At2g20310, At2g20320,
At2g20360, At2g20480, At2g20490, At2g20510, At2g20515, At2g20670,
At2g20680, At2g20722, At2g20723, At2g20724, At2g20890, At2g20940,
At2g20950, At2g20980, At2g21300, At2g21320, At2g21410, At2g21430,
At2g21520, At2g21530, At2g21580, At2g21620, At2g21660, At2g21690,
At2g21700, At2g21710, At2g21780, At2g21790, At2g22070, At2g22080,
At2g22090, At2g22100, At2g22110, At2g22120, At2g22140, At2g22220,
At2g22230, At2g22280, At2g22380, At2g22530, At2g22540, At2g22670,
At2g22790, At2g22807, At2g22830, At2g22870, At2g22990, At2g23040,
At2g23080, At2g23090, At2g23093, At2g23120, At2g23130, At2g23150,
At2g23430, At2g23460, At2g23690, At2g23770, At2g23780, At2g23910,
At2g23930, At2g24120, At2g24240, At2g24290, At2g24360, At2g24370,
At2g24380, At2g24395, At2g24560, At2g24590, At2g24790, At2g24960,
At2g25010, At2g25060, At2g25080, At2g25095, At2g25110, At2g25200,
At2g25250, At2g25270, At2g25355, At2g25430, At2g25460, At2g25490,
At2g25610, At2g25620, At2g25630, At2g25640, At2g25660, At2g25680,
At2g25690, At2g25720, At2g25735, At2g25737, At2g25760, At2g25850,
At2g25870, At2g25900, At2g25930, At2g26030, At2g26080, At2g26110,
At2g26270, At2g26300, At2g26320, At2g26340, At2g26550, At2g26560,
At2g26600, At2g26650, At2g26660, At2g26670, At2g26710, At2g26980,
At2g27020, At2g27150, At2g27260, At2g27285, At2g27350, At2g27400,
At2g27460, At2g27500, At2g27505, At2g27600, At2g27820, At2g27850,
At2g27970, At2g28060, At2g28100, At2g28200, At2g28320, At2g28350,
At2g28390, At2g28480, At2g28520, At2g28540, At2g28570, At2g28600,
At2g28760, At2g28770, At2g28830, At2g28840, At2g28890, At2g28910,
At2g28920, At2g28940, At2g28950, At2g29080, At2g29180, At2g29190,
At2g29420, At2g29430, At2g29450, At2g29490, At2g29510, At2g29670,
At2g29690, At2g29700, At2g29760, At2g30000, At2g30010, At2g30040,
At2g30050, At2g30120, At2g30200, At2g30280, At2g30320, At2g30440,
At2g30470, At2g30520, At2g30530, At2g30550, At2g30580, At2g30590,
At2g30600, At2g30700, At2g30720, At2g30880, At2g30950, At2g30960,
At2g31010, At2g31020, At2g31040, At2g31230, At2g31260, At2g31350,
At2g31370, At2g31380, At2g31570, At2g31680, At2g31720, At2g31750,

At2g31800, At2g31820, At2g31840, At2g31850, At2g31870, At2g31880,
At2g31940, At2g31957, At2g31970, At2g32020, At2g32090, At2g32150,
At2g32190, At2g32200, At2g32220, At2g32230, At2g32410, At2g32430,
At2g32520, At2g32560, At2g32700, At2g32890, At2g32900, At2g32950,
At2g32960, At2g33040, At2g33050, At2g33120, At2g33130, At2g33170,
At2g33180, At2g33255, At2g33290, At2g33360, At2g33410, At2g33430,
At2g33450, At2g33470, At2g33530, At2g33590, At2g33610, At2g33700,
At2g33710, At2g33730, At2g33830, At2g33835, At2g33840, At2g33855,
At2g33860, At2g33890, At2g34160, At2g34170, At2g34250, At2g34310,
At2g34340, At2g34380, At2g34390, At2g34400, At2g34580, At2g34610,
At2g34690, At2g34720, At2g34770, At2g34780, At2g34860, At2g35010,
At2g35230, At2g35382, At2g35384, At2g35387, At2g35390, At2g35410,
At2g35450, At2g35460, At2g35605, At2g35610, At2g35630, At2g35690,
At2g35744, At2g35747, At2g35750, At2g35780, At2g35790, At2g35880,
At2g35900, At2g35920, At2g35940, At2g36000, At2g36010, At2g36080,
At2g36130, At2g36140, At2g36145, At2g36220, At2g36250, At2g36270,
At2g36280, At2g36290, At2g36310, At2g36320, At2g36350, At2g36420,
At2g36460, At2g36500, At2g36520, At2g36830, At2g36835, At2g36880,
At2g36900, At2g36950, At2g37025, At2g37035, At2g37060, At2g37110,
At2g37120, At2g37140, At2g37150, At2g37160, At2g37270, At2g37410,
At2g37470, At2g37480, At2g37530, At2g37585, At2g37600, At2g37830,
At2g37860, At2g37960, At2g38000, At2g38070, At2g38120, At2g38160,
At2g38185, At2g38255, At2g38260, At2g38290, At2g38400, At2g38440,
At2g38460, At2g38465, At2g38560, At2g38610, At2g38630, At2g38660,
At2g38700, At2g38800, At2g38820, At2g39000, At2g39050, At2g39190,
At2g39260, At2g39270, At2g39280, At2g39290, At2g39360, At2g39400,
At2g39490, At2g39500, At2g39650, At2g39660, At2g39705, At2g39720,
At2g39725, At2g39750, At2g39760, At2g39805, At2g39810, At2g39870,
At2g39930, At2g39950, At2g39960, At2g40000, At2g40060, At2g40080,
At2g40095, At2g40100, At2g40120, At2g40205, At2g40340, At2g40360,
At2g40410, At2g40480, At2g40540, At2g40550, At2g40570, At2g40650,
At2g40730, At2g40745, At2g40800, At2g40810, At2g40830, At2g40935,
At2g40940, At2g41020, At2g41060, At2g41080, At2g41110, At2g41140,
At2g41180, At2g41230, At2g41290, At2g41350, At2g41420, At2g41430,
At2g41540, At2g41550, At2g41680, At2g41700, At2g41705, At2g41740,
At2g41790, At2g41820, At2g41830, At2g41870, At2g41900, At2g42020,
At2g42170, At2g42230, At2g42240, At2g42260, At2g42270, At2g42280,
At2g42300, At2g42330, At2g42350, At2g42360, At2g42390, At2g42395,
At2g42500, At2g42510, At2g42650, At2g42690, At2g42700, At2g42760,
At2g42770, At2g42780, At2g42880, At2g42950, At2g42955, At2g43010,
At2g43040, At2g43090, At2g43110, At2g43120, At2g43300, At2g43340,
At2g43400, At2g43410, At2g43430, At2g43510, At2g43530, At2g43540,
At2g43570, At2g43640, At2g43650, At2g43680, At2g43710, At2g43720,
At2g43790, At2g43820, At2g43910, At2g43930, At2g44050, At2g44065,
At2g44080, At2g44120, At2g44170, At2g44180, At2g44190, At2g44270,
At2g44350, At2g44450, At2g44480, At2g44490, At2g44500, At2g44660,

At2g44670, At2g44710, At2g44735, At2g44750, At2g44770, At2g44860, At2g44870, At2g44930, At2g44950, At2g44970, At2g45010, At2g45040, At2g45060, At2g45070, At2g45080, At2g45160, At2g45170, At2g45180, At2g45240, At2g45250, At2g45290, At2g45350, At2g45460, At2g45590, At2g45630, At2g45680, At2g45690, At2g45720, At2g45730, At2g45740, At2g45750, At2g45780, At2g45790, At2g45820, At2g45880, At2g45910, At2g45960, At2g46050, At2g46060, At2g46160, At2g46170, At2g46220, At2g46260, At2g46270, At2g46280, At2g46290, At2g46300, At2g46340, At2g46390, At2g46410, At2g46500, At2g46505, At2g46540, At2g46550, At2g46580, At2g46590, At2g46600, At2g46610, At2g46620, At2g46650, At2g46680, At2g46700, At2g46710, At2g46730, At2g46735, At2g46800, At2g46810, At2g46820, At2g46830, At2g46840, At2g46930, At2g47010, At2g47060, At2g47070, At2g47090, At2g47100, At2g47110, At2g47130, At2g47170, At2g47180, At2g47230, At2g47290, At2g47380, At2g47470, At2g47490, At2g47600, At2g47620, At2g47640, At2g47650, At2g47700, At2g47710, At2g47720, At2g47730, At2g47760, At2g47790, At2g47980, At2g48030, At2g48160, At3g01040, At3g01060, At3g01150, At3g01160, At3g01170, At3g01220, At3g01280, At3g01290, At3g01313, At3g01316, At3g01430, At3g01470, At3g01490, At3g01510, At3g01516, At3g01530, At3g01540, At3g01560, At3g01590, At3g01610, At3g01640, At3g01700, At3g01740, At3g01750, At3g01760, At3g01810, At3g01830, At3g01850, At3g01860, At3g02010, At3g02040, At3g02120, At3g02140, At3g02180, At3g02250, At3g02280, At3g02320, At3g02330, At3g02335, At3g02340, At3g02360, At3g02370, At3g02450, At3g02460, At3g02470, At3g02530, At3g02570, At3g02580, At3g02590, At3g02600, At3g02700, At3g02710, At3g02800, At3g02830, At3g02840, At3g02860, At3g02880, At3g02910, At3g02920, At3g03010, At3g03020, At3g03050, At3g03110, At3g03150, At3g03160, At3g03170, At3g03310, At3g03320, At3g03380, At3g03550, At3g03560, At3g03570, At3g03710, At3g03740, At3g03773, At3g03790, At3g03855, At3g03870, At3g03920, At3g03960, At3g03970, At3g03990, At3g04040, At3g04060, At3g04110, At3g04120, At3g04130, At3g04260, At3g04445, At3g04550, At3g04605, At3g04620, At3g04640, At3g04650, At3g04680, At3g04717, At3g04730, At3g04820, At3g04830, At3g04840, At3g04930, At3g04940, At3g04960, At3g05070, At3g05090, At3g05100, At3g05110, At3g05120, At3g05210, At3g05220, At3g05230, At3g05340, At3g05370, At3g05420, At3g05430, At3g05500, At3g05520, At3g05530, At3g05550, At3g05580, At3g05630, At3g05650, At3g05670, At3g05690, At3g05700, At3g05710, At3g05830, At3g05835, At3g05840, At3g05910, At3g06010, At3g06080, At3g06105, At3g06110, At3g06130, At3g06280, At3g06300, At3g06350, At3g06370, At3g06380, At3g06410, At3g06430, At3g06450, At3g06455, At3g06483, At3g06500, At3g06510, At3g06520, At3g06530, At3g06550, At3g06670, At3g06730, At3g06750, At3g06810, At3g06840, At3g06895, At3g07020, At3g07025, At3g07040, At3g07050, At3g07110, At3g07190, At3g07260, At3g07310, At3g07350, At3g07390, At3g07400, At3g07440, At3g07500, At3g07525, At3g07530, At3g07620, At3g07630, At3g07670, At3g07680, At3g07720, At3g07780, At3g07790,

At3g07800, At3g07850, At3g07860, At3g07870, At3g07880, At3g07890,
At3g07900, At3g07910, At3g07960, At3g08020, At3g08510, At3g08520,
At3g08530, At3g08580, At3g08610, At3g08680, At3g08710, At3g08720,
At3g08730, At3g08780, At3g08790, At3g08910, At3g08920, At3g08940,
At3g08943, At3g08950, At3g08960, At3g08970, At3g09000, At3g09010,
At3g09030, At3g09200, At3g09300, At3g09340, At3g09350, At3g09360,
At3g09370, At3g09440, At3g09480, At3g09490, At3g09505, At3g09520,
At3g09540, At3g09760, At3g09820, At3g09830, At3g09920, At3g09980,
At3g10020, At3g10160, At3g10250, At3g10300, At3g10320, At3g10330,
At3g10380, At3g10390, At3g10415, At3g10420, At3g10490, At3g10500,
At3g10572, At3g10640, At3g10650, At3g10670, At3g10680, At3g10720,
At3g10770, At3g10790, At3g10840, At3g10845, At3g10880, At3g10915,
At3g10920, At3g10970, At3g10980, At3g11220, At3g11230, At3g11280,
At3g11460, At3g11510, At3g11560, At3g11570, At3g11700, At3g11760,
At3g11820, At3g11910, At3g11940, At3g12030, At3g12110, At3g12120,
At3g12130, At3g12210, At3g12240, At3g12250, At3g12290, At3g12300,
At3g12320, At3g12350, At3g12380, At3g12385, At3g12390, At3g12560,
At3g12620, At3g12690, At3g12740, At3g12800, At3g12920, At3g12950,
At3g12980, At3g13030, At3g13040, At3g13050, At3g13180, At3g13229,
At3g13230, At3g13300, At3g13320, At3g13330, At3g13350, At3g13380,
At3g13430, At3g13445, At3g13450, At3g13480, At3g13570, At3g13580,
At3g13670, At3g13690, At3g13720, At3g13800, At3g13810, At3g13860,
At3g13910, At3g13930, At3g13940, At3g14050, At3g14060, At3g14080,
At3g14090, At3g14100, At3g14150, At3g14180, At3g14190, At3g14200,
At3g14220, At3g14240, At3g14270, At3g14350, At3g14410, At3g14415,
At3g14430, At3g14475, At3g14620, At3g14660, At3g14750, At3g14830,
At3g14840, At3g14870, At3g14880, At3g14940, At3g14990, At3g15000,
At3g15030, At3g15060, At3g15070, At3g15200, At3g15210, At3g15220,
At3g15350, At3g15355, At3g15357, At3g15360, At3g15420, At3g15430,
At3g15450, At3g15460, At3g15480, At3g15500, At3g15520, At3g15550,
At3g15580, At3g15620, At3g15630, At3g15640, At3g15660, At3g15710,
At3g15760, At3g15770, At3g15780, At3g15800, At3g15850, At3g15900,
At3g16000, At3g16010, At3g16090, At3g16100, At3g16150, At3g16200,
At3g16210, At3g16280, At3g16350, At3g16380, At3g16480, At3g16500,
At3g16510, At3g16570, At3g16720, At3g16770, At3g16785, At3g16800,
At3g16830, At3g16850, At3g16950, At3g16990, At3g17000, At3g17020,
At3g17040, At3g17090, At3g17120, At3g17130, At3g17160, At3g17205,
At3g17240, At3g17365, At3g17530, At3g17609, At3g17650, At3g17710,
At3g17800, At3g17820, At3g17840, At3g17880, At3g17910, At3g17930,
At3g18060, At3g18070, At3g18210, At3g18215, At3g18310, At3g18350,
At3g18360, At3g18420, At3g18500, At3g18520, At3g18690, At3g18770,
At3g18780, At3g18790, At3g18810, At3g18820, At3g18830, At3g18850,
At3g18860, At3g18930, At3g18950, At3g18955, At3g18990, At3g19000,
At3g19010, At3g19030, At3g19080, At3g19120, At3g19130, At3g19150,
At3g19400, At3g19420, At3g19540, At3g19590, At3g19770, At3g19810,
At3g19830, At3g19840, At3g19930, At3g19950, At3g19960, At3g19970,

At3g19980, At3g20240, At3g20260, At3g20270, At3g20280, At3g20340,
At3g20350, At3g20365, At3g20500, At3g20510, At3g20600, At3g20640,
At3g20660, At3g20710, At3g20720, At3g20885, At3g20890, At3g20900,
At3g20970, At3g21055, At3g21060, At3g21475, At3g21480, At3g21540,
At3g21650, At3g21710, At3g21730, At3g21760, At3g21810, At3g22100,
At3g22104, At3g22190, At3g22370, At3g22440, At3g22450, At3g22460,
At3g22750, At3g22770, At3g22780, At3g22845, At3g22880, At3g22886,
At3g22942, At3g22960, At3g23030, At3g23040, At3g23230, At3g23250,
At3g23300, At3g23340, At3g23440, At3g23540, At3g23590, At3g23600,
At3g23740, At3g23830, At3g23940, At3g23990, At3g24120, At3g24150,
At3g24160, At3g24190, At3g24503, At3g24550, At3g24560, At3g24570,
At3g24580, At3g24670, At3g24730, At3g24740, At3g24760, At3g24820,
At3g24830, At3g24860, At3g25070, At3g25110, At3g25120, At3g25410,
At3g25430, At3g25600, At3g25650, At3g25700, At3g25717, At3g25780,
At3g25805, At3g25880, At3g25910, At3g26030, At3g26060, At3g26115,
At3g26210, At3g26310, At3g26380, At3g26440, At3g26580, At3g26670,
At3g26680, At3g26730, At3g26740, At3g26744, At3g26810, At3g26840,
At3g26850, At3g27050, At3g27060, At3g27080, At3g27090, At3g27095,
At3g27100, At3g27160, At3g27210, At3g27260, At3g27340, At3g27380,
At3g27390, At3g27700, At3g27730, At3g27770, At3g27830, At3g27865,
At3g28330, At3g28340, At3g28410, At3g28560, At3g28730, At3g28760,
At3g28860, At3g28910, At3g28940, At3g29160, At3g29240, At3g29280,
At3g29290, At3g29320, At3g29360, At3g29390, At3g29810, At3g30460,
At3g30775, At3g31996, At3g33000, At3g33002, At3g42170, At3g42316,
At3g42950, At3g43230, At3g43310, At3g43510, At3g43580, At3g43600,
At3g43700, At3g43710, At3g43800, At3g44100, At3g44200, At3g44310,
At3g44320, At3g44330, At3g44380, At3g44400, At3g44800, At3g44820,
At3g45130, At3g45230, At3g45300, At3g45400, At3g45620, At3g45770,
At3g45830, At3g45930, At3g45970, At3g45980, At3g46000, At3g46160,
At3g46200, At3g46320, At3g46430, At3g46530, At3g46550, At3g46560,
At3g46580, At3g46590, At3g46630, At3g46640, At3g46690, At3g46790,
At3g46930, At3g47000, At3g47070, At3g47080, At3g47160, At3g47450,
At3g47460, At3g47470, At3g47490, At3g47540, At3g47550, At3g47560,
At3g47570, At3g47620, At3g47640, At3g47650, At3g47690, At3g47830,
At3g47833, At3g48000, At3g48050, At3g48140, At3g48150, At3g48170,
At3g48320, At3g48330, At3g48520, At3g48550, At3g48570, At3g48600,
At3g48730, At3g48750, At3g48770, At3g48800, At3g48810, At3g48820,
At3g48830, At3g48870, At3g48880, At3g48890, At3g48930, At3g48990,
At3g49000, At3g49080, At3g49210, At3g49220, At3g49240, At3g49250,
At3g49390, At3g49420, At3g49500, At3g49510, At3g49590, At3g49610,
At3g49620, At3g49670, At3g49680, At3g49710, At3g49750, At3g49800,
At3g49810, At3g49870, At3g49890, At3g49910, At3g49930, At3g49940,
At3g50060, At3g50080, At3g50240, At3g50270, At3g50310, At3g50360,
At3g50430, At3g50480, At3g50590, At3g50630, At3g50680, At3g50685,
At3g50750, At3g50790, At3g50880, At3g50920, At3g51080, At3g51130,
At3g51160, At3g51230, At3g51240, At3g51390, At3g51420, At3g51430,

At3g51450, At3g51490, At3g51500, At3g51520, At3g51550, At3g51620, At3g51630, At3g51730, At3g51820, At3g51830, At3g51890, At3g51910, At3g51920, At3g51950, At3g51960, At3g51970, At3g51980, At3g51990, At3g52040, At3g52060, At3g52070, At3g52090, At3g52100, At3g52105, At3g52190, At3g52220, At3g52230, At3g52400, At3g52430, At3g52480, At3g52525, At3g52590, At3g52610, At3g52670, At3g52840, At3g52880, At3g52910, At3g52940, At3g52960, At3g52990, At3g53000, At3g53090, At3g53130, At3g53220, At3g53260, At3g53280, At3g53310, At3g53350, At3g53360, At3g53540, At3g53610, At3g53620, At3g53630, At3g53670, At3g53740, At3g53750, At3g53760, At3g53810, At3g53930, At3g53950, At3g54000, At3g54010, At3g54040, At3g54140, At3g54300, At3g54360, At3g54400, At3g54420, At3g54440, At3g54460, At3g54480, At3g54490, At3g54600, At3g54670, At3g54680, At3g54740, At3g54800, At3g54810, At3g54880, At3g54890, At3g54900, At3g54910, At3g54920, At3g54950, At3g55000, At3g55170, At3g55180, At3g55220, At3g55270, At3g55280, At3g55380, At3g55430, At3g55440, At3g55470, At3g55520, At3g55530, At3g55570, At3g55605, At3g55620, At3g55980, At3g56000, At3g56010, At3g56020, At3g56030, At3g56060, At3g56080, At3g56140, At3g56170, At3g56190, At3g56200, At3g56250, At3g56270, At3g56275, At3g56290, At3g56330, At3g56350, At3g56360, At3g56400, At3g56650, At3g56710, At3g56720, At3g56820, At3g56850, At3g56860, At3g56880, At3g56950, At3g57070, At3g57130, At3g57140, At3g57270, At3g57280, At3g57290, At3g57300, At3g57340, At3g57370, At3g57410, At3g57430, At3g57450, At3g57480, At3g57550, At3g57640, At3g57645, At3g57670, At3g57730, At3g57760, At3g57800, At3g57810, At3g57880, At3g57890, At3g57910, At3g57920, At3g57930, At3g57950, At3g57980, At3g57990, At3g58030, At3g58130, At3g58200, At3g58430, At3g58490, At3g58510, At3g58540, At3g58560, At3g58580, At3g58670, At3g58700, At3g58830, At3g58840, At3g58970, At3g58975, At3g59000, At3g59050, At3g59060, At3g59100, At3g59110, At3g59210, At3g59290, At3g59300, At3g59650, At3g59690, At3g59700, At3g59950, At3g59960, At3g60030, At3g60080, At3g60110, At3g60190, At3g60230, At3g60250, At3g60400, At3g60480, At3g60500, At3g60520, At3g60540, At3g60620, At3g60690, At3g60800, At3g60820, At3g60830, At3g60900, At3g61010, At3g61020, At3g61080, At3g61100, At3g61105, At3g61110, At3g61130, At3g61150, At3g61280, At3g61320, At3g61460, At3g61520, At3g61560, At3g61580, At3g61600, At3g61630, At3g61640, At3g61670, At3g61690, At3g61740, At3g61820, At3g61850, At3g61870, At3g61890, At3g61980, At3g62030, At3g62090, At3g62120, At3g62150, At3g62200, At3g62260, At3g62280, At3g62285, At3g62290, At3g62390, At3g62400, At3g62410, At3g62470, At3g62475, At3g62550, At3g62580, At3g62640, At3g62650, At3g62700, At3g62735, At3g62790, At3g62830, At3g62840, At3g62860, At3g62950, At3g62970, At3g63006, At3g63010, At3g63060, At3g63070, At3g63090, At3g63100, At3g63120, At3g63130, At3g63170, At3g63310, At3g63460, At3g63470, At3g63500, At3g63510, At4g00100, At4g00150, At4g00305, At4g00310, At4g00335, At4g00340, At4g00440, At4g00500, At4g00530, At4g00570, At4g00585,

At4g00590, At4g00640, At4g00680, At4g00690, At4g00750, At4g00860, At4g00900, At4g01010, At4g01100, At4g01130, At4g01150, At4g01210, At4g01245, At4g01320, At4g01330, At4g01370, At4g01510, At4g01515, At4g01540, At4g01610, At4g01720, At4g01860, At4g01865, At4g01870, At4g01910, At4g01915, At4g01930, At4g01940, At4g01960, At4g02010, At4g02050, At4g02055, At4g02060, At4g02075, At4g02080, At4g02210, At4g02220, At4g02370, At4g02440, At4g02460, At4g02550, At4g02555, At4g02590, At4g02610, At4g02660, At4g02720, At4g02725, At4g02840, At4g02880, At4g02890, At4g02920, At4g03020, At4g03070, At4g03100, At4g03130, At4g03210, At4g03260, At4g03292, At4g03295, At4g03420, At4g03430, At4g03490, At4g03560, At4g04020, At4g04190, At4g04320, At4g04570, At4g04614, At4g04620, At4g04640, At4g04670, At4g04695, At4g04700, At4g04885, At4g04960, At4g04970, At4g05020, At4g05040, At4g05048, At4g05070, At4g05100, At4g05180, At4g05320, At4g05360, At4g05390, At4g05410, At4g05420, At4g05440, At4g05520, At4g05590, At4g06746, At4g08150, At4g08170, At4g08180, At4g08200, At4g08210, At4g08220, At4g08390, At4g08510, At4g08520, At4g08540, At4g08920, At4g08950, At4g08960, At4g08980, At4g09000, At4g09030, At4g09420, At4g09480, At4g09510, At4g09650, At4g09900, At4g09965, At4g09970, At4g10030, At4g10040, At4g10100, At4g10170, At4g10270, At4g10370, At4g10430, At4g10570, At4g10650, At4g10730, At4g10740, At4g10750, At4g10840, At4g10920, At4g11090, At4g11213, At4g11220, At4g11240, At4g11360, At4g11370, At4g11560, At4g11570, At4g11600, At4g11680, At4g11690, At4g11830, At4g11910, At4g12000, At4g12005, At4g12060, At4g12115, At4g12170, At4g12220, At4g12230, At4g12320, At4g12400, At4g12405, At4g12410, At4g12420, At4g12560, At4g12720, At4g12790, At4g12850, At4g13010, At4g13030, At4g13160, At4g13245, At4g13340, At4g13350, At4g13430, At4g13530, At4g13670, At4g13940, At4g13990, At4g14160, At4g14220, At4g14230, At4g14270, At4g14272, At4g14340, At4g14490, At4g14500, At4g14530, At4g14540, At4g14605, At4g14695, At4g14830, At4g14920, At4g15075, At4g15130, At4g15140, At4g15233, At4g15236, At4g15240, At4g15258, At4g15260, At4g15410, At4g15415, At4g15420, At4g15475, At4g15545, At4g15720, At4g15760, At4g15790, At4g15930, At4g15950, At4g16140, At4g16150, At4g16240, At4g16250, At4g16265, At4g16360, At4g16390, At4g16430, At4g16440, At4g16470, At4g16480, At4g16490, At4g16500, At4g16510, At4g16520, At4g16570, At4g16580, At4g16630, At4g16660, At4g16765, At4g16980, At4g17010, At4g17080, At4g17100, At4g17150, At4g17160, At4g17300, At4g17390, At4g17500, At4g17580, At4g17612, At4g17615, At4g17640, At4g17650, At4g17765, At4g17840, At4g17890, At4g17895, At4g17910, At4g17975, At4g18010, At4g18070, At4g18130, At4g18140, At4g18150, At4g18197, At4g18205, At4g18250, At4g18270, At4g18300, At4g18380, At4g18520, At4g18530, At4g18570, At4g18593, At4g18650, At4g18740, At4g18750, At4g18800, At4g18830, At4g18890, At4g18910, At4g18930, At4g18975, At4g19040, At4g19110, At4g19150, At4g19160, At4g19220, At4g19395, At4g19610, At4g19640, At4g19710, At4g19720, At4g19860, At4g19870,

At4g19940, At4g20000, At4g20010, At4g20200, At4g20280, At4g20290,
At4g20320, At4g20360, At4g20820, At4g20830, At4g20880, At4g21190,
At4g21445, At4g21510, At4g21570, At4g21670, At4g21730, At4g21940,
At4g21980, At4g21990, At4g22200, At4g22240, At4g22290, At4g22330,
At4g22340, At4g22350, At4g22360, At4g22380, At4g22580, At4g22590,
At4g22690, At4g22756, At4g22760, At4g22860, At4g22910, At4g22920,
At4g22930, At4g22960, At4g22970, At4g23050, At4g23190, At4g23220,
At4g23250, At4g23270, At4g23280, At4g23530, At4g23630, At4g23730,
At4g23885, At4g23910, At4g23915, At4g24020, At4g24040, At4g24090,
At4g24100, At4g24110, At4g24210, At4g24220, At4g24280, At4g24290,
At4g24370, At4g24390, At4g24400, At4g24470, At4g24480, At4g24560,
At4g24570, At4g24630, At4g24690, At4g24700, At4g24740, At4g24920,
At4g24960, At4g24972, At4g25020, At4g25050, At4g25090, At4g25100,
At4g25210, At4g25230, At4g25360, At4g25380, At4g25470, At4g25490,
At4g25540, At4g25550, At4g25580, At4g25620, At4g25670, At4g25680,
At4g25770, At4g25780, At4g25840, At4g25870, At4g25910, At4g26000,
At4g26070, At4g26080, At4g26090, At4g26130, At4g26170, At4g26225,
At4g26230, At4g26490, At4g26570, At4g26675, At4g26680, At4g26700,
At4g26840, At4g26850, At4g26860, At4g26870, At4g26970, At4g26990,
At4g27020, At4g27030, At4g27050, At4g27100, At4g27120, At4g27270,
At4g27290, At4g27410, At4g27460, At4g27470, At4g27560, At4g27657,
At4g27700, At4g27710, At4g27740, At4g27780, At4g27830, At4g27840,
At4g27875, At4g27880, At4g27910, At4g27920, At4g27950, At4g27970,
At4g28030, At4g28040, At4g28070, At4g28260, At4g28290, At4g28300,
At4g28310, At4g28400, At4g28450, At4g28460, At4g28490, At4g28540,
At4g28630, At4g28640, At4g28720, At4g28760, At4g29010, At4g29050,
At4g29060, At4g29070, At4g29090, At4g29120, At4g29160, At4g29170,
At4g29180, At4g29190, At4g29210, At4g29230, At4g29310, At4g29380,
At4g29390, At4g29400, At4g29410, At4g29415, At4g29530, At4g29660,
At4g29710, At4g29730, At4g29780, At4g29810, At4g29860, At4g29870,
At4g29950, At4g30000, At4g30010, At4g30190, At4g30200, At4g30220,
At4g30280, At4g30340, At4g30350, At4g30440, At4g30450, At4g30500,
At4g30580, At4g30600, At4g30650, At4g30680, At4g30810, At4g30840,
At4g30850, At4g30890, At4g30900, At4g30970, At4g30972, At4g30993,
At4g30996, At4g31130, At4g31150, At4g31160, At4g31180, At4g31300,
At4g31330, At4g31410, At4g31450, At4g31510, At4g31520, At4g31530,
At4g31550, At4g31560, At4g31670, At4g31700, At4g31740, At4g31800,
At4g31830, At4g31840, At4g31875, At4g31877, At4g31960, At4g32020,
At4g32050, At4g32150, At4g32190, At4g32285, At4g32290, At4g32300,
At4g32320, At4g32330, At4g32400, At4g32410, At4g32450, At4g32470,
At4g32475, At4g32480, At4g32530, At4g32620, At4g32640, At4g32660,
At4g32670, At4g32680, At4g32690, At4g32730, At4g32750, At4g32760,
At4g32765, At4g32770, At4g32850, At4g32870, At4g32910, At4g32930,
At4g32960, At4g33050, At4g33060, At4g33080, At4g33130, At4g33170,
At4g33300, At4g33430, At4g33440, At4g33565, At4g33625, At4g33650,
At4g33666, At4g33740, At4g33920, At4g33925, At4g33980, At4g34000,

At4g34035, At4g34040, At4g34050, At4g34120, At4g34131, At4g34138, At4g34140, At4g34150, At4g34240, At4g34290, At4g34310, At4g34390, At4g34480, At4g34490, At4g34500, At4g34550, At4g34560, At4g34590, At4g34620, At4g34630, At4g34640, At4g34700, At4g34710, At4g34740, At4g34770, At4g34780, At4g34870, At4g34910, At4g34975, At4g35000, At4g35040, At4g35050, At4g35070, At4g35140, At4g35220, At4g35310, At4g35320, At4g35335, At4g35380, At4g35440, At4g35460, At4g35470, At4g35490, At4g35570, At4g35580, At4g35590, At4g35610, At4g35620, At4g35750, At4g35760, At4g35770, At4g35785, At4g35790, At4g35800, At4g35830, At4g35860, At4g35930, At4g35980, At4g36020, At4g36060, At4g36245, At4g36280, At4g36500, At4g36540, At4g36670, At4g36710, At4g36720, At4g36750, At4g36780, At4g36790, At4g36810, At4g36820, At4g36900, At4g36930, At4g36960, At4g37040, At4g37080, At4g37180, At4g37210, At4g37235, At4g37240, At4g37270, At4g37300, At4g37440, At4g37450, At4g37470, At4g37480, At4g37610, At4g37620, At4g37630, At4g37640, At4g37670, At4g37690, At4g37740, At4g37830, At4g37910, At4g38040, At4g38060, At4g38070, At4g38140, At4g38170, At4g38210, At4g38225, At4g38280, At4g38310, At4g38320, At4g38470, At4g38550, At4g38580, At4g38620, At4g38690, At4g38730, At4g38780, At4g38790, At4g38810, At4g38920, At4g38930, At4g38960, At4g38970, At4g39040, At4g39080, At4g39090, At4g39100, At4g39150, At4g39160, At4g39320, At4g39340, At4g39350, At4g39360, At4g39361, At4g39390, At4g39400, At4g39410, At4g39560, At4g39600, At4g39610, At4g39615, At4g39620, At4g39630, At4g39640, At4g39680, At4g39730, At4g39850, At4g39865, At4g39870, At4g39880, At4g40030, At4g40040, At4g40042, At4g40070, At5g01070, At5g01200, At5g01260, At5g01390, At5g01420, At5g01460, At5g01470, At5g01530, At5g01600, At5g01630, At5g01650, At5g01715, At5g01720, At5g01760, At5g01810, At5g01950, At5g01960, At5g02020, At5g02150, At5g02160, At5g02200, At5g02250, At5g02290, At5g02400, At5g02480, At5g02490, At5g02610, At5g02615, At5g02620, At5g02770, At5g02850, At5g02860, At5g02870, At5g02940, At5g02950, At5g02960, At5g02970, At5g03030, At5g03130, At5g03190, At5g03210, At5g03220, At5g03230, At5g03300, At5g03310, At5g03320, At5g03420, At5g03452, At5g03460, At5g03480, At5g03555, At5g03630, At5g03650, At5g03670, At5g03705, At5g03730, At5g03740, At5g03850, At5g03905, At5g03920, At5g03970, At5g03980, At5g03990, At5g04030, At5g04060, At5g04070, At5g04170, At5g04340, At5g04410, At5g04420, At5g04440, At5g04490, At5g04500, At5g04590, At5g04710, At5g04720, At5g04780, At5g04900, At5g05090, At5g05100, At5g05140, At5g05180, At5g05300, At5g05310, At5g05380, At5g05430, At5g05440, At5g05560, At5g05580, At5g05780, At5g05795, At5g05810, At5g05860, At5g05920, At5g06060, At5g06070, At5g06125, At5g06320, At5g06390, At5g06450, At5g06460, At5g06570, At5g06700, At5g06830, At5g06860, At5g06960, At5g06970, At5g06980, At5g07020, At5g07060, At5g07100, At5g07120, At5g07220, At5g07240, At5g07290, At5g07320, At5g07340, At5g07360, At5g07370, At5g07580, At5g07625, At5g07670, At5g07675, At5g07680, At5g07710, At5g07770,

At5g07790, At5g07920, At5g08075, At5g08080, At5g08139, At5g08150, At5g08185, At5g08190, At5g08300, At5g08335, At5g08360, At5g08400, At5g08415, At5g08420, At5g08440, At5g08450, At5g08460, At5g08500, At5g08570, At5g08630, At5g08660, At5g08670, At5g08680, At5g09300, At5g09400, At5g09540, At5g09590, At5g09620, At5g09630, At5g09640, At5g09755, At5g09760, At5g09770, At5g09800, At5g09810, At5g09880, At5g09900, At5g09910, At5g10050, At5g10070, At5g10080, At5g10110, At5g10160, At5g10290, At5g10350, At5g10450, At5g10455, At5g10490, At5g10525, At5g10540, At5g10560, At5g10730, At5g10780, At5g10820, At5g10830, At5g10840, At5g10850, At5g10950, At5g10960, At5g10990, At5g11010, At5g11030, At5g11040, At5g11060, At5g11090, At5g11140, At5g11170, At5g11200, At5g11230, At5g11240, At5g11260, At5g11390, At5g11420, At5g11475, At5g11480, At5g11490, At5g11510, At5g11520, At5g11650, At5g11670, At5g11720, At5g11740, At5g11750, At5g11850, At5g11900, At5g12060, At5g12150, At5g12190, At5g12230, At5g12340, At5g12390, At5g12420, At5g12430, At5g12440, At5g12470, At5g12850, At5g12900, At5g12970, At5g12980, At5g12990, At5g13000, At5g13020, At5g13030, At5g13090, At5g13100, At5g13120, At5g13160, At5g13180, At5g13190, At5g13200, At5g13220, At5g13225, At5g13230, At5g13250, At5g13300, At5g13310, At5g13360, At5g13370, At5g13500, At5g13630, At5g13640, At5g13700, At5g13710, At5g13750, At5g13760, At5g13770, At5g13810, At5g13840, At5g13845, At5g13890, At5g14030, At5g14050, At5g14060, At5g14100, At5g14105, At5g14110, At5g14120, At5g14130, At5g14140, At5g14170, At5g14200, At5g14260, At5g14310, At5g14360, At5g14430, At5g14470, At5g14495, At5g14610, At5g14640, At5g14700, At5g14710, At5g14730, At5g14790, At5g14930, At5g15090, At5g15200, At5g15210, At5g15220, At5g15240, At5g15340, At5g15420, At5g15440, At5g15470, At5g15500, At5g15510, At5g15620, At5g15640, At5g15700, At5g15710, At5g15800, At5g15850, At5g15860, At5g15880, At5g15950, At5g15980, At5g16010, At5g16110, At5g16170, At5g16180, At5g16260, At5g16360, At5g16390, At5g16440, At5g16480, At5g16560, At5g16610, At5g16620, At5g16640, At5g16730, At5g16750, At5g16760, At5g16800, At5g16840, At5g16850, At5g16870, At5g16880, At5g17190, At5g17250, At5g17280, At5g17290, At5g17370, At5g17380, At5g17610, At5g17620, At5g17630, At5g17840, At5g17910, At5g17920, At5g17990, At5g18120, At5g18170, At5g18270, At5g18430, At5g18440, At5g18480, At5g18520, At5g18610, At5g18630, At5g18650, At5g18660, At5g18840, At5g18850, At5g18950, At5g18960, At5g19095, At5g19120, At5g19130, At5g19140, At5g19150, At5g19230, At5g19240, At5g19250, At5g19290, At5g19300, At5g19430, At5g19460, At5g19500, At5g19570, At5g19660, At5g19670, At5g19690, At5g19860, At5g19980, At5g20010, At5g20020, At5g20030, At5g20060, At5g20070, At5g20120, At5g20170, At5g20180, At5g20190, At5g20230, At5g20250, At5g20650, At5g20660, At5g20680, At5g20830, At5g20900, At5g20910, At5g20950, At5g21170, At5g21940, At5g22000, At5g22010, At5g22040, At5g22210, At5g22250, At5g22290, At5g22630, At5g22640, At5g22730, At5g22770, At5g22780, At5g22830, At5g22840,

At5g22880, At5g22910, At5g22920, At5g22950, At5g22990, At5g23070, At5g23210, At5g23470, At5g23570, At5g23610, At5g23630, At5g23670, At5g23690, At5g23700, At5g23730, At5g23850, At5g24010, At5g24070, At5g24165, At5g24180, At5g24310, At5g24316, At5g24320, At5g24330, At5g24340, At5g24360, At5g24380, At5g24400, At5g24470, At5g24520, At5g24590, At5g24630, At5g24640, At5g24680, At5g24710, At5g24810, At5g24870, At5g24910, At5g24930, At5g25100, At5g25130, At5g25220, At5g25290, At5g25350, At5g25460, At5g25520, At5g25540, At5g25580, At5g25625, At5g25630, At5g25757, At5g25760, At5g25790, At5g25930, At5g25940, At5g26030, At5g26220, At5g26360, At5g26680, At5g26720, At5g26742, At5g26805, At5g26820, At5g26940, At5g27030, At5g27150, At5g27320, At5g27640, At5g27700, At5g27715, At5g27720, At5g28410, At5g28770, At5g28840, At5g29000, At5g30490, At5g30495, At5g32450, At5g33280, At5g33290, At5g35100, At5g35220, At5g35360, At5g35430, At5g35570, At5g35735, At5g35840, At5g35923, At5g36110, At5g36160, At5g36290, At5g36880, At5g37010, At5g37260, At5g37600, At5g38030, At5g38035, At5g38150, At5g38155, At5g38210, At5g38220, At5g38240, At5g38470, At5g38640, At5g38830, At5g38850, At5g38895, At5g39010, At5g39020, At5g39050, At5g39070, At5g39080, At5g39240, At5g39250, At5g39330, At5g39510, At5g39660, At5g39790, At5g39800, At5g39830, At5g39840, At5g39860, At5g39880, At5g39950, At5g39960, At5g40140, At5g40170, At5g40180, At5g40340, At5g40370, At5g40390, At5g40460, At5g40550, At5g40660, At5g40670, At5g40850, At5g41080, At5g41100, At5g41140, At5g41170, At5g41190, At5g41350, At5g41370, At5g41471, At5g41600, At5g41670, At5g41675, At5g41680, At5g41960, At5g41990, At5g42000, At5g42010, At5g42020, At5g42050, At5g42060, At5g42130, At5g42160, At5g42270, At5g42300, At5g42520, At5g42540, At5g42635, At5g42700, At5g42790, At5g42900, At5g42950, At5g42990, At5g43100, At5g43130, At5g43140, At5g43170, At5g43330, At5g43560, At5g43600, At5g43700, At5g43750, At5g43760, At5g43820, At5g43900, At5g43940, At5g43960, At5g43970, At5g44070, At5g44170, At5g44180, At5g44190, At5g44280, At5g44286, At5g44290, At5g44610, At5g44785, At5g44790, At5g45020, At5g45030, At5g45110, At5g45370, At5g45430, At5g45610, At5g45660, At5g45750, At5g45790, At5g45810, At5g45990, At5g46115, At5g46280, At5g46380, At5g46390, At5g46410, At5g46420, At5g46470, At5g46590, At5g46780, At5g46800, At5g46810, At5g46840, At5g47010, At5g47040, At5g47120, At5g47140, At5g47170, At5g47175, At5g47180, At5g47190, At5g47210, At5g47220, At5g47320, At5g47380, At5g47400, At5g47420, At5g47480, At5g47590, At5g47610, At5g47640, At5g47760, At5g47770, At5g47780, At5g47790, At5g47820, At5g47850, At5g47910, At5g48020, At5g48220, At5g48240, At5g48250, At5g48330, At5g48380, At5g48385, At5g48450, At5g48560, At5g48580, At5g48620, At5g48840, At5g48930, At5g49000, At5g49110, At5g49200, At5g49210, At5g49450, At5g49470, At5g49500, At5g49510, At5g49520, At5g49710, At5g49730, At5g49760, At5g49810, At5g49900, At5g49980, At5g50000, At5g50100, At5g50170, At5g50380, At5g50440, At5g50450, At5g50460, At5g50780,

At5g50805, At5g50890, At5g50920, At5g51010, At5g51070, At5g51130, At5g51170, At5g51180, At5g51190, At5g51230, At5g51260, At5g51340, At5g51370, At5g51390, At5g51460, At5g51550, At5g51640, At5g51870, At5g51880, At5g51900, At5g51960, At5g52050, At5g52055, At5g52180, At5g52200, At5g52210, At5g52240, At5g52440, At5g52510, At5g52530, At5g52560, At5g52650, At5g52770, At5g52800, At5g52820, At5g52830, At5g52900, At5g52930, At5g52980, At5g52990, At5g53050, At5g53290, At5g53300, At5g53310, At5g53320, At5g53330, At5g53400, At5g53440, At5g53450, At5g53470, At5g53480, At5g53490, At5g53560, At5g53580, At5g53640, At5g53650, At5g53830, At5g53980, At5g54110, At5g54160, At5g54180, At5g54580, At5g54650, At5g54770, At5g54910, At5g54920, At5g54930, At5g54940, At5g54960, At5g54970, At5g55040, At5g55045, At5g55130, At5g55131, At5g55132, At5g55220, At5g55505, At5g55520, At5g55630, At5g55670, At5g55740, At5g55830, At5g55850, At5g55920, At5g55950, At5g55960, At5g56020, At5g56075, At5g56130, At5g56140, At5g56150, At5g56170, At5g56180, At5g56230, At5g56260, At5g56270, At5g56360, At5g56450, At5g56530, At5g56550, At5g56580, At5g56630, At5g56670, At5g56680, At5g56750, At5g56990, At5g57000, At5g57040, At5g57080, At5g57160, At5g57170, At5g57180, At5g57220, At5g57270, At5g57290, At5g57300, At5g57310, At5g57345, At5g57490, At5g57570, At5g57580, At5g57630, At5g57650, At5g57655, At5g57660, At5g57670, At5g57885, At5g57887, At5g57930, At5g57940, At5g58005, At5g58070, At5g58130, At5g58140, At5g58190, At5g58200, At5g58220, At5g58230, At5g58240, At5g58250, At5g58320, At5g58350, At5g58370, At5g58375, At5g58380, At5g58430, At5g58440, At5g58560, At5g58570, At5g58575, At5g58595, At5g58630, At5g58640, At5g58650, At5g58700, At5g58710, At5g58720, At5g58740, At5g58787, At5g58870, At5g58950, At5g59020, At5g59060, At5g59070, At5g59140, At5g59150, At5g59290, At5g59300, At5g59380, At5g59430, At5g59440, At5g59460, At5g59470, At5g59490, At5g59540, At5g59550, At5g59570, At5g59680, At5g59690, At5g59760, At5g59780, At5g59820, At5g59840, At5g59890, At5g59900, At5g59910, At5g59945, At5g59950, At5g59970, At5g59980, At5g59990, At5g60110, At5g60270, At5g60290, At5g60360, At5g60390, At5g60450, At5g60540, At5g60550, At5g60580, At5g60610, At5g60630, At5g60740, At5g60750, At5g60790, At5g60920, At5g60966, At5g60980, At5g61000, At5g61180, At5g61210, At5g61240, At5g61330, At5g61345, At5g61350, At5g61380, At5g61450, At5g61490, At5g61520, At5g61600, At5g61620, At5g61670, At5g61820, At5g61830, At5g61880, At5g62000, At5g62060, At5g62070, At5g62090, At5g62150, At5g62190, At5g62270, At5g62300, At5g62370, At5g62530, At5g62560, At5g62575, At5g62620, At5g62810, At5g62820, At5g62910, At5g62920, At5g62930, At5g63060, At5g63080, At5g63120, At5g63130, At5g63160, At5g63190, At5g63200, At5g63280, At5g63290, At5g63330, At5g63370, At5g63390, At5g63410, At5g63420, At5g63490, At5g63500, At5g63510, At5g63530, At5g63640, At5g63680, At5g63790, At5g63800, At5g63810, At5g63860, At5g63870, At5g63890, At5g63900, At5g63970, At5g63980, At5g64040, At5g64070, At5g64080, At5g64090,

At5g64170, At5g64180, At5g64200, At5g64250, At5g64260, At5g64270, At5g64300, At5g64310, At5g64430, At5g64450, At5g64460, At5g64470, At5g64505, At5g64510, At5g64550, At5g64650, At5g64670, At5g64680, At5g64710, At5g64735, At5g64780, At5g64790, At5g64840, At5g64850, At5g64920, At5g64940, At5g65015, At5g65110, At5g65120, At5g65207, At5g65300, At5g65360, At5g65380, At5g65430, At5g65445, At5g65450, At5g65470, At5g65540, At5g65615, At5g65620, At5g65630, At5g65650, At5g65670, At5g65700, At5g65720, At5g65810, At5g65820, At5g65830, At5g65860, At5g65900, At5g65910, At5g65920, At5g66070, At5g66100, At5g66120, At5g66130, At5g66180, At5g66190, At5g66200, At5g66210, At5g66220, At5g66270, At5g66280, At5g66320, At5g66350, At5g66400, At5g66410, At5g66470, At5g66480, At5g66490, At5g66562, At5g66564, At5g66580, At5g66590, At5g66620, At5g66658, At5g66675, At5g66750, At5g66770, At5g66780, At5g66810, At5g66850, At5g66870, At5g66900, At5g66930, At5g67060, At5g67070, At5g67090, At5g67100, At5g67130, At5g67140, At5g67160, At5g67220, At5g67230, At5g67280, At5g67290, At5g67300, At5g67330, At5g67350, At5g67370, At5g67455, At5g67470, At5g67480, At5g67490, At5g67500, At5g67510, At5g67520, At5g67560, At5g67600, At5g67620, At5g67630
