# Supplementary material for: Whole-Genome Analysis of Histone H3 Lysine 27 Trimethylation in Arabidopsis
Source: PLoS Biol. 2007 Apr 17;5(5):e129. doi: 10.1371/journal.pbio.0050129 (PMC1852588; doi:10.1371/journal.pbio.0050129)
Supplement: Supplementary file 5 [file pbio.0050129.sg003.pdf]

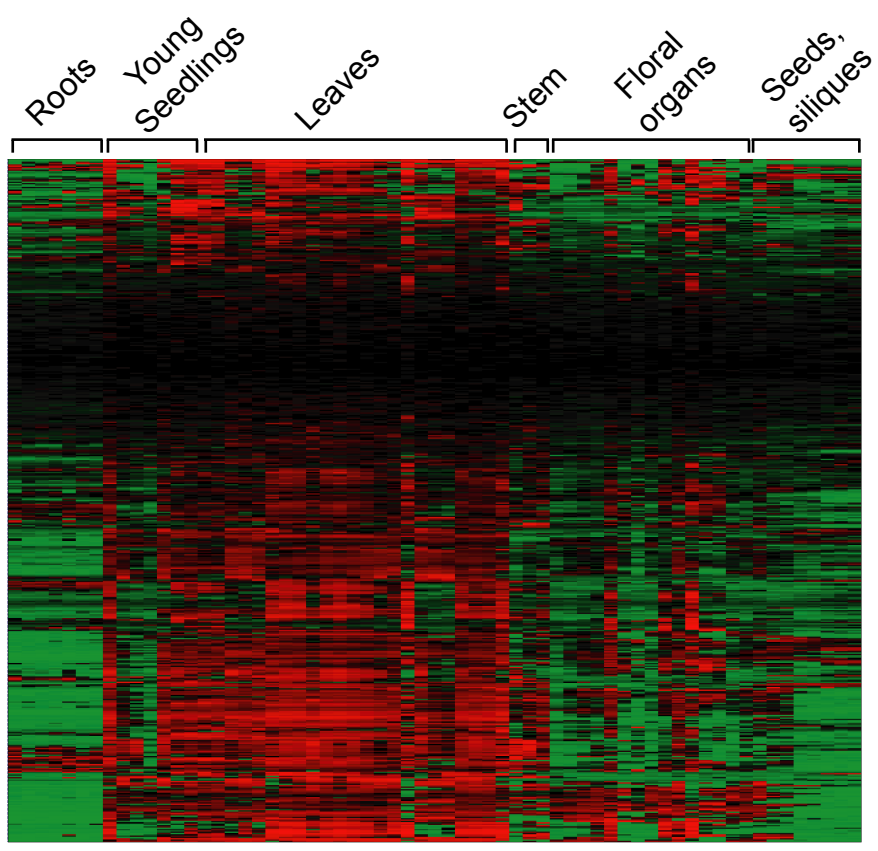

GO Term	% in Genome	% in Cluster	p-value	definition
GO:0006952	1.9%	8.9%	10^{-30}	defense response
GO:0016020	23.5%	38.3%	10^{-30}	membrane
GO:0012505	14.6%	25.6%	$10^{-10.9}$	endomembrane system
GO:0050896	8.9%	20.2%	$10^{-10.5}$	response to stimulus
GO:0004888	0.7%	3.7%	$10^{-8.6}$	transmembrane receptor activity
GO:0003700	6.3%	13.2%	$10^{-8.3}$	transcription factor activity

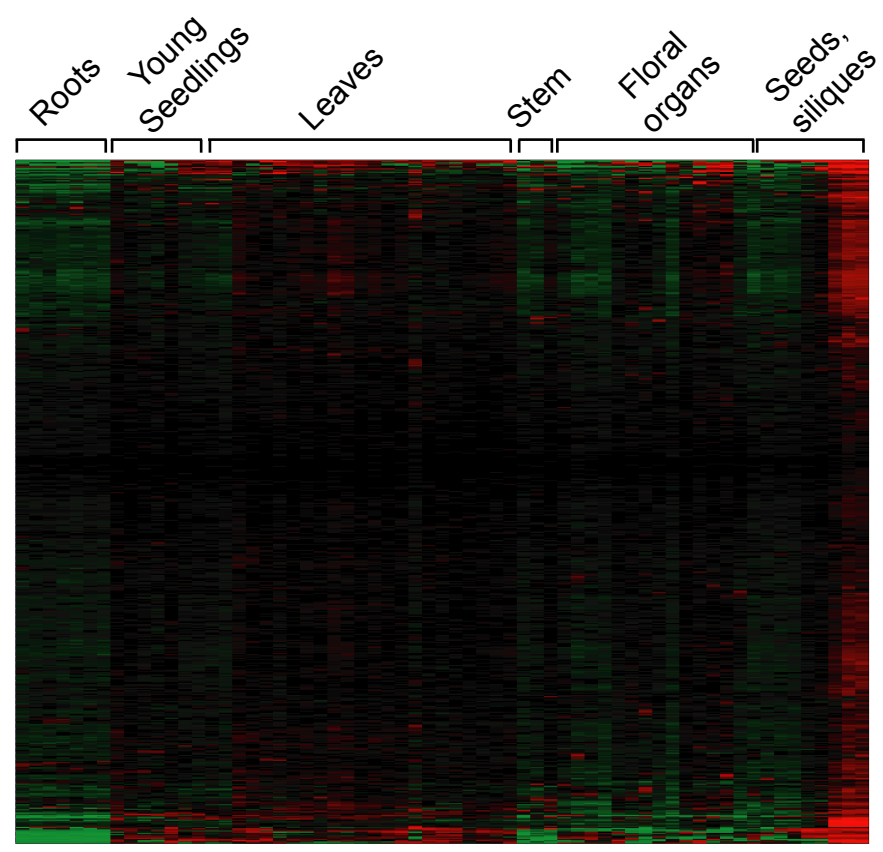

GO Term	% in Genome	% in Cluster	p-value	definition
GO:0006350	6.0%	11.6%	$10^{-6.6}$	transcription
GO:0045449	5.7%	10.9%	$10^{-6.1}$	regulation of transcription
GO:0019219	5.8%	10.9%	$10^{-6.0}$	regulation of nucleobase, nucleoside, nucleotide and nucleic acid metabolism
GO:0031323	5.9%	11.1%	$10^{-5.9}$	regulation of cellular metabolism
GO:0019222	6.0%	11.1%	$10^{-5.7}$	regulation of metabolism
GO:0006355	3.5%	7.5%	$10^{-5.6}$	regulation of transcription, DNA-dependent

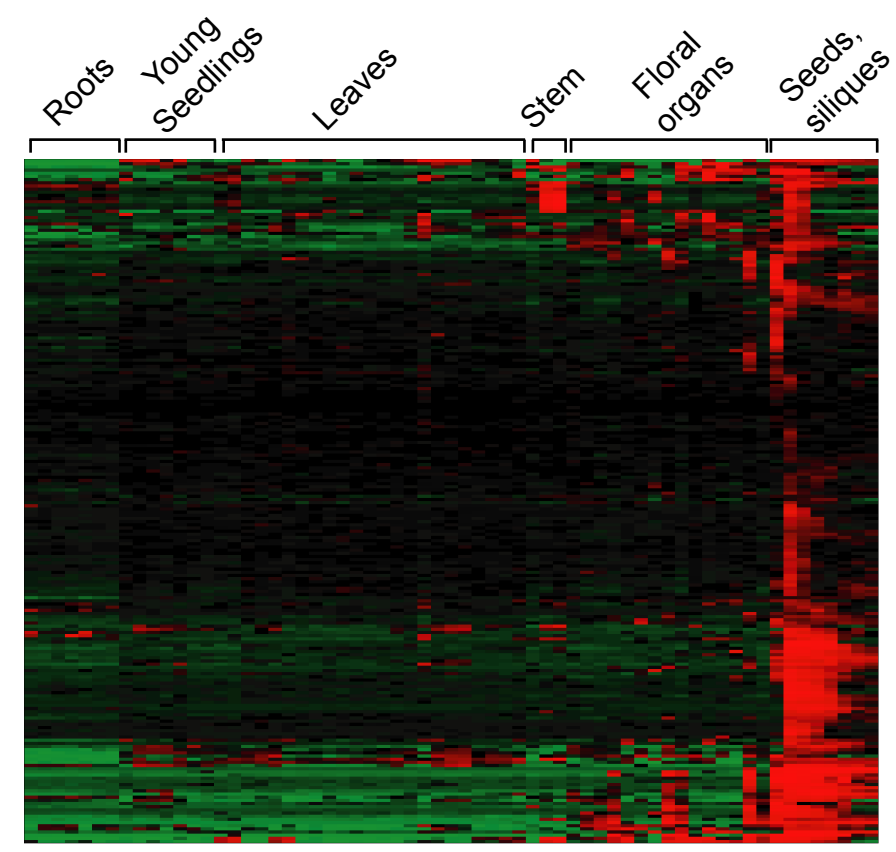

GO Term	% in Genome	% in Cluster	p-value	definition
GO:0004126	0.0%	2.3%	$10^{-8.0}$	cytidine deaminase activity
GO:0019005	0.1%	2.8%	$10^{-7.5}$	SCF ubiquitin ligase complex
GO:0031461	0.1%	2.8%	$10^{-7.3}$	cullin-RING ubiquitin ligase complex
GO:0019239	0.1%	2.3%	$10^{-7.3}$	deaminase activity
GO:0030528	6.8%	17.6%	$10^{-7.2}$	transcription regulator activity
GO:0003700	6.3%	16.2%	$10^{-6.7}$	transcription factor activity

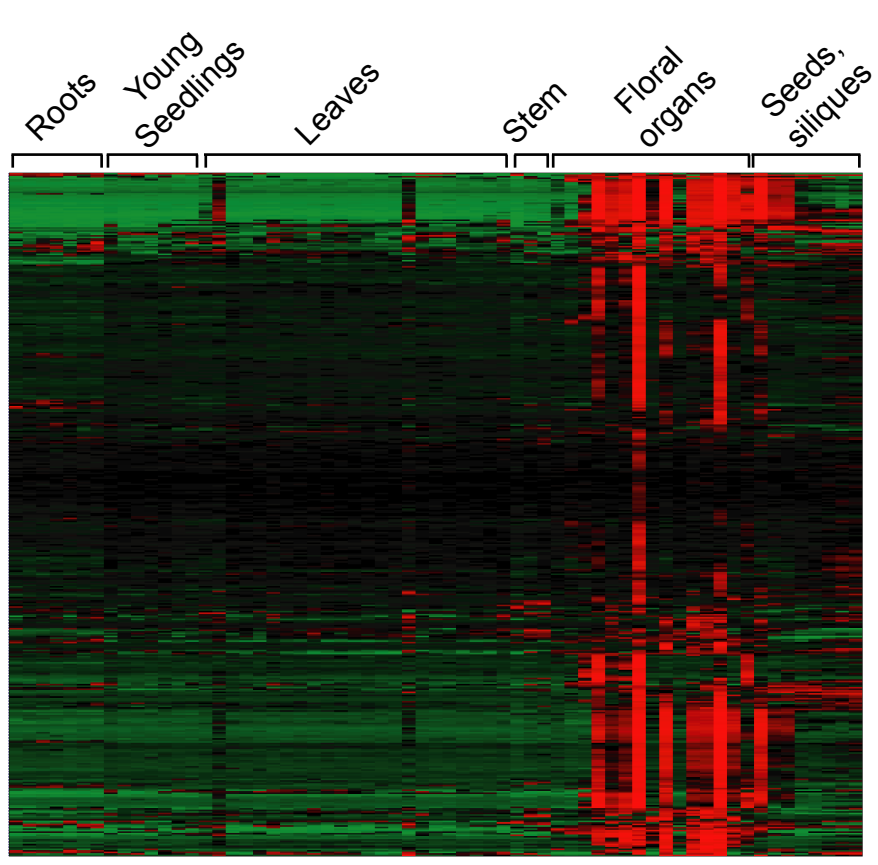

GO Term	% in Genome	% in Cluster	p-value	definition
GO:0012505	14.6%	26.8%	10^{-30}	endomembrane system
GO:0016020	23.5%	39.3%	10^{-30}	membrane
GO:0015385	0.1%	2.0%	$10^{-8.0}$	sodium:hydrogen antiporter activity
GO:0004650	0.3%	2.5%	$10^{-7.7}$	polygalacturonase activity
GO:0004553	1.4%	5.5%	$10^{-7.7}$	hydrolase activity, hydrolyzing O-glycosyl compounds
GO:0005451	0.2%	2.0%	$10^{-7.0}$	monovalent cation:proton antiporter activity

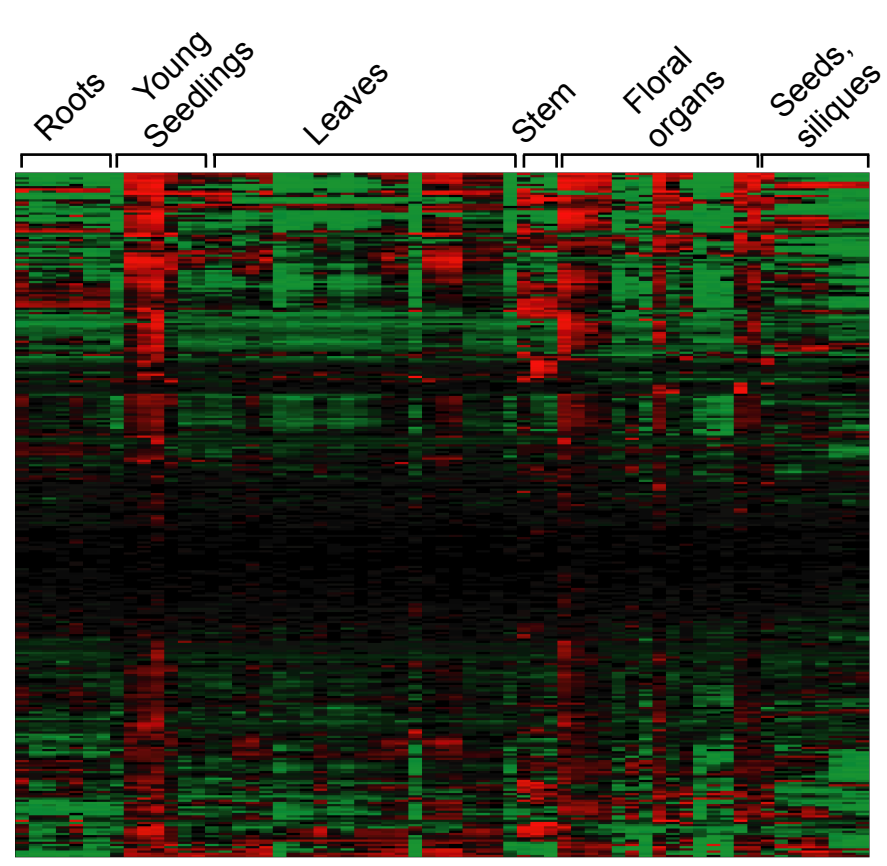

GO Term	% in Genome	% in Cluster	p-value	definition
GO:0003677	8.1%	23.5%	10^{-30}	DNA binding
GO:0003700	6.3%	21.9%	10^{-30}	transcription factor activity
GO:0006350	6.0%	16.8%	10^{-30}	transcription
GO:0007389	0.2%	3.9%	10^{-30}	pattern specification
GO:0003676	10.8%	25.8%	$10^{-10.9}$	nucleic acid binding
GO:0030528	6.8%	23.2%	$10^{-10.8}$	transcription regulator activity

Supplemental Figure 3
