# Supplementary material for: Whole-Genome Analysis of Histone H3 Lysine 27 Trimethylation in Arabidopsis
Source: PLoS Biol. 2007 Apr 17;5(5):e129. doi: 10.1371/journal.pbio.0050129 (PMC1852588; doi:10.1371/journal.pbio.0050129)
Supplement: Supplementary file 9 [file pbio.0050129.sg007.pdf]

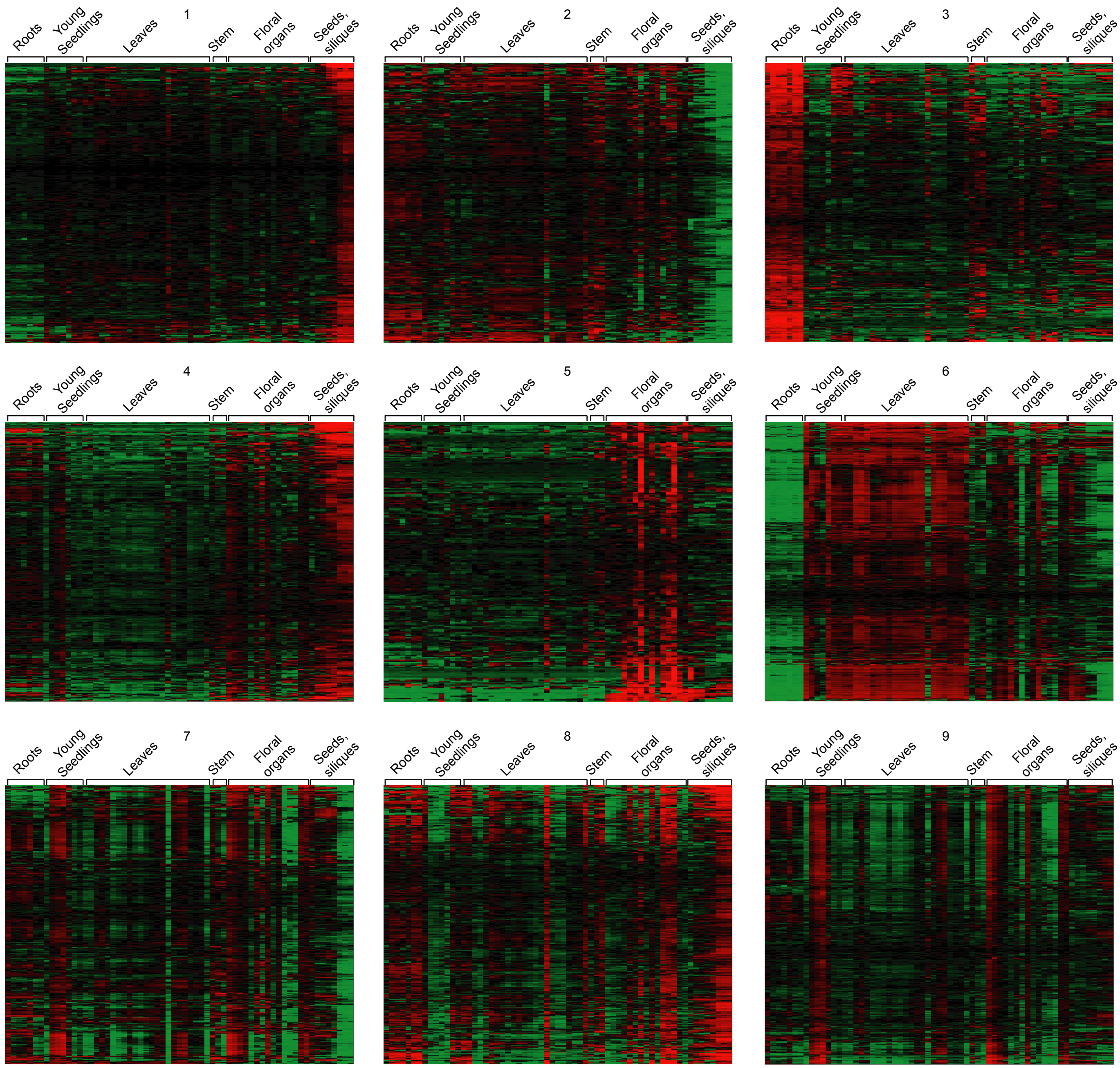

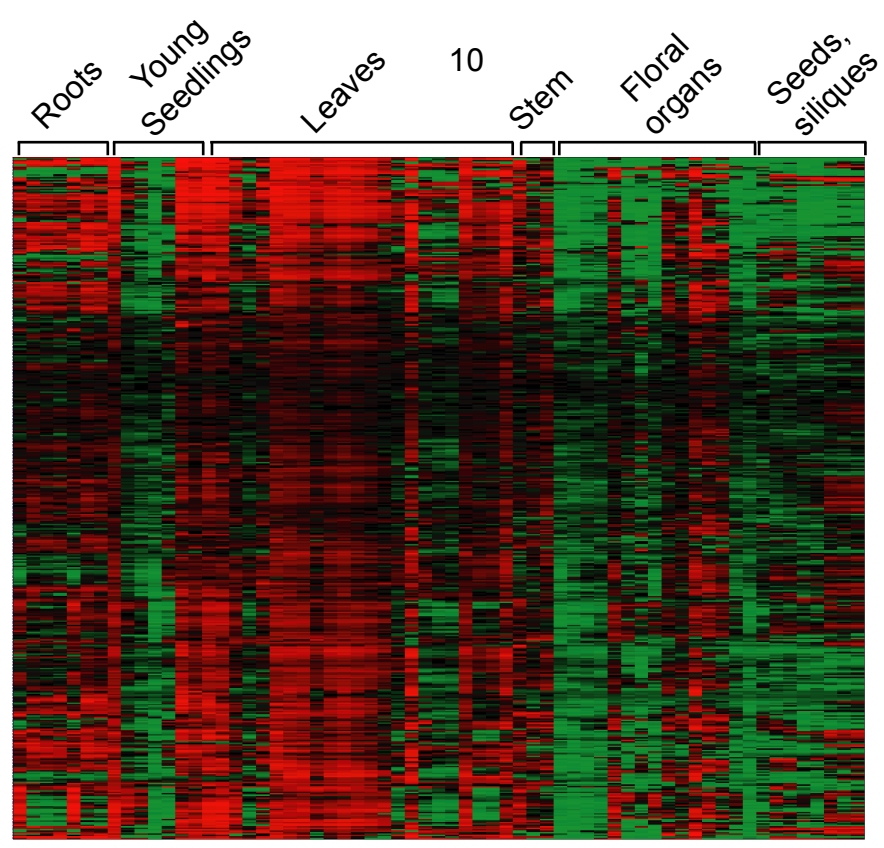

Cluster	GO Term	p-val (log10)	% in genome	% in cluster	definition
1	GO:0008020	-2.91	0.02%	0.61%	G-protein coupled photoreceptor activity
2	GO:0007582	-9.76	42.04%	59.68%	physiological process
3	GO:0003824	-3.86	29.00%	38.51%	catalytic activity
4	GO:0003676	-6.10	10.83%	19.30%	nucleic acid binding
5	GO:0031325	-4.00	0.11%	1.78%	positive regulation of cellular metabolism
6	GO:0005622	-30.00	38.07%	68.24%	intracellular
7	GO:0043234	-30.00	5.38%	15.75%	protein complex
8	GO:0006914	-8.44	0.08%	1.57%	autophagy
9	GO:0006412	-30.00	2.30%	8.75%	protein biosynthesis
10	GO:0002217	-30.00	0.68%	5.27%	physiological defense response

Supplemental Figure 7
