# Supplementary material for: Whole-Genome Analysis of Histone H3 Lysine 27 Trimethylation in Arabidopsis
Source: PLoS Biol. 2007 Apr 17;5(5):e129. doi: 10.1371/journal.pbio.0050129 (PMC1852588; doi:10.1371/journal.pbio.0050129)
Supplement: Supplementary file 10 [file pbio.0050129.sg008.pdf]

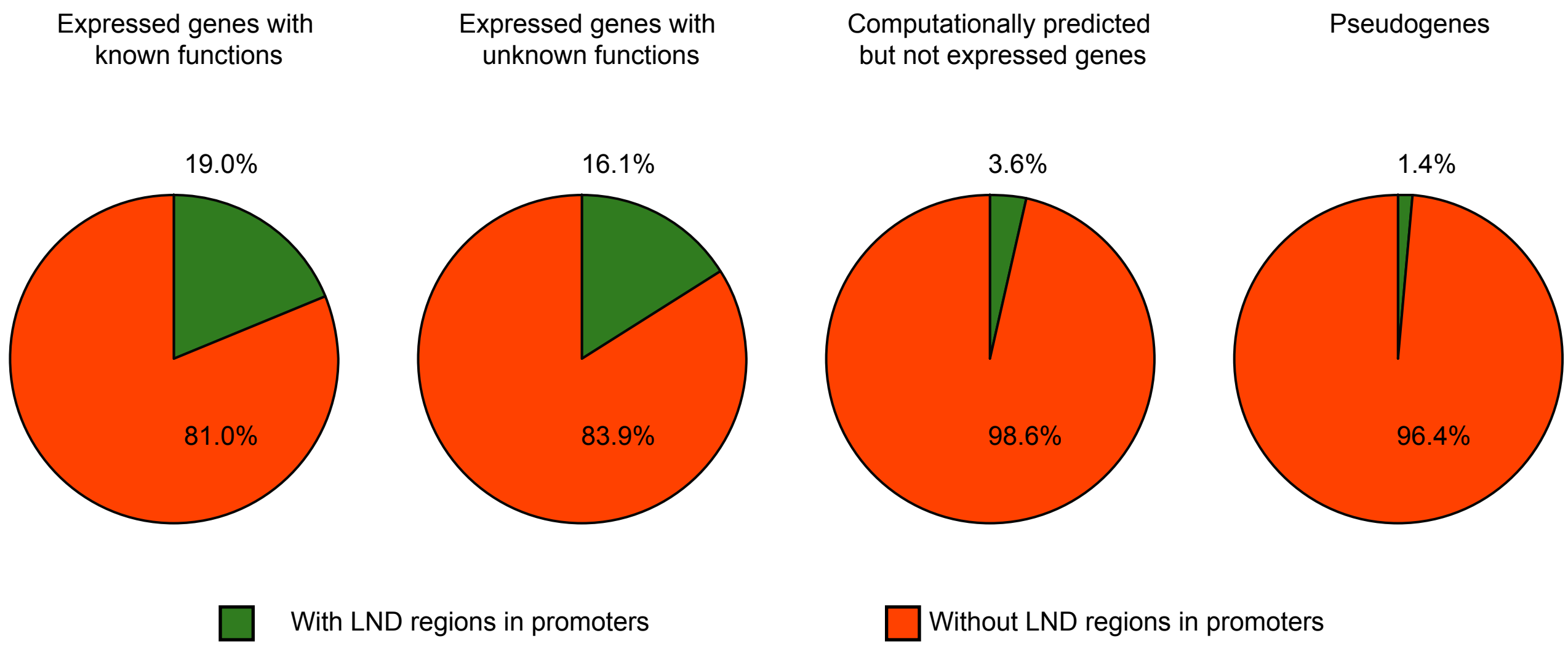

Supplemental Figure 8
