# Supplementary material for: Whole-Genome Analysis of Histone H3 Lysine 27 Trimethylation in Arabidopsis
Source: PLoS Biol. 2007 Apr 17;5(5):e129. doi: 10.1371/journal.pbio.0050129 (PMC1852588; doi:10.1371/journal.pbio.0050129)
Supplement: Supplementary file 13 [file pbio.0050129.st001.pdf]

Table S1. PCR primers used for validation of H3K27me3 results

Region in Figure S2	Chromosome	From	To	Forward Primer	Reverse Primer
1	2	17826400	17826638	GGCTCTCTGCTTTCATTTACTTT	ATTTGGTTGTGTTCAGAAATGG
2	2	17827684	17827888	GGGTGGTGCGAGTAATGAAG	GTAGAGACGGCCTCGAGTGG
3	2	17831870	17832091	CCTCAATTCCATTGTCAGCA	TGCCAGAACACATGTAGCAG
4	2	17834158	17834425	TGTGTGATGGAGTGGAAGGA	TCCGTAAGGTTTGAGGATGC
5	2	17835312	17835518	TTGCTATTTGATCTGGTATTCACC	TCTCTAAGCAAAAGTTTGATCTCATC
6	5	23039110	23039321	GGAATCAGAAATTCCAACAGG	TTGATGCTCTTCGTGCACTT
7	5	23040576	23040783	ACGAACCAATGCTGATGTGA	ACGCCAAAGTTCGACTTGAT
8	5	23042577	23042857	AAGCAATGCCGTATTGATCC	GCTGCACTTCAACAGCTTCA
9	5	23046464	23046679	TTTCACGCCAAGGGCTATAC	GATCCTGGAACCTCCATCAA
10	4	12497529	12497728	GAGAGTTCTGGCGAAACGAG	TGAGAAGGTTAAGAGGAAGATCAAG
11	4	12501040	12501280	GCTGCTTATTGGTTGGTCGT	GGTGGGTTCAATGTTTCGAT
12	4	12504534	12504817	AGGCCAGAAAAGGATCAAGC	CTCATGAGGTGGTGGTGATG
13	4	12508868	12509070	ACTTCACGATCCAGGAAACC	CAACCACCGCCTTCTTAAAC
14	4	12512014	12512289	TTGATGGGATGGATTGTGTG	AAGCGTTGACCTCATCAACC
