# Supplementary material for: Whole-Genome Analysis of Histone H3 Lysine 27 Trimethylation in Arabidopsis
Source: PLoS Biol. 2007 Apr 17;5(5):e129. doi: 10.1371/journal.pbio.0050129 (PMC1852588; doi:10.1371/journal.pbio.0050129)
Supplement: Supplementary file 14 [file pbio.0050129.st002.pdf]

Table S2. PCR primers used for validation of low nucleosome density regions

Region in Figure S5	In the promoter of	Forward Primer	Reverse Primer
1	At5g13100	CCTTTTTGAGATTCGGATGG	TGTGATCAGAGTAAAGATGGGTTT
2	At5g13230	CAAGTGTTTGGCTTTGGTCA	CAGGATAAACTGTTGGTGTTGG
3	At4g27260	TTGTTATGGACGGACACACC	AATACGCAAATCATTCCTCCA
4	At5g14210	GCCCTCTTTATGCCACAAGT	CCCAACCATATAATAGCATTTCTAA
5	At2g15520	AAAGACAACTGTCACCGTAAGAA	TCGACGTCAACTGCTAACGA
6	At5g10460	TGTCGTCTGCAAAGGAAAAA	AAAAATTCCTTCTGGACTCACG
7	At4g01330	CGCCCAAACCTTATCCTTTT	AAACCGGTGAACTAATGAATGA
8	At4g00640	TGGAAAATGACTTGGTCACG	ACTCGTACTCGCATGTGGTG
9	At3g07190	CACGCAGTTCCAAATTCCTT	TTCTCTACGGTCTACATGCTAACAA
10	At3g07390	GAGGTAGACGGGCATTGGTA	TTGGTTATGAGCCAAAATTCA
11	At2g24560	ACTCAGAGCCGACATTTGGT	CACGGGTCTGTCTAGTTCTCG
12	At5g14130	TTCATGGCGATCAGTCAAAA	AGTGTCGTTAAACACAAAATGGA
13	At1g24120	CCTCGATAAAGCCCAAGAAA	CAACGACGAGGACACTTCAA
14	At1g09780	TGACCATTTGAGATTTAAAAGAACT	TTTCAACTTTCAATATGACGAACC
15	At1g09860	TTCGATTCACACGAGTTTGC	CCGTAAAACCGACTTCAACC
16	At1g09940	GCATATTTTTCTTGGCTAAATTGG	GAAAAAGCCCATCAAAGCTG
17	At4g27320	TCCCGTACGTTGAGTTTGACT	GGAATTGACGATGTCGTGTG
